# Global priorities for an effective information basis of biodiversity distributions

**DOI:** 10.1038/ncomms9221

**Published:** 2015-09-08

**Authors:** Carsten Meyer, Holger Kreft, Robert Guralnick, Walter Jetz

**Affiliations:** 1 Biodiversity, Macroecology and Conservation Biogeography Group, Faculty of Forest Sciences, University of Göttingen, Büsgenweg 1, 37077 Göttingen, Germany; 2 University of Florida Museum of Natural History, University of Florida at Gainesville, 358 Dickinson Hall, Gainesville, Florida 32611-2710, USA; 3 Department of Ecology and Evolutionary Biology, Yale University, 165 Prospect Street, New Haven, Connecticut 06520, USA; 4 Department of Life Sciences, Imperial College London, Silwood Park Campus, Buckhurst Road, Ascot, Berks SL5 7PY, UK

## Abstract

Gaps in digital accessible information (DAI) on species distributions hamper prospects of safeguarding biodiversity and ecosystem services, and addressing central ecological and evolutionary questions. Achieving international targets on biodiversity knowledge requires that information gaps be identified and actions prioritized. Integrating 157 million point records and distribution maps for 21,170 terrestrial vertebrate species, we find that outside a few well-sampled regions, DAI on point occurrences provides very limited and spatially biased inventories of species. Surprisingly, many large, emerging economies are even more under-represented in global DAI than species-rich, developing countries in the tropics. Multi-model inference reveals that completeness is mainly limited by distance to researchers, locally available research funding and participation in data-sharing networks, rather than transportation infrastructure, or size and funding of Western data contributors as often assumed. Our results highlight the urgent need for integrating non-Western data sources and intensifying cooperation to more effectively address societal biodiversity information needs.

## Abstract (web summary)

Comprehensive digital information on species distributions is crucial for research in ecology, evolution and conservation. Here, Meyer *et al.* find large gaps and biases in global vertebrate point records, especially in emerging economies, and identify key factors currently limiting information.

## Graphical abstract

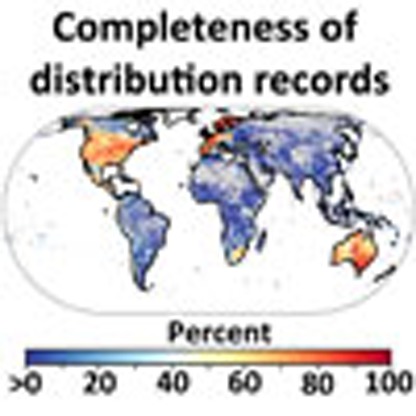

The parties to the Convention on Biological Diversity (CBD) have agreed on 20 targets to improve the state of biodiversity by 2020 ( https://www.cbd.int/sp/targets/). Aichi Target 19 specifically mandates the development of an advanced and shared biodiversity knowledge base. Information on species distributions in space is a central aspect of biodiversity knowledge that can enable the effective management of biodiversity and associated ecosystem services in a rapidly changing world^1^,^2^,^3^,^4^,^5^. Species distributions are critical for informing actions towards multiple Aichi targets, associated environmental indicators^6^ and the recently launched assessment work of the Intergovernmental science-policy Platform on Biodiversity and Ecosystem Services^7^.

International efforts to mobilize and aggregate distribution data, most notably through the Global Biodiversity Information Facility (GBIF), have facilitated access to large quantities of digital species occurrence records from a variety of data sources, especially museum specimens and field observations^8^,^9^. Such records provide vital, fine-scale information about where and when species occur and are widely used in ecology, evolution and conservation research. In contrast to expert knowledge or data sets that are either non-digital or not openly shared, and thus effectively inaccessible to most users, such mobilized records form the bulk of *de facto* ‘digital accessible information' (DAI, originally referred to as DAK in ref. 10). Although in a recent study^11^ the authors saw evidence for progress towards Aichi Target 19 in increasing volumes of GBIF-facilitated DAI, they had to acknowledge the critical caveat of unclear ‘taxonomic coverage (e.g., number of species), record completeness or geographic biases'.

Severe gaps and biases usually exist in DAI^10^,^12^,^13^,^14^ and these require careful consideration in ecological modelling^15^,^16^,^17^ and conservation research^3^. These data limitations may result from the way data are collected in the field, digitized in museums or mobilized and aggregated as digital species records into global biodiversity data-sharing networks. Different socio-economic and geographic drivers of data limitations have been hypothesized, including inadequate financial and institutional resources^18^,^19^,^20^, poor international scientific cooperation^20^, lack of access or regional safety concerns^20^,^21^,^22^,^23^, or a focus on regions with certain appeal like endemism-, species-rich or protected areas^12^,^21^,^24^.

The amount of data required to completely inventory species assemblages is a function of their richness and the spatial grain^13^,^14^,^25^. To be relevant for conservation applications, distribution data sets must inform about species occurrences at fine spatial grains^26^, either directly or by facilitating derived, fine-grain models^5^,^13^. Such fine-grain models are integral to conservation research, but can also directly influence conservation decision-making. For instance, occurrence records have facilitated the identification of ‘priority areas'^27^ in Madagascar, where following a legal decree, no mining and forestry activities can be permitted (*Arrêté Interministériel* n18633/2008/MEFT/MEM, renewed in 2014; further examples in ref. 5).

Identifying information gaps and factors limiting the dissemination of biodiversity information are recognized as priorities both at the political^28^ and scientific^29^ levels of the CBD. To date, magnitude and exact location of gaps in global DAI as well as the generality and relative importance of underlying causes remain unclear, hampering prioritization of future data mobilization efforts^30^. International efforts to mobilize biodiversity records remain un-assessed for their success and effectiveness in addressing targets to improve and share biodiversity knowledge.

Here we perform this assessment for 21,170 species of birds, mammals and amphibians, and c. 157 million geographically and taxonomically validated point records that were provided to GBIF by 160 data publishers, including small institutions with a distinct taxonomic and geographic focus, large internationally active research museums and citizen science programmes. We determine the factors currently limiting biodiversity inventory completeness in global DAI and identify priority regions and activities to advance it. We find that most gaps in inventories exist in large emerging economies and DAI is mainly limited by distance to data contributors, locally available research funding and political commitment to data sharing. To advance global DAI effectively, efforts to foster participation in data-sharing networks and mobilize non-Western data sources should be prioritized.

## Results and Discussion

### Patterns in global DAI on species distributions

**Figure 1:**
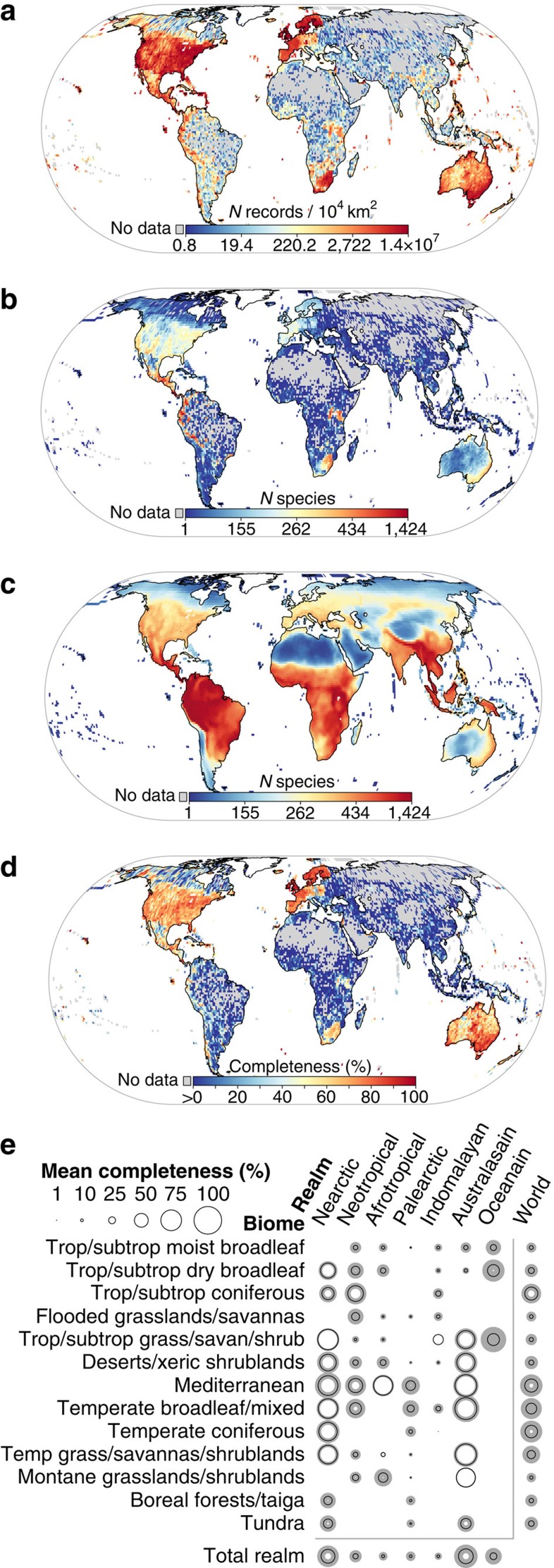
Global unevenness and gaps in the DAI on distributions of 21,170 species of terrestrial vertebrates (birds, mammals and amphibians). (**a**) Density of point records, (**b**) species richness from point records, (**c**) species richness from expert opinion and (**d**) inventory completeness (percentage of expected richness documented by records). Grey areas do not have any mobilized records. (**e**) Mean inventory completeness in biome-realm combinations. Size of black circles is proportional to mean inventory completeness and grey areas show s.d. All assessed over a 110-km equal-area grid.

At a grain size of 110 km grid cells, the density of terrestrial vertebrate records varies by five orders of magnitude (Fig. 1a), peaking in parts of Europe, North and Central America and Australia. Conversely, 48% of Asian, 35% of African and 21% of South American cells have no records mobilized into DAI. At this spatial grain, the finest ensuring sufficient accuracy of species expert-range maps^31^,^32^, species richness derived from point records shows little concordance with expected richness (Fig. 1b,c). Although spatial patterns between the two data sources show at least weak associations (*r*_s_=0.28–0.39, see Supplementary Table 1a), only 4.2% of all 12,029 cells reach ≥80% completeness (Fig. 1d).

Completeness, defined as percentage of expected richness documented with point records, is moderately to strongly predicted by record density (binomial generalized linear model (GLM), *d*^2^=0.59–0.90, Supplementary Fig. 1, Supplementary Table 1b and see Supplementary Notes 1–3 for details). Whereas high record density results in high levels of completeness in much of the Nearctic and Australasia, this is less the case for the more species-rich Neo- and Afrotropics (Fig. 1a,b,d,e and Supplementary Fig. 1D). The Eastern Palaearctic and Indomalayan realms are characterized by particularly low levels of completeness. Average completeness also varies greatly among the world's major biomes and biomes within biogeographical realms (Fig. 1e and Supplementary Table 2a–c). Specifically, tropical and subtropical forests, grasslands and savannas, but also boreal forests and tundra biomes remain vastly underinventoried. Surprisingly, we cannot confirm a pronounced ‘tropical data gap'^33^ (_max_-*t* test, *P*_Dut_=0.27, *N*=4,717/7,286; tropics versus non-tropics). Instead, a severe gap emerges across most of Asia (including temperate regions), non-Southern Africa and Brazil (_max_-*t* test, *P*_Dut_<0.01, *N*=6,089/5,914; when comparing mean completeness in these areas to all others; see also Supplementary Tables 2 and 3).

**Figure 2:**
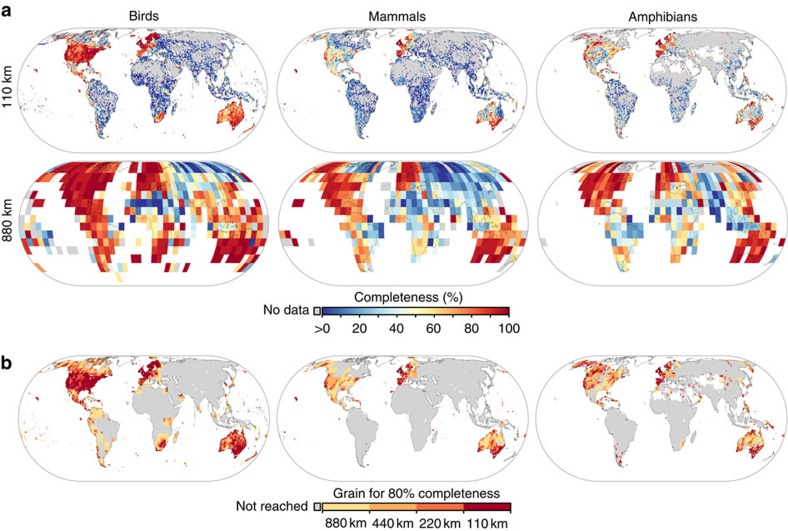
Spatial variation in point record-based inventory completeness for three vertebrate taxa at different spatial grains. (**a**) Inventory completeness at the 110- and 880-km grain. (**b**) Minimum grain size to reach 80% inventory completeness, mapped at 110 km. Grey grid cells (**a**) show areas within the taxon's global range without mobilized records and (**b**) areas that do not reach 80% completeness at 880 km.

Although these strong geographic differences in completeness are broadly repeated among the three vertebrate groups (Fig. 2a), completeness patterns among the three taxa only show moderately strong positive associations (*r*_s_=0.65–0.74 depending on taxon and grain, _max_-*t* tests, all *P*_Dut_<0.001, *N*=323–11,522). This suggests that the completeness pattern of a single-taxon is a poor predictor for un-assessed taxa and highlights the need to identify taxon-specific information gaps^34^. As expected from substantially fewer records for mammals and amphibians compared with birds (∼3 and ∼1 M compared with ∼150 M, see Supplementary Table 4), their overall level of completeness is significantly lower (Tukey's test, all *P*_Dut_<0.001, *N*=280–11,757, depending on spatial grain, when comparing mammal/amphibian completeness with bird completeness).

Completeness levels of ≥80% over large extents, even at a relatively coarse grain of 110 km, are only achieved in birds and only in North America, Europe and Australia (Fig. 2a). Coarsening grains even further to 440 or 880 km substantially increases completeness in all groups (Kruskal–Wallis test, all *P*<0.001, *N*=280–11,757, Fig. 2a,b and Supplementary Fig. 2), but necessarily leads to inferior opportunities for inference and application. Such coarse grains are not adequate for most questions in ecology^35^ and, with land-use and conservation actions typically set at the kilometer scale or finer, are unsuited for effective resource management. Most species distribution models (SDMs) connecting records with fine-grained environmental data for extrapolation^17^ are unable to provide a general remedy here, owing to their known sensitivity to environmental bias^14^,^36^. This pervasive lack of DAI over vast extents (for example, only <20% completeness at 880 km grain over much of Asia, Fig. 2a) demonstrates that for many regions with large conservation opportunities^37^ there are not sufficient mobilized occurrence data to facilitate even the most sophisticated modelling approaches. Global numbers of sampling locations for the majority of species are far below the 50–100 typically recommmended^3^,^38^,^39^ as minimum SDM requirements (54.9% of all bird species have <50 records, median=37; mammals: 79.2%, median=6; amphibians: 91.3%, median=2) (compare refs 14, 40).

### Addressing information gaps effectively

**Figure 3:**
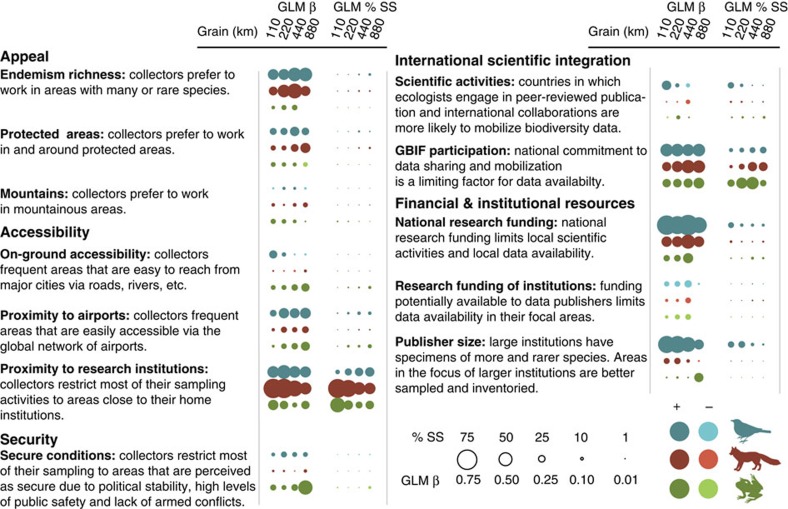
Determinants of inventory completeness in DAI on species distributions. Effects were tested in multiple generalized linear regression models with a binomial distribution and a logit link (GLM β and GLM % SS). All possible model subsets were ranked based on AIC scores and subsets with ΔAIC<10 re-run as spatial models to account for spatial autocorrelation in model residuals. Bubble size represents the relative strength of predictor–response relationships. Vertebrate groups are represented by different colours, with shading denoting the direction of the relationship. We show the relative importance of predictors using two different metrics: (i) the standardized coefficients of the reduced spatial multiple regression models with the lowest AIC score (blank cells indicate variables that were not included in these models) (GLM β), and (ii) the percentage each predictor has in the total sum of squares (GLM % SS) of a type III analysis of variance.

Such glaring data gaps highlight the need to identify and, where possible, address the root causes of low inventory completeness. Understanding of the key driving factors of bias is important to prioritize activities in data mobilization. Further, drivers of bias can be explicitly incorporated into biodiversity models^41^,^42^. To this end, we tested 12 hypotheses falling into 5 broad categories: appeal, accessibility, security, international scientific integration, and financial and institutional resources (details in Fig. 3 and Supplementary Notes 2 and 3, Supplementary Figs 3–6 and Supplementary Table 5). Most hypotheses receive at least some support in our multi-model inference framework, highlighting the complex interplay of geographic and socio-economic factors as drivers of inventory completeness (Fig. 3; for record density and bivariate model results, see Supplementary Fig. 5; detailed results in Supplementary Tables 6–8). Depending on taxon and grain, minimum adequate models of inventory completeness explain 60%–78% of the deviance (Supplementary Table 6) and the relative importance of factors varies more strongly among taxonomic groups than among grain sizes (depending on the predictor, percentages of sums of squares explained in an analysis of variance are 16.5%–72.5% higher for factor ‘taxon' compared with factor ‘spatial grain').

A strong role for data collection has been attributed to region or species ‘appeal', for example, researchers' preference for reserves, mountains or other areas of high total, rare and range-restricted species richness^21^,^24^,^43^. We find this supported in birds and mammals by strong positive effects on inventory completeness of endemism richness and weaker effects of protected area coverage. Surprisingly, we find relatively low importance of on-ground accessibility from cities and proximity to airports (Fig. 3), which have previously been suggested to strongly constrain field collections^21^,^23^. In contrast, spatial distance to data-contributing institutions (Supplementary Table 9) consistently emerges as a key predictor of inventory completeness and record density (Fig. 3 and Supplementary Fig. 5). This highlights the imprint that long-term logistics of maintaining field sampling and specimen transport leave on global biodiversity information (compare refs 22, 24). Insecure conditions may discourage field sampling^20^,^44^, but we find little evidence that security aspects are important in limiting completeness or record density (Fig. 3, Supplementary Fig. 5 and Supplementary Note 2). We expected our index of integration into scientific activities, that is, country's H-index in ecology multiplied by level of international collaboration, to be strongly correlated with inventory completeness, as it should reflect the routine of making research results public^20^,^33^. However, it is not an important factor for explaining completeness or record density (Fig. 3 and Supplementary Fig. 5). Conversely, GBIF participation emerges as a consistently strong factor determining completeness in DAI. Supporting previous suggestions^19^,^45^, national research funding (gross expenditure on research and development) is strongly positively correlated with completeness (Fig. 3). Surprisingly, however, research funding of countries where data-publishing institutions are situated does not affect inventory completeness in the regions of their sampling activity (Supplementary Note 2). Finally, publisher size, estimated from contributed data volume, only weakly predicts inventory completeness for mammals and amphibians, but it has much stronger effects for birds, where the largest data contributors are not museums but aggregators of citizen-science observations (Supplementary Table 9), pointing to the potential of alternative, non-institution-based ways of producing DAI for certain taxa (see discussions in refs 13, 46, 47).

Most of the strongest limiting factors of completeness affect digitization and mobilization of existing data rather than the actual collection of new records in the field. Although adequate national research funding is vital for producing DAI on local biodiversity, our results suggest that funding for university research usually leading to peer-reviewed publications is not improving our ability to close information gaps as greatly as direct support for data mobilization programmes (Fig. 3: ‘Scientific activities' versus ‘GBIF participation'). A likely reason is that current data-archiving policies^48^ and academic reward systems^49^ do not favour data-sharing activities. They further suggest that the largest or best-funded museums alone are unable to guarantee high inventory completeness in distant regions, unless their efforts are backed by supportive local conditions, such as locally available research funding, mobilization efforts in local research institutions and national commitment to data sharing. The most effective strategy for closing gaps in DAI may therefore lie in supporting mobilization efforts in institutions nearby identified data gaps and supporting participation in international data-sharing programmes. Dedicated funds and specialized personnel for data mobilization in developed, often low-diversity countries may be better applied to support efforts in countries that lag behind, due to lack of expertise or cyber infrastructure^50^, for example, through direct partnerships or capacity building assistance.

**Figure 4:**
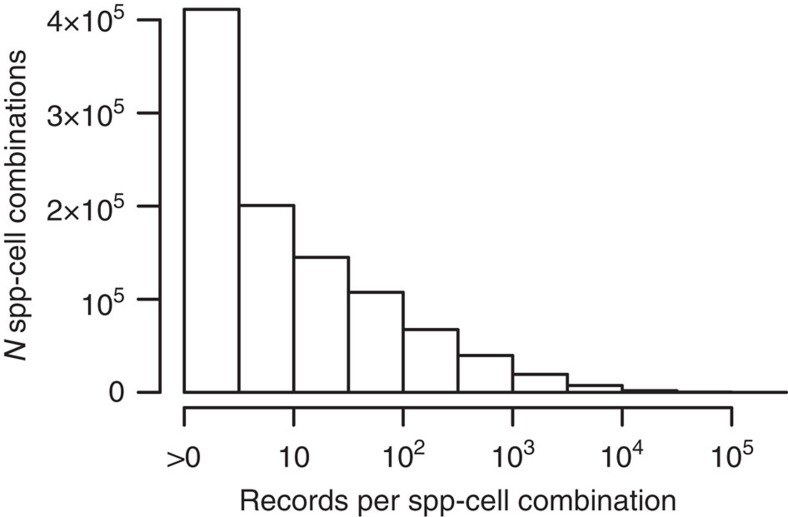
Redundancy of information in 157 M globally mobilized point records that constitute DAI on species distributions. The histogram shows the frequency of different degrees of information duplication (duplicated species-grid cell combinations) at the 110-km grain. Theoretically, and under ideal sampling, representing each of 3.7 M species-grid cell combinations by one record would achieve 100% inventory completeness at that spatial grain.

The need to mobilize more data to increase completeness is obvious: 69%–95% of the deviance in completeness explained by our minimum adequate models can also be explained by differences in record density (Supplementary Table 7a). However, we find that there is much room for improving the effectiveness of such mobilization: representing each known species of the three vertebrate groups once in every 110 km cell within its range, and thus achieving 100% inventory completeness globally at that spatial grain, would require c. 3.7 M ideally sampled records. Currently, about 42 times that many (157 M) validated records represent only 21.6% (0.8 M) of these 3.7 M unique species-grid cell combinations, demonstrating a huge level of informational redundancy concentrated in a few places (Fig. 4, compare ref. 47). Such intensive but localized sampling and data mobilization may benefit local conservation efforts as well as many purely scientific endeavors, but surely trades off against global-scale data needs, such that gaps in DAI are particularly severe in regions where higher-resolution data sets are most needed to support cost-effective progress towards multiple Aichi Targets^37^,^51^. Strategic mobilization of data sources that likely contain many missing species-grid cell combinations could prove effective in quickly closing gaps and reducing geographical bias in global DAI. This in turn would facilitate robust, fine-grain distribution models from SDM or downscaling approaches^52^ for a greater and geographically more representative sample of species than previously possible^3^, and could immediately support various Aichi Targets^6^. Examples include land-use planning to minimize biodiversity loss (Target 7), creating species lists for protected areas and improving global reserve networks (Target 11), safeguarding threatened species (Target 12) and mapping and securing associated ecosystem services (Target 14). Targeting sufficiently recent data sources would furthermore create strong synergies with keeping conservation assessments up-to-date^53^. As a concrete example of potential conservation impacts, GBIF-facilitated records were recently used in the legal listing of five species of sawfish (Pristidae) under the US Endangered Species Act^54^. Increased access to occurrence information alone cannot ensure sound application nor conservation outcomes, but it can facilitate sound, data-driven decision-making^5^, which in many parts of the world is currently impossible. We therefore argue that data mobilization efforts should be coordinated and strive to maximize return-on-investment for global conservation applicability.

**Figure 5:**
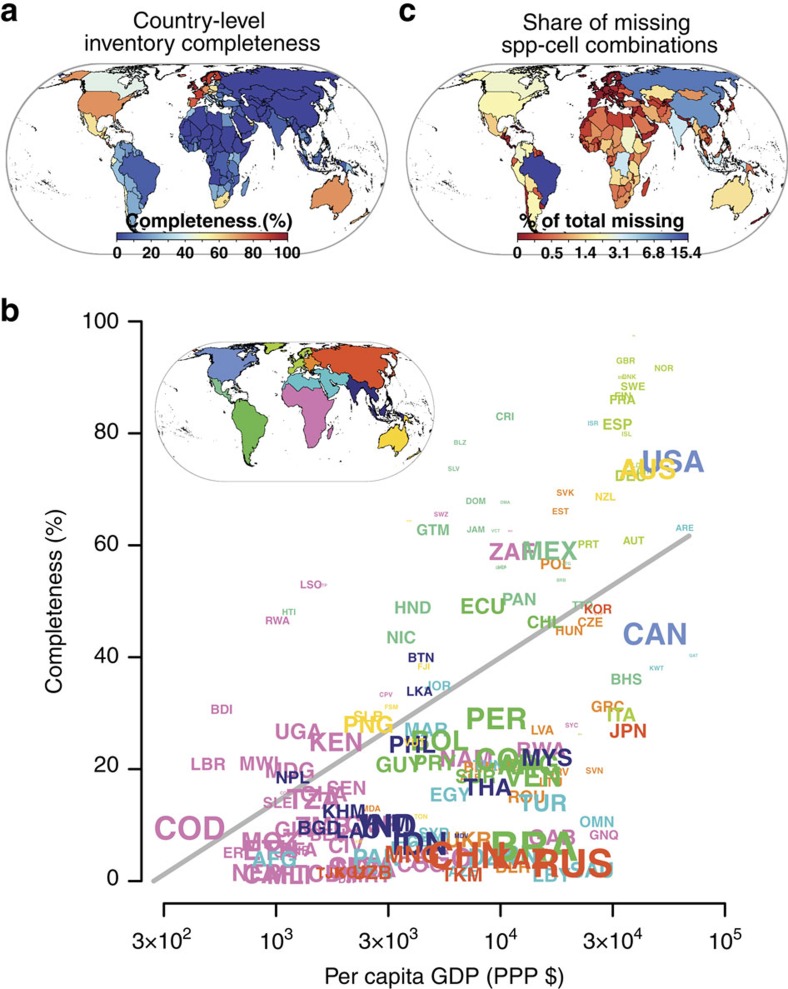
Gaps in DAI on species distributions at the country level. (**a**) Country-level inventory completeness, measured as the percentage of the total unique species-grid cell combinations in each country that are covered by GBIF records. (**b**) Country-level inventory completeness in relation to per capita gross domestic product (in purchase power parity dollars, PPP $); *r*^2^=0.34, *P*<0.001. Font size of country ISO codes is proportional to the total number of unique species-grid cell combinations that need to be recorded in each country to reach 100% inventory completeness at the 110-km grain. Font colour is for geographical reference (compare inset map). Countries mentioned in the main text: BRA, Brazil; CHN, China; IDN, Indonesia; IND, India; MEX, Mexico; RUS, Russia; TUR, Turkey. (**c**) Share that each country has in the unique species-grid cell combinations that are missing globally from a complete inventory at the 110-km grain.

Immediate opportunities for addressing gaps in DAI are most apparent at the national level: we find that even after controlling for all investigated factors (which explain 92.1%–97.2% of cross-national variation), country identity still explains a significant portion of inventory completeness (2.4%–7.1% of *D*^2^; Supplementary Table 7b), pointing to an important role of country-specific political, legal, historical, linguistic or cultural factors (Supplementary Note 4). If countries were equally committed to providing access to their biodiversity information, as agreed upon by CBD signatories, completeness should be mainly limited by available financial resources. However, there is only a moderate relationship between country-level completeness and per capita gross domestic product (*r*^2^=0.34, *P*<0.001; Fig. 5a,b) or total conservation spending^55^ (*r*^2^=0.16, *P*<0.001). Notably, several large emerging economies including Brazil, China, India, Indonesia, Russia or Turkey lag behind (Fig. 5b,c and Supplementary Table 3), which is worrying given increasing pressure on their biodiversity from rising global and domestic consumption^56^,^57^. Success in building an adequate information basis for global biodiversity conservation and thus globally informed policies for environmental sustainability will depend on their support and may be determined by political rather than economic factors. For example, despite the large mobilization needs owing to its megadiverse biota, Mexico has a leading role in biodiversity informatics due to early political support for establishment of a national biodiversity programme^58^. Data-rich institutions in economically powerful countries such as Brazil, China and Russia^12^,^14^,^24^, which together account for 31% of missing species-grid cell combinations (Fig. 5c and Supplementary Table 3), seem particularly well-poised to contribute significantly to globally accessible species distribution information.

As countries such as Brazil recently announced intentions to relax biodiversity research restrictions^59^, as well as to improve and unlock their data store, existing national programmes (for example, speciesLink; http://splink.cria.org.br) will increasingly be integrated into global DAI, and information gaps and priorities may rapidly shift. More than current snapshots, tools for ongoing re-evaluation (see http://patterns.mol.org/completeness) may aid researchers to assess or account for data bias^60^ as well as monitor progress in data mobilization^11^.

This global cross-taxon assessment represents a first in a number of steps required for more effective understanding and confrontation of information gaps on species distributions. Although terrestrial vertebrates represent only c. 1.6% of described species^61^, addressing the factors that emerged as important across vertebrate taxa may hold the greatest promise for closing gaps for biodiversity in general. Vitally, and confirmed by the strong taxon dependence of our results, assessments of distribution information need to be extended to more species-rich groups such as fishes, plants and invertebrates (for example, see refs 10, 23, 25 for regional assessments). Comparing ratios between mobilized record volumes and described species numbers suggests that gaps in DAI may be one to three orders of magnitude more severe in those groups (average records per species: tetrapods (31,032 spp.): 6,909; fishes (31,658 spp.): 347; vascular plants (283,701 spp.): 317; invertebrates (1.38M spp.): 31; numbers of geo-referenced records from GBIF website, June 2014, species numbers from ref. 61).

Such profound data limitations call for more holistic solutions. Our assessment highlights potential ways for making institution-based data mobilization more effective, but also the limitations of such efforts. Point records from biocollections only represent one of a variety of data sources^13^ and their targeted mobilization should be complemented by other ways to address biodiversity information needs. Thorough biodiversity assessments led by trained field biologists will continue to play an important role in the creation of primary information for unsurveyed, biodiverse areas. In addition, novel approaches such as citizen science projects are already providing increasingly valuable records for certain taxa at comparatively low cost^46^. Improved reward systems^49^ and new data publishing mechanisms and journal requirements^48^ can incentivize both individual scientists and larger project teams to openly share biodiversity records. Much information held by conservation non-governmental or governmental organizations may be unlocked through supportive mechanisms, such as stronger evaluation and attribution of progress towards declared national commitments (for example, Aichi Target 19) and more widely adopted strategies to address sensitive information, for example, on threatened species^62^.

Further opportunities for improvements lie in better use of available information. Novel Bayesian modelling approaches can address some of the typical limitations of classical SDMs, for example, by connecting different data types across spatial scales^52^ or by explicitly modelling bias-causing processes^41^,^42^,^63^. Geographically or thematically focused data platforms such as eBird^46^ or Atlas of Living Australia^62^ have already highlighted the opportunities of using enriched information together with models. Novel biodiversity informatics infrastructure such as Map of Life^13^ has the potential to provide an integration of disparate information sources, and to link these with environmental information through best-suited modelling tools to address species distributions and their changes globally.

Rapid biodiversity loss, limited funding and potential trade-offs with direct conservation investments^64^ require priorities for future collection and mobilization of biodiversity records into DAI. Targeted integration of available information and assessments of gaps, along with continued evaluation of effectiveness of DAI for conservation needs, are as vital as increased commitment to biodiversity data sharing by political stakeholders, institutions and individual scientists. With time running out to meet CBD targets on biodiversity knowledge, more effective data use and mobilization, and a cultural shift about data sharing are urgently needed.

## Methods

### Species distribution data

We overlaid expert-based extent-of-occurrence range maps for terrestrial birds (excluding pelagic feeders; *N*=9,712), terrestrial mammals (*N*=5,270) and amphibians (*N*=6,188) with four nested equal-area grids (grain sizes: 110, 220, 440 and 880 km) to infer coarse-resolution species richness patterns. As a representation of international efforts to collect, digitize and share biodiversity records, we compiled a database of nearly 200 M records for the three groups, aggregated by GBIF (see Supplementary Tables 4 and 9, and Supplementary Note 1). We focus on GBIF given that it is by far the largest such effort in geographic and taxonomic scope^8^,^9^ and has an intergovernmental mandate to openly make accessible data from a worldwide base of data publishers. Data from GBIF represent the greatest body of existing DAI on species occurrences, based on centuries' worth of museum specimens, citizen science observations, surveys, literature and other sources. GBIF also has a vital role in sharing skills, software, tools and best practices for biodiversity data mobilization. Thus, GBIF-facilitated DAI is currently the best available indicator of ‘shared biodiversity knowledge, science base and technologies' as referred to by Aichi Target 19 (ref. 11). To link GBIF-facilitated records with range maps, extensive taxonomic standardization was necessary (our approach as well as various filtering and validation steps are explained in the Supplementary Note 1). We defined inventory completeness as the percentage of expert-opinion species richness documented by mobilized records. We note that other DAI sources play vital and often complementary roles in progressing towards Aichi Targets (Supplementary Note 4). Yet, other data sets may not be shared but nevertheless influence regional research and conservation. Thus, results here should not be interpreted as definite maps of knowledge gaps, but the analyses of drivers are likely indicative of factors limiting biodiversity information in other data sources.

### Geographic and socio-economic drivers of gaps in DAI

We analysed relationships of 12 geographic and socio-economic factors with record density and inventory completeness. We used three variables to describe the appeal of areas to attract collectors: (i) endemism richness^65^, that is, the sum of inverse range sizes of all species present in a grid cell, was calculated from the number of 110 km cells. (ii) To model effects of mountains on record collection, we calculated the topographic range in each cell based on a digital elevation model. (iii) We modelled the effects of protected areas using proportions of land area in grid cells that fall within protected areas of International Union for Conservation of Nature categories I–IV. We investigated three aspects of accessibility: (i) to test for effects of on-ground accessibility, we used a data set on the time needed to travel to cities with a population **>**50,000 (ref. 66). (ii) To model effects of the proximity to airports, we created an index based on the locations of >9,300 airports and airfields^67^. (iii) ‘Proximity to institutions' was expressed as weighted geographic proximity of a grid cell to those data publishers that contributed records for the area surrounding the cell. Index values are high if the majority of records are contributed by geographically close data publishers. We modelled effects of secure conditions using the Global Peace Index^68^, which aggregates information on political stability, armed conflicts and levels of public safety. We investigated two aspects of international scientific integration: (i) to quantify integration into ‘scientific activities', we extracted the H-index for every country based on peer-reviewed papers published in the field ‘Ecology, Evolution, Behavior and Systematics' from *Elsevier*'s *Scopus* database (covering the years 1996–2011), and multiplied it with the proportion of papers resulting from international collaborations (see Supplementary Note 2). (ii) We tested for effects of political commitment to data sharing using the proportion of the land area within each grid cell that falls within GBIF-participating countries. We used three measures of financial and institutional resources: we estimated financial resources that are potentially available for biodiversity research from per capita gross domestic expenditure on research and development (i) within grid cell-overlaying countries (‘National research funding') as well as (ii) in countries where the publishers of records for a particular cell are situated (‘Research funding of institutions'). (iii) We used record volumes contributed to GBIF by different data publishers to estimate institution size. Details on calculation and transformation of predictor variables, along with detailed information on the respective hypotheses and the limitations of our data sources are in Supplementary Notes 2 and 4.

### Statistical methods

We investigated effects of predictor variables on inventory completeness separately for amphibians, birds and mammals at each of the four spatial grains with simple and multiple regressions. Specifically, we used non-spatial and spatial generalized linear models with a binomial distribution, where completeness enters as a composite variable (‘species covered by records', ‘species not covered but presumed present') and where differences in species richness are automatically accounted for. Spatial models account for residual spatial autocorrelation by including a ‘residuals autocovariate' built from residuals of the non-spatial model and an optimized spatial neighbourhood structure^69^. Because of long computation times for spatial models, we ran all possible non-spatial models and re-ran those model subsets that would likely be among the minimum adequate spatial models (with ΔAIC <10 to the lowest Akaike Information Criterion score) as spatial models. We assessed model fits of minimum adequate spatial models as the % deviance explained (*D*^2^) (Supplementary Table 6). We investigated interactions among variables as well as nonlinear effects, but—although many were significant—accounting for them did not greatly alter model fit or parameter estimates of main effects in preliminary analyses. To maintain as much simplicity as possible given 12 predictor variables and 12 separate sets of models (3 taxa × 4 spatial grains), we decided to focus on the main effects. We used standardized coefficients (*β*) of minimum adequate spatial models (with the lowest AIC scores) to measure the relative importance of predictor variables. As an alternative measure, we used percentages of the sums of squares attributable to each factor, based on analyses of variance with a response variable consisting of the AIC scores of all possible models and predictor variables coding the presence/absence of each predictor in the respective model. As we modelled effects separately for each of the three vertebrate groups, the over-representation of birds in terms of species and record number does not bias the conclusions for mammals and amphibians (Supplementary Note 4). We identified factors that are most important for limiting inventory completeness by focusing on those effects that consistently emerged as important across vertebrate groups, grains sizes and evaluation metrics. For further details and references, see Supplementary Notes. *P*-values were adjusted to geographically effective degrees of freedom following Dutilleul^70^.

### Data archiving

The synonym table used for this study as well as data sets used to plot maps and run regression models are available as Supplementary Data 1 and 2.

## Additional information

**How to cite this article:** Meyer, C. *et al.* Global priorities for an effective information basis of biodiversity distributions. *Nat. Commun.* 6:8221 doi: 10.1038/ncomms9221 (2015).

## Supplementary Material

Supplementary Information Supplementary Figures 1-6, Supplementary Tables 1-9, Supplementary Notes 1-4 and Supplementary References.
