# Supplementary material for: Global priorities for an effective information basis of biodiversity distributions
Source: Nat Commun. 2015 Sep 8;6:8221. doi: 10.1038/ncomms9221 (PMC4569846; doi:10.1038/ncomms9221)
Supplement: Supplementary Information — Supplementary Figures 1-6, Supplementary Tables 1-9, Supplementary Notes 1-4 and Supplementary References. [file ncomms9221-s1.pdf]

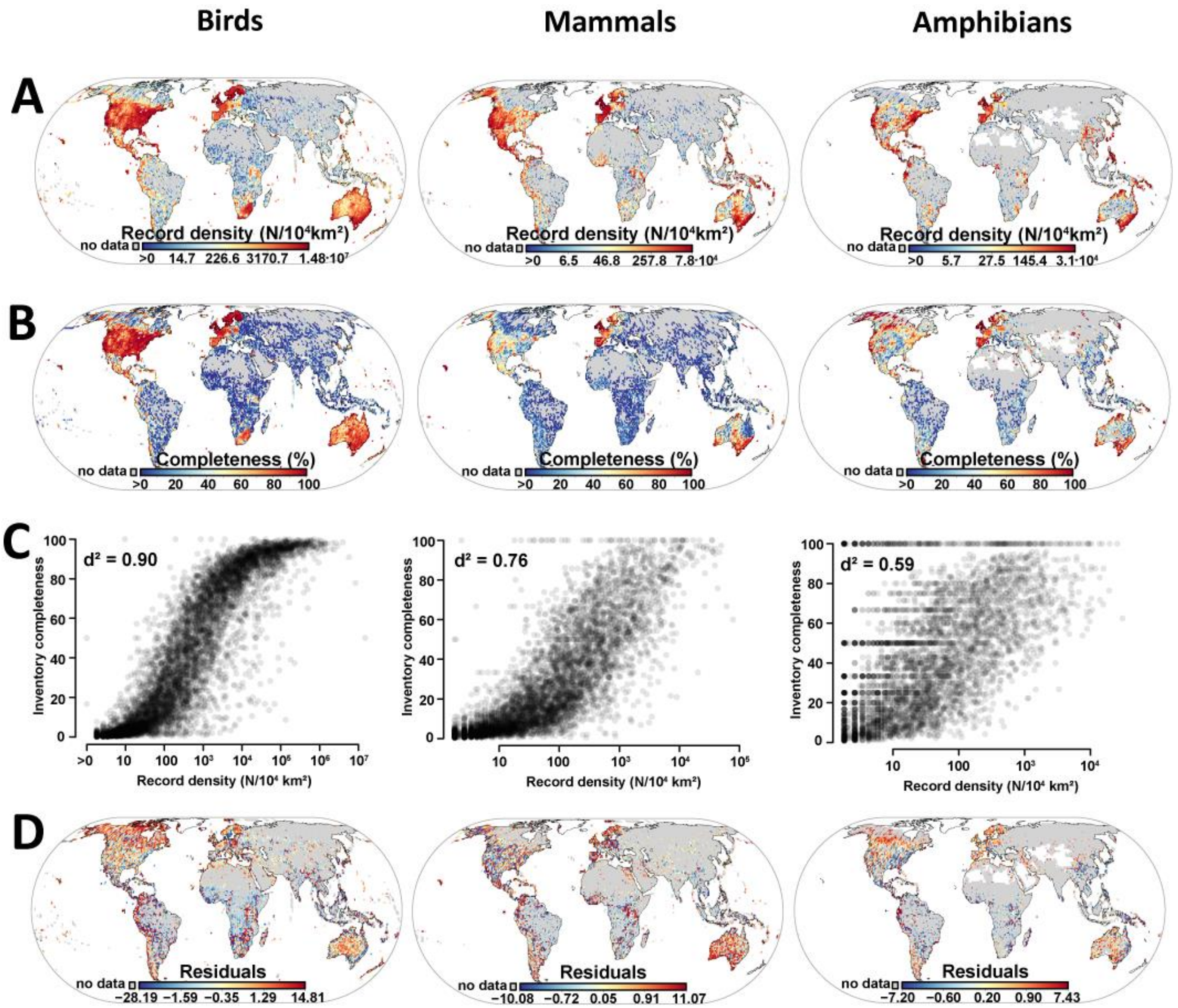

Supplementary Fig. 1. Relationships between record density and inventory completeness in global 'digital accessible information' for three vertebrate groups at the 110 km grain. A) Record density, B) Inventory Completeness, C) Scatter plots of relation between inventory completeness and record density with deviance explained (d^2) based non non-zero grid cells, D) Spatial arrangement of residuals of a binomial generalized linear model (logit link) explaining inventory completeness with record density. Red values indicate higher, blue values lower inventory completeness than expected from record density.

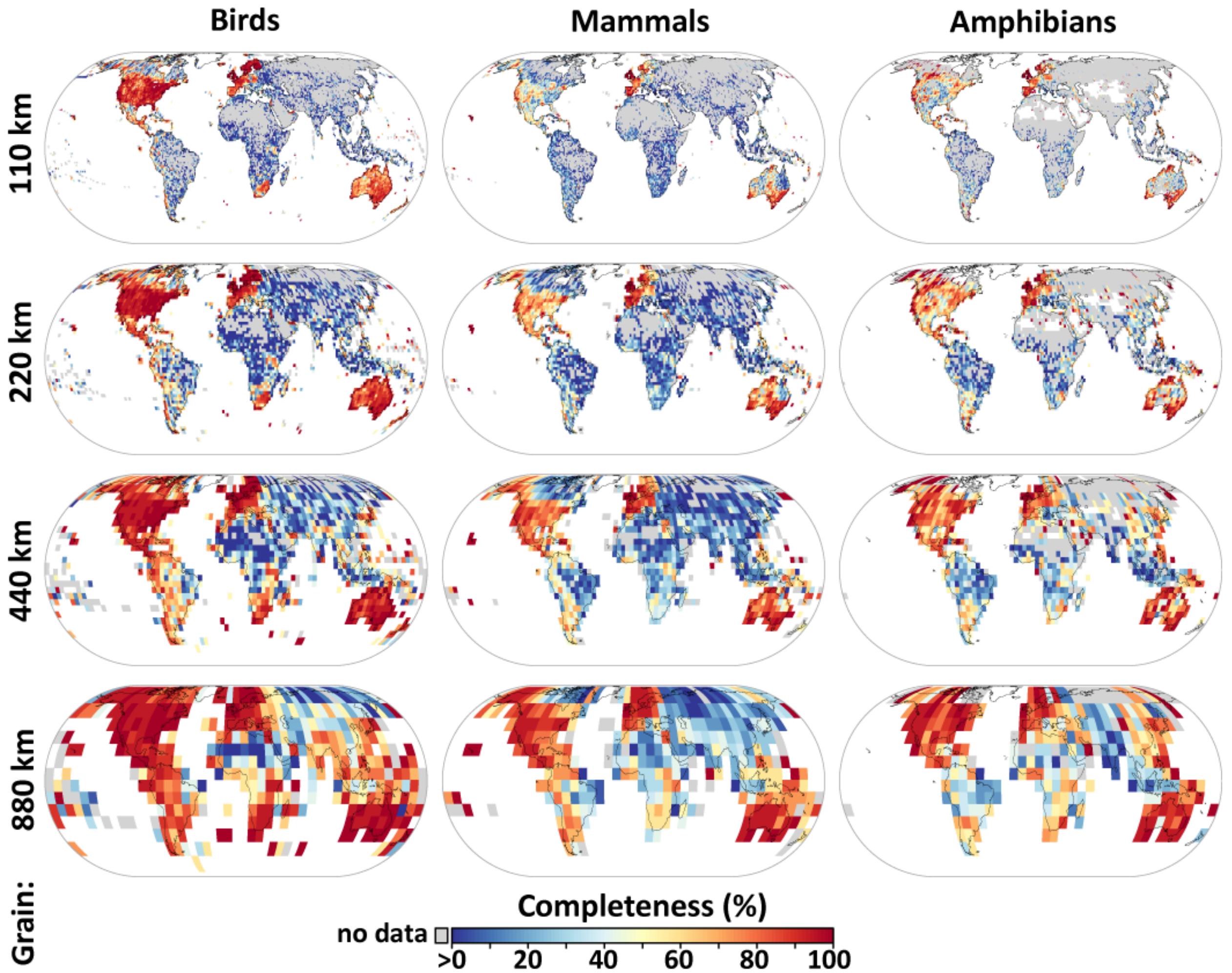

Supplementary Fig. 2: Spatial variation in record-based inventory completeness for three vertebrate taxa at four spatial grains. Grey grid cells show areas within the global range of the taxonomic group with no mobilized records.

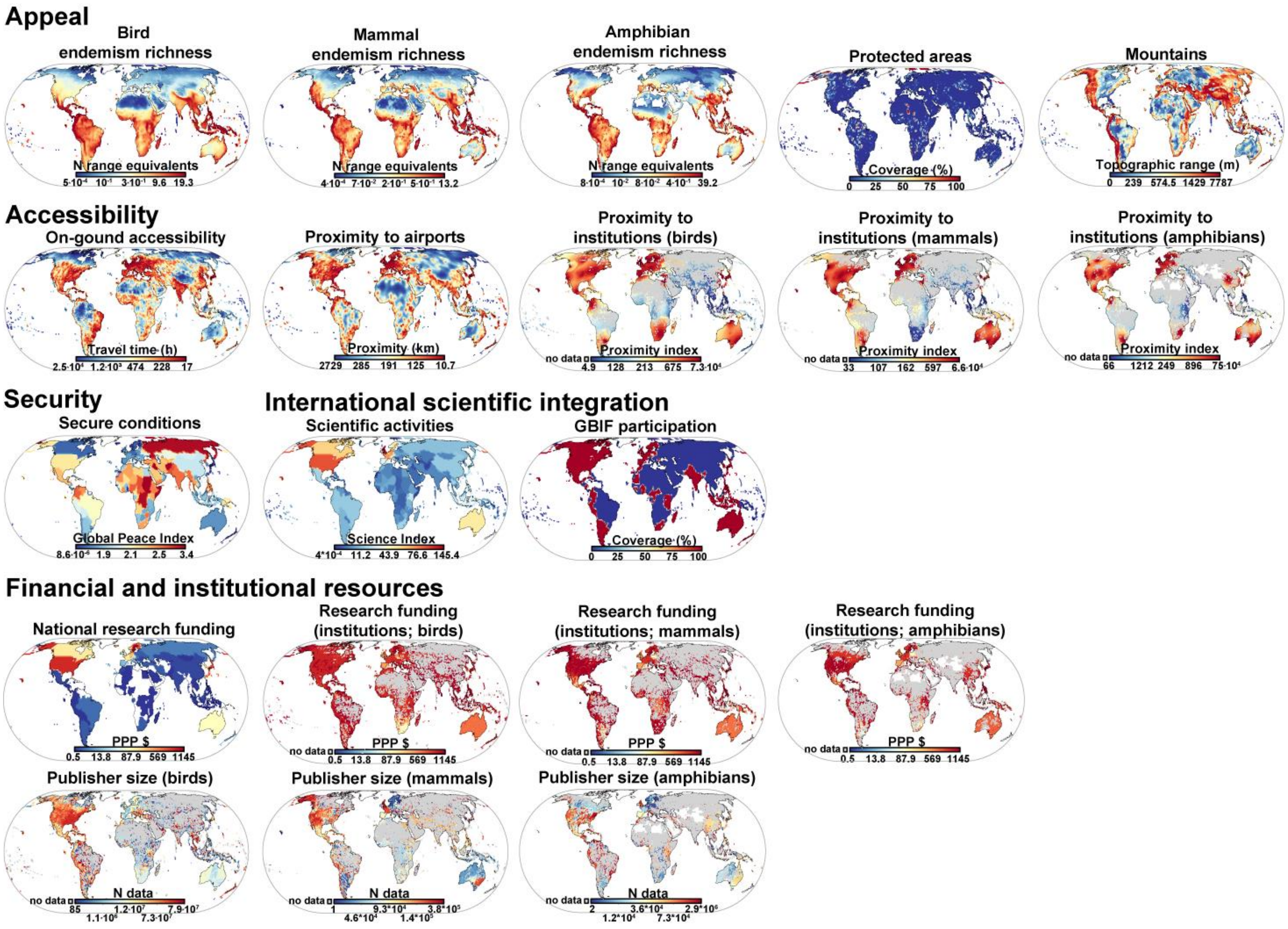

Supplementary Fig. 3: Maps of predictor variables used to model point record density and inventory completeness (110 km grain). For details on variables and source datasets see 'Materials and Methods'. The Global Peace Index (variable 'Secure conditions') has high values for insecure conditions and we multiplied values with -1 to test for effects of secure conditions. The variable 'Research funding (institutions)' describes the mean research funding of the countries where the providers of records for a given grid cell are situated. The variable 'Publisher size' describes the mean size (contributed data volume) of the providers of records for a given grid cell.

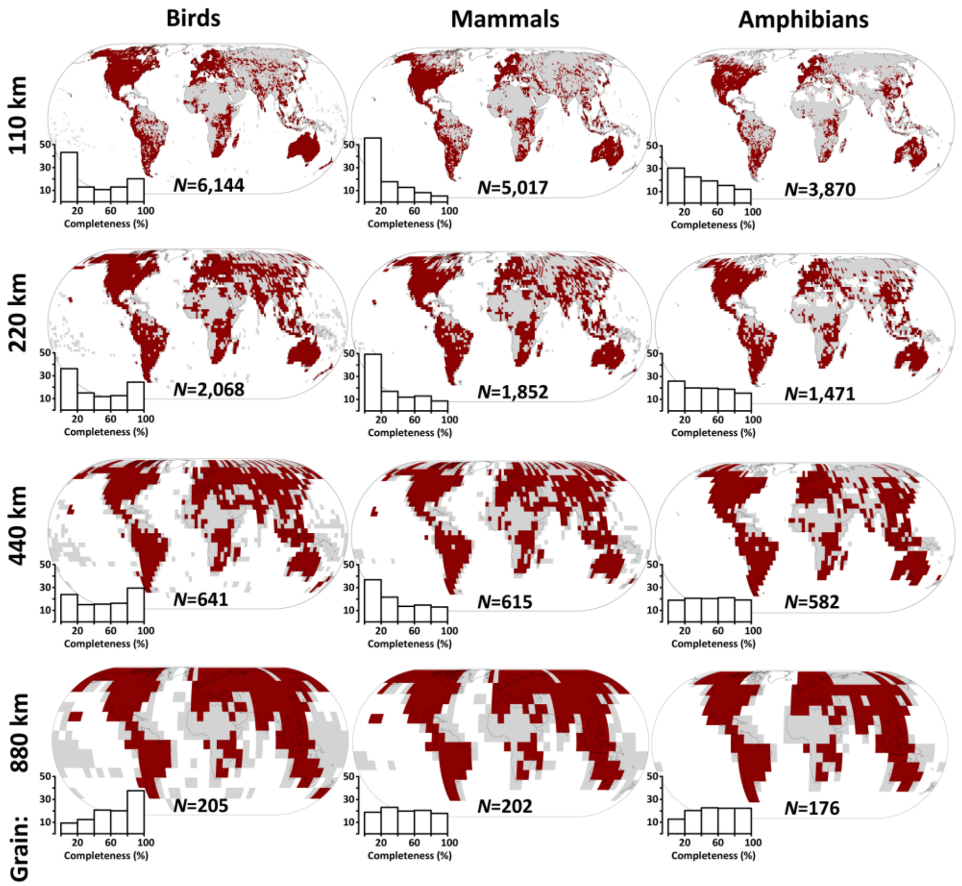

Supplementary Fig. 4: Grid cells selected for models of point record density and inventory completeness. Dark red cells were considered in models, grey cells were not considered although the taxonomic group is present because they either had no records or no data for all predictor variables was available. At the bottom part of each map the number of grid cells in the respective models (*N*) is shown. Inset histograms show percentages of included grid cells in five 20%-completeness classes.

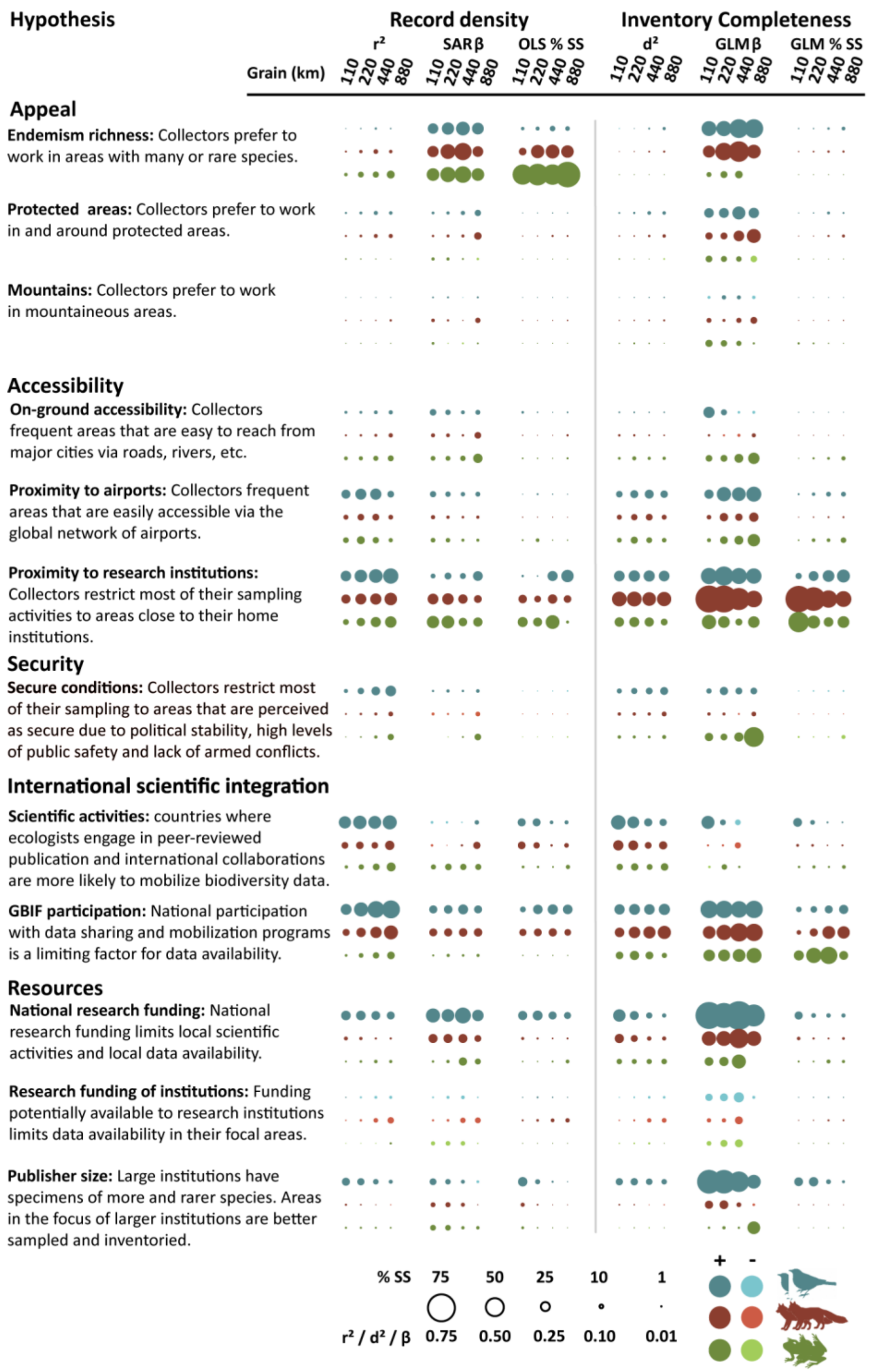

Supplementary Fig. 5: Determinants of point record density and inventory completeness. Effects were tested in simple and multiple regression models. All model subsets were ranked based on AIC scores and subsets with ΔAIC <10 re-run as spatial models, by accounting for spatial autocorrelation in model residuals. For record density, we used ordinary least squares models and simultaneous autoregressive models (SAR β and OLS % SS). For inventory completeness, we used spatial and non-spatial generalized linear models with a binomial distribution and a logit link (GLM β and GLM % SS). Bubble size represents the strength of predictor-response relationships. Vertebrate groups

are represented by color, with shading denoting the direction of the relationship. We show predictor strength for record density using three different metrics: i) the coefficient of determination in simple regressions (r^2), ii) the standardized coefficients of the reduced subset of the spatial multi-predictor model with the lowest AIC score (blank cells indicate variables that were not included in these models) (SAR β), and iii) the percentage each predictor has in the total Sums of Squares (OLS % SS) of a type III ANOVA. For the latter we used AIC values of all possible model subsets as the response variable and dummy-variables coding whether or not a predictor is in the respective model as explanatory variables. We show predictor strength for inventory completeness using three different metrics analogous to those for record density: i) the deviance explained in simple generalized linear regression models (d^2), ii) the standardized coefficients of the reduced spatial multiple generalized linear regression models with the lowest AIC score (GLM β), and iii) the percentage each predictor has in the total Sums of Squares (GLM % SS) of a type III ANOVA.

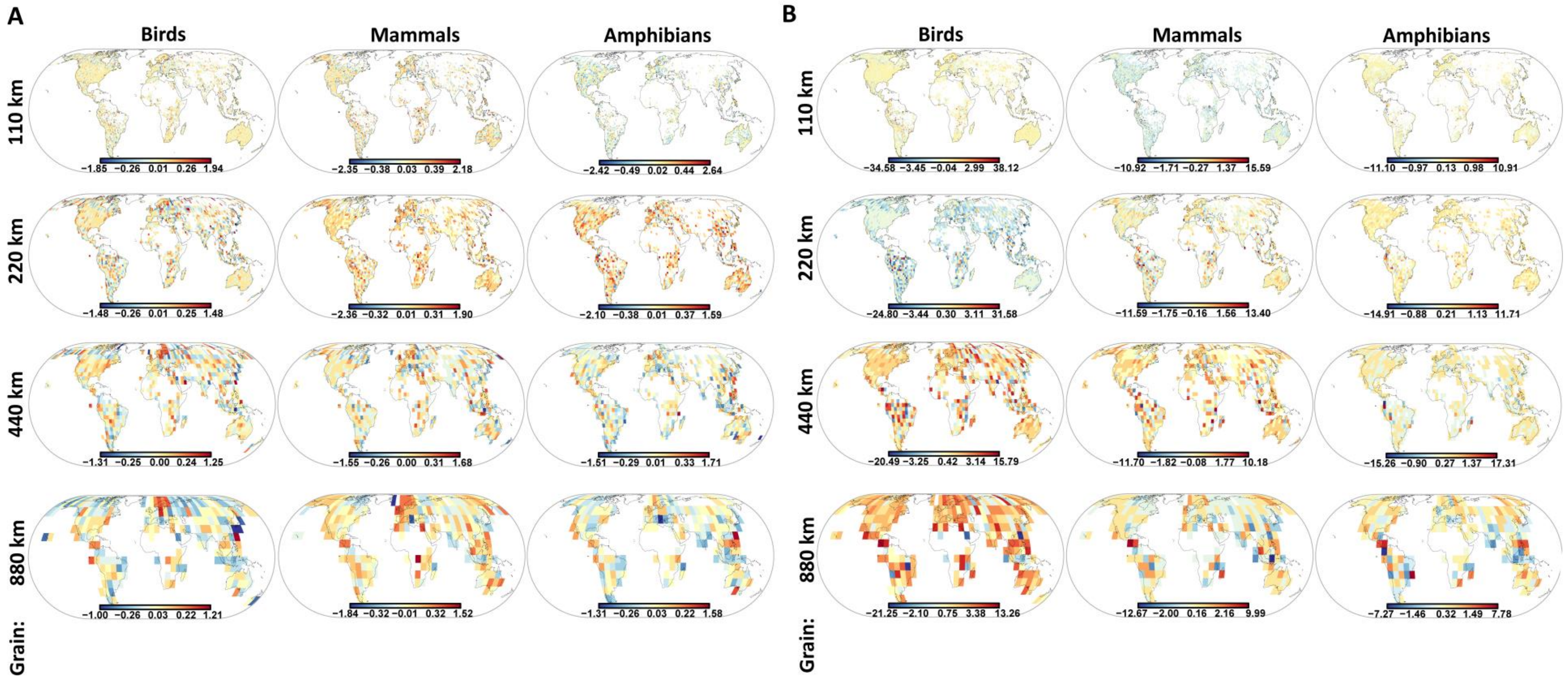

Supplementary Fig. 6: Spatial arrangement of residuals in **A)** simultaneous autoregressive models of point record density and **B)** spatial binomial generalized linear models (logit link) of inventory completeness. Red values indicate higher, blue values lower inventory completeness than expected from the predictor variables.

Supplementary Table 1

a) correlations between record density and inventory completeness

	Grain (km)	median record density	median inventory completeness	r_S	*N* cells
**Birds**	110	8.61	0.03	0.91***	7,378
	220	48.11	0.22	0.89***	2,863
	440	115.87	0.47	0.85***	1,007
	880	304.46	0.65	0.78***	350
**Mammals**	110	0.81	0.01	0.82***	5,885
	220	5.66	0.08	0.84***	2,447
	440	14.76	0.24	0.87***	888
	880	33.39	0.43	0.84***	300
**Amphibians**	110	0.00	0.00	0.57***	4,346
	220	1.83	0.16	0.57***	1,863
	440	4.13	0.36	0.56***	699
	880	13.81	0.50	0.60***	251

b) correlations between GBIF richness and expert richness

	Grain (km)	median $SR_{records}$	median SR_{expert}	r_S	*N* cells
**Birds**	110	4	193	0.35**	11,757
	220	34	205	0.58***	3,575
	440	83.5	228.5	0.79***	1,136
	880	157	274.5	0.91***	372
**Mammals**	110	1	52	0.28*	11,522
	220	5	57	0.49***	3,415
	440	16	69	0.69***	1,037
	880	39	92	0.83***	323
**Amphibians**	110	0	10	0.39***	10,002
	220	2	12	0.61***	2,973
	440	5	16	0.81***	919
	880	14	29	0.91***	280

Supplementary Table 1: Global correlations between **a)** record density and inventory completeness (based on grid cells with at least one record) and **b)** species richness evident in mobilized occurrence point records ($SR_{records}$) and expected true species richness based on expert-opinion range maps (SR_{expert}). For each taxonomic group and spatial grain (km), the median record density (N records/10^4 km²), the median inventory completeness, the Spearman's rank coefficient (r_s), and the number of grid cells (N cells) are shown. Asterisks behind r_s represent *P*-values corrected for spatial autocorrelation[80] (.: *P*<0.1; *: *P*<0.05; **: *P*<0.01; ***: *P*<0.001).

Supplementary Table 2

a) Variation among biomes

Biome	*N* cells	Min	Max	Mean	SD	Median
Tropical & Subtropical Moist Broadleaf Forests	2,214	0.0	100.0	14.1	20.2	3.2
Tropical & Subtropical Dry Broadleaf Forests	374	0.0	96.7	22.7	23.8	14.6
Tropical & Subtropical Coniferous Forests	62	0.4	80.9	46.7	21.8	51.2
Flooded Grasslands & Savannas	75	0.0	86.9	13.1	20.3	1.5
Tropical & Subtropical Grasslands, Savannas & Shrublands	1,637	0.0	100.0	14.4	23.5	1.7
Deserts & Xeric Shrublands	2,369	0.0	96.3	17.8	27.5	0.7
Mediterranean Forests, Woodlands & Scrub	325	0.0	96.1	47.6	31.0	52.2
Temperate Broadleaf & Mixed Forests	1,129	0.0	96.1	38.7	34.6	32.3
Temperate Conifer Forests	320	0.0	88.6	45.2	31.7	58.6
Montane Grasslands & Shrublands	410	0.0	72.1	11.8	19.8	1.5
Temperate Grasslands, Savannas & Shrublands	830	0.0	100.0	29.9	32.0	11.3
Boreal Forests/Taiga	1,317	0.0	94.1	15.9	25.5	0.5
Tundra	775	0.0	100.0	20.5	26.3	3.9

b) Variation among realms

Realm	*N* cells	Min	Max	Mean	SD	Median
Nearctic	1,727	0.0	94.1	49.9	25.6	58.8
Neotropics	1,715	0.0	86.9	19.8	23.2	8.9
Afrotropics	1,817	0.0	100.0	10.6	18.1	1.6
Palearctic	4,539	0.0	96.1	10.0	22.2	0.0
Indomalay	890	0.0	80.0	9.6	14.4	2.1
Australasia	985	0.0	96.3	53.1	29.3	62.3
Oceania	178	0.0	100.0	22.8	31.0	0.0

c) Variation among biome-realm combinations

Biome	Realm	*N* cells	Min	Max	Mean	SD	Median
Tropical & Subtropical Moist Broadleaf Forests	Australasia	261	0.0	92.9	16.7	20.5	5.3
	Neotropics	799	0.0	86.5	16.8	22.3	4.6
	Afrotropics	311	0.0	79.5	11.7	16.8	3.7
	Palearctic	44	0.0	19.6	4.0	4.7	2.8
	Indomalay	645	0.0	80.0	10.2	15.2	2.2
	Oceania	154	0.0	100.0	19.6	29.3	0.0
Tropical & Subtropical Dry Broadleaf Forests	Nearctic	3	45.7	67.3	54.5	11.4	50.5
	Oceania	19	0.0	96.7	43.0	32.3	47.4
	Neotropics	175	0.0	83.6	32.6	24.3	31.5
	Australasia	32	0.0	30.2	12.2	9.3	13.6
	Afrotropics	23	0.0	69.9	19.4	23.5	7.4
	Indomalay	122	0.0	51.0	8.1	11.8	2.0
Tropical & Subtropical Coniferous Forests	Neotropics	32	6.6	80.9	55.3	20.2	60.0
	Nearctic	22	16.8	74.4	44.2	16.7	43.2
	Indomalay	8	0.4	40.0	19.3	16.3	20.4
Flooded Grasslands & Savannas	Neotropics	23	0.0	86.9	29.1	26.0	26.3
	Indomalay	2	0.8	24.4	12.6	16.7	12.6
	Palearctic	19	0.0	36.7	5.2	10.4	0.7
	Afrotropics	31	0.0	45.1	6.1	12.6	0.5
Tropical & Subtropical Grasslands, Savannas & Shrublands	Nearctic	8	67.9	86.4	74.2	5.6	73.0
	Australasia	192	2.1	92.3	64.9	13.8	65.3
	Indomalay	1	36.6	36.6	36.6	-	36.6
	Oceania	5	0.0	100.0	43.3	46.5	33.3
	Neotropics	275	0.0	65.3	9.2	13.8	2.3
	Afrotropics	1,156	0.0	72.5	6.7	13.5	0.8
Deserts & Xeric Shrublands	Australasia	297	20.3	96.3	65.9	14.6	67.6
	Nearctic	198	3.7	87.3	59.8	16.9	64.4
	Afrotropics	214	0.0	72.2	19.8	22.5	9.2
	Neotropics	125	0.0	84.3	20.7	25.1	8.1

		**Variation among biome-realm combinations (continued)**						
**Biome**	**Realm**	***N* cells**	**Min**	**Max**	**Mean**	**SD**	**Median**
	Indomalay	90	0.0	43.9	5.6	10.1	0.7
	Palearctic	1,445	0.0	71.4	2.4	7.7	0.0
Mediterranean Forests,Woodlands & Scrub	Australasia	67	50.9	93.0	78.6	10.3	81.7
	Nearctic	17	7.7	88.1	71.4	20.6	78.9
	Afrotropics	8	57.5	78.7	69.8	6.2	71.2
	Neotropics	15	0.0	81.3	51.3	21.8	54.7
	Palearctic	218	0.0	96.1	35.1	28.7	27.2
Temperate Broadleaf & Mixed Forests	Australasia	73	0.0	94.4	79.1	16.5	82.4
	Nearctic	236	9.4	87.5	70.3	9.7	71.5
	Neotropics	43	0.0	79.5	42.2	25.9	47.5
	Indomalay	13	0.0	40.8	17.0	16.6	9.1
	Palearctic	764	0.0	96.1	25.2	32.0	6.9
Temperate Conifer Forests	Nearctic	192	3.9	85.8	66.5	14.8	70.9
	Palearctic	127	0.0	88.6	13.5	22.1	1.9
	Indomalay	1	0.2	0.2	0.2	-	0.2
Temperate Grasslands, Savannas & Shrublands	Australasia	49	48.4	90.4	76.8	10.3	79.1
	Nearctic	249	12.7	87.9	67.1	11.0	68.9
	Afrotropics	5	0.0	100.0	45.9	49.7	15.5
	Neotropics	144	0.0	75.2	18.2	18.1	10.6
	Palearctic	383	0.0	44.3	4.0	8.3	0.4
Montane Grasslands & Shrublands	Australasia	6	66.2	72.1	68.7	2.0	68.5
	Afrotropics	66	0.0	70.1	34.4	27.9	32.7
	Neotropics	62	0.0	63.8	22.7	16.6	21.2
	Palearctic	276	0.0	39.7	2.7	5.6	0.3
Boreal Forests/Taiga	Nearctic	438	0.0	94.1	30.4	23.6	26.3
	Palearctic	879	0.0	91.6	8.6	23.3	0.0
Tundra	Australasia	8	0.0	64.3	37.4	21.0	41.7
	Nearctic	364	0.0	89.1	32.8	23.4	31.8
	Palearctic	384	0.0	94.4	8.1	22.1	0.0

d) Variation among countries

**GeoRegion**	**Country**	**Code**	***N* cells**	**Min**	**Max**	**Mean**	**SD**	**Median**
South America	Ecuador	ECU	30	0.0	84.3	52.6	22.3	58.6
	Falkland Islands (Islas Malvinas)	FLK	11	0.0	59.6	35.7	20.3	41.8
	Chile	CHL	76	0.0	81.3	36.9	25.5	39.0
	Peru	PER	108	0.0	78.3	29.6	20.3	31.2
	Bolivia	BOL	86	0.0	64.4	23.3	16.6	22.2
	Suriname	SUR	11	0.0	50.1	17.7	15.2	20.6
	Guyana	GUY	20	0.3	65.3	20.1	16.7	18.6
	French Guiana	GUF	6	0.0	29.0	15.1	11.8	18.3
	Paraguay	PRY	31	0.5	67.3	19.5	16.1	17.9
	Uruguay	URY	16	0.6	51.3	20.2	16.7	16.0
	Colombia	COL	98	0.0	68.4	19.0	19.2	13.4
	Venezuela	VEN	80	0.0	64.6	17.7	17.8	10.9
	Brazil	BRA	704	0.0	54.1	5.0	9.9	0.5
Central America/Caribbean	British Virgin Islands	VGB	1	79.9	79.9	79.9	-	79.9
	Puerto Rico	PRI	6	30.8	82.4	70.2	19.7	77.4
	Costa Rica	CRI	6	35.7	86.5	69.9	19.2	76.2
	Belize	BLZ	2	75.3	76.9	76.1	1.1	76.1
	El Salvador	SLV	2	67.7	75.5	71.6	5.5	71.6
	Virgin Islands	VIR	1	70.0	70.0	70.0	-	70.0
	Dominican Republic	DOM	6	64.0	73.2	67.7	3.6	66.4
	Dominica	DMA	1	64.9	64.9	64.9	-	64.9
	Guatemala	GTM	10	34.5	78.2	62.1	12.2	64.6
	Jamaica	JAM	5	53.9	73.3	62.3	7.3	61.8
	St. Vincent and the Grenadines	VCT	1	60.6	60.6	60.6	-	60.6
	Cayman Islands	CYM	3	47.2	84.3	64.0	18.8	60.5
	Netherlands Antilles	ANT	1	57.3	57.3	57.3	-	57.3
	St. Lucia	LCA	1	56.1	56.1	56.1	-	56.1
	Antigua and Barbuda	ATG	1	55.7	55.7	55.7	-	55.7
	Grenada	GRD	1	55.0	55.0	55.0	-	55.0
	Mexico	MEX	182	0.0	87.3	52.4	19.5	54.8
	Panama	PAN	11	10.2	76.5	45.8	23.3	53.8
	Barbados	BRB	1	53.7	53.7	53.7	-	53.7
	Haiti	HTI	3	36.5	58.3	47.5	10.9	47.8
	Martinique	MTQ	2	42.9	49.7	46.3	4.8	46.3
	Honduras	HND	12	0.0	61.2	42.9	17.9	46.0

Variation among countries (continued)

GeoRegion	Country	Code	*N* cells	Min	Max	Mean	SD	Median
	Trinidad and Tobago	TTO	2	34.1	57.6	45.8	16.7	45.8
	St. Kitts and Nevis	KNA	1	43.9	43.9	43.9	-	43.9
	Montserrat	MSR	1	41.0	41.0	41.0	-	41.0
	Nicaragua	NIC	13	0.0	63.6	37.5	21.5	42.7
	Cuba	CUB	16	1.1	61.2	36.8	15.9	40.4
	Guadeloupe	GLP	2	0.0	78.3	39.1	55.3	39.1
	Bahamas, The	BHS	22	0.0	80.9	33.9	26.5	33.4
	Anguilla	AIA	1	24.5	24.5	24.5	-	24.5
	Turks and Caicos Islands	TCA	4	0.0	39.7	17.6	20.2	15.3
Northern America	United States	USA	848	0.0	100.0	64.4	18.9	69.8
	Bermuda	BMU	1	45.5	45.5	45.5	-	45.5
	Canada	CAN	827	0.0	85.6	35.5	24.7	35.0
North/West Europe	Ireland	IRL	9	87.3	96.1	92.9	2.8	93.5
	Denmark	DNK	7	81.5	90.8	85.3	3.3	84.6
	Sweden	SWE	41	73.5	90.2	84.0	3.8	84.4
	Finland	FIN	30	74.2	91.6	84.1	4.4	84.2
	United Kingdom	GBR	33	16.7	94.2	83.6	14.5	88.1
	Norway	NOR	28	65.1	90.0	83.3	5.3	84.0
	Belgium	BEL	2	82.5	85.1	83.8	1.8	83.8
	France	FRA	49	66.4	89.1	79.9	5.2	81.3
	Spain	ESP	61	0.0	96.1	71.0	19.6	75.4
	Germany	DEU	29	41.1	81.5	68.8	10.4	71.1
	Switzerland	CHE	4	49.7	76.2	67.0	11.8	71.0
	Iceland	ISL	11	51.4	80.3	68.3	8.9	69.4
	Netherlands	NLD	3	62.3	81.8	70.4	10.1	67.3
	Austria	AUT	5	16.1	68.1	56.9	22.8	67.0
	Portugal	PRT	17	0.0	72.2	49.5	21.6	55.6
	Malta	MLT	1	26.2	26.2	26.2	-	26.2
	Italy	ITA	35	0.0	51.1	24.9	14.2	22.9
	Svalbard	SJM	29	0.0	94.4	27.4	29.1	17.6
	Greenland	GRL	13	0.0	65.5	17.8	21.3	9.5
	Faroe Islands	FRO	4	0.0	36.8	12.7	16.8	7.0
	Jan Mayen	SJM	3	0.0	11.1	3.7	6.4	0.0
East/South-East Europe	Estonia	EST	5	9.7	85.9	64.5	31.3	75.4
	Slovakia	SVK	4	62.4	68.5	65.4	2.5	65.4
	Poland	POL	27	16.0	85.7	55.1	19.7	60.9
	Hungary	HUN	7	11.4	63.3	42.8	21.3	49.8
	Cyprus	CYP	1	45.3	45.3	45.3	-	45.3
	Czech Republic	CZE	8	19.5	71.7	44.3	21.8	39.6
	Latvia	LVA	7	2.3	48.3	26.1	15.0	31.7
	Greece	GRC	14	12.0	47.3	30.0	12.0	31.2
	Bosnia and Herzegovina	BIH	13	0.5	36.2	18.9	13.9	24.6
	Croatia	HRV	3	12.4	21.2	17.8	4.7	19.9
	Macedonia	MKD	2	13.4	23.9	18.7	7.4	18.7
	Montenegro	MNE	1	18.0	18.0	18.0	-	18.0
	Slovenia	SVN	2	14.6	19.7	17.2	3.6	17.2
	Bulgaria	BGR	9	0.0	36.6	18.7	13.2	16.4
	Lithuania	LTU	5	6.5	32.7	17.1	10.6	13.6
	Moldova	MDA	2	9.5	14.9	12.2	3.8	12.2
	Albania	ALB	4	2.3	40.3	16.6	16.7	11.8
	Romania	ROU	20	0.3	45.8	13.7	13.5	10.8
	Ukraine	UKR	49	0.3	65.3	6.6	11.9	1.2
	Byelarus	BLR	20	0.0	22.8	2.2	5.2	0.4
Australia/Oceania	Wake Island	UMI	1	100.0	100.0	100.0	-	100.0
	Norfolk Island	NFK	1	92.9	92.9	92.9	-	92.9
	Nauru	NRU	1	80.0	80.0	80.0	-	80.0
	Australia	AUS	660	2.1	96.3	69.4	14.6	70.9
	Western Samoa	WSM	2	36.1	94.1	65.1	41.0	65.1
	New Zealand	NZL	40	0.0	88.1	54.9	24.9	65.0
	Guam	GUM	1	47.4	47.4	47.4	-	47.4
	Northern Mariana Islands	MNP	7	0.0	94.4	42.3	32.9	33.3
	Papua New Guinea	PNG	82	0.0	61.6	21.1	18.3	20.1
	Solomon Islands	SLB	29	0.0	73.7	22.8	21.9	18.0
	Niue	NIU	1	15.4	15.4	15.4	-	15.4
	New Caledonia	NCL	18	0.0	78.7	20.6	27.3	4.0
	Cook Islands	COK	14	0.0	88.9	21.0	32.0	0.0
	French Polynesia	PYF	30	0.0	44.0	9.0	13.8	0.0
	Kiribati	KIR	29	0.0	66.7	6.0	15.8	0.0
	Micronesia, Federated States of	FSM	38	0.0	100.0	18.0	34.3	0.0

Variation among countries (continued)

GeoRegion	Country	Code	*N* cells	Min	Max	Mean	SD	Median
	Pitcairn Islands	PCN	2	0.0	0.0	0.0	0.0	0.0
	Tokelau	TKL	3	0.0	0.0	0.0	0.0	0.0
	Tonga	TON	11	0.0	55.6	11.0	21.5	0.0
	Tuvalu	TUV	4	0.0	0.0	0.0	0.0	0.0
	US Minor Outlying Islands	UM	4	0.0	0.0	0.0	-	0.0
	Wallis and Futuna	WLF	3	0.0	0.0	0.0	0.0	0.0
Tropical Asia	Cocos (Keeling) Islands	CCK	1	50.0	50.0	50.0	-	50.0
	Bhutan	BTN	3	35.5	38.8	37.3	1.7	37.6
	Sri Lanka	LKA	7	3.0	50.4	32.5	17.8	37.6
	British Indian Ocean Territory	IO	6	0.0	40.0	18.3	15.7	22.5
	Philippines	PHL	72	0.0	62.5	20.4	19.0	17.7
	Malaysia	MYS	37	0.0	55.3	20.8	16.4	17.3
	Cambodia	KHM	17	0.0	33.0	12.1	9.1	13.6
	Nepal	NPL	10	0.2	39.0	16.2	16.1	13.3
	Thailand	THA	44	0.0	49.0	15.7	15.7	10.3
	Vietnam	VNM	28	0.0	40.6	10.0	10.7	6.3
	Lao People's Democratic Republic	LAO	17	0.0	31.6	8.5	9.0	4.5
	India	IND	276	0.0	60.8	8.1	12.3	1.9
	Myanmar	MMR	61	0.0	29.6	4.2	5.7	1.7
	Indonesia	IDN	316	0.0	50.3	6.2	10.0	1.3
	Bangladesh	BGD	12	0.0	39.7	7.0	13.3	0.8
	Pakistan	PAK	69	0.0	43.9	2.8	7.0	0.3
	Maldives	MDV	15	0.0	44.4	7.7	16.3	0.0
	Spratly Islands	PG	4	0.0	0.0	0.0	0.0	0.0
Temperate Asia	Korea, Republic of	KOR	13	16.7	71.3	46.1	17.3	46.4
	Taiwan	TWN	8	0.0	79.2	42.3	36.9	53.3
	Japan	JPN	78	0.0	70.3	22.7	19.5	16.2
	Korea, Democratic People's Republic of	PRK	11	0.0	64.1	8.1	18.7	1.9
	Kyrgyzstan	KGZ	12	0.0	3.4	1.3	1.0	1.2
	Tajikistan	TJK	12	0.0	3.1	1.1	1.0	1.0
	Mongolia	MNG	124	0.0	31.8	4.5	7.3	0.7
	China	CHN	774	0.0	44.2	2.8	6.1	0.2
	Kazakhstan	KAZ	224	0.0	44.3	3.0	8.6	0.0
	Russia	RUS	1,456	0.0	81.1	2.0	7.1	0.0
	Turkmenistan	TKM	38	0.0	4.8	0.6	1.2	0.0
	Uzbekistan	UZB	37	0.0	14.9	0.9	2.6	0.0
Greater Middle East	Israel	ISR	3	71.4	80.7	76.8	4.9	78.5
	United Arab Emirates	ARE	7	18.1	72.2	61.4	19.2	68.3
	Qatar	QAT	1	40.2	40.2	40.2	-	40.2
	Kuwait	KWT	1	36.3	36.3	36.3	-	36.3
	Morocco	MAR	34	4.5	48.4	23.7	13.4	25.5
	Tunisia	TUN	17	1.5	39.9	17.1	12.5	15.7
	Jordan	JOR	8	0.0	71.1	26.5	31.9	9.9
	Turkey	TUR	67	0.0	54.9	11.9	12.9	7.2
	Armenia	ARM	2	2.0	12.0	7.0	7.1	7.0
	Georgia	GEO	8	1.8	12.6	6.5	3.6	6.0
	Syrian Arab Republic	SYR	17	0.0	30.5	7.3	9.2	5.3
	Egypt	EGY	81	0.0	71.0	10.9	14.9	5.0
	Oman	OMN	27	0.0	52.4	9.5	14.0	3.8
	Iran, Islamic Republic of	IRN	137	0.0	27.3	3.5	5.1	1.5
	Afghanistan	AFG	51	0.0	18.3	3.1	4.3	0.9
	Azerbaijan	AZE	7	0.0	4.6	1.2	1.6	0.9
	Iraq	IRQ	35	0.0	30.4	4.3	8.2	0.9
	Algeria	DZA	194	0.0	25.0	1.8	3.8	0.0
	Libya	LBY	133	0.0	9.1	0.5	1.6	0.0
	Saudi Arabia	SAU	163	0.0	60.4	1.3	5.7	0.0
	Yemen	YEM	38	0.0	8.2	0.9	2.0	0.0
	Western Sahara	ESH	25	0.0	24.6	1.4	4.9	0.0
Sub-Saharan Africa	St. Helena	SHN	4	0.0	100.0	62.5	47.9	75.0
	Swaziland	SWZ	1	64.5	64.5	64.5	-	64.5
	South Africa	ZAF	104	2.1	100.0	56.7	16.7	61.5
	Sao Tome and Principe	STP	2	44.4	60.9	52.7	11.6	52.7
	Reunion	REU	1	52.2	52.2	52.2	-	52.2
	Lesotho	LSO	3	49.4	54.8	51.6	2.8	50.6
	Rwanda	RWA	2	38.1	52.9	45.5	10.5	45.5
	Mauritius	MUS	2	16.7	73.1	44.9	39.9	44.9
	Cape Verde	CPV	8	0.0	65.7	30.8	26.8	31.5
	Burundi	BDI	3	5.0	50.0	28.2	22.6	29.6

								**Variation among countries (continued)**
**GeoRegion**	**Country**	**Code**	***N* cells**	**Min**	**Max**	**Mean**	**SD**	**Median**
	Malawi	MWI	11	0.9	34.6	20.5	12.6	26.9
	Uganda	UGA	19	3.2	60.9	24.9	17.6	20.2
	Zimbabwe	ZWE	32	0.5	55.0	19.3	15.6	15.7
	Comoros	COM	2	11.5	19.6	15.6	5.8	15.6
	Namibia	NAM	66	0.0	63.1	20.2	16.4	15.6
	Botswana	BWA	46	0.0	61.6	20.8	18.1	13.8
	Liberia	LBR	8	0.7	47.5	20.1	17.0	13.7
	Equatorial Guinea	GNQ	4	2.2	37.5	16.7	15.5	13.6
	Ghana	GHA	21	0.8	40.7	15.0	13.4	12.0
	Madagascar	MDG	54	0.0	69.9	17.2	18.9	11.0
	Senegal	SEN	18	0.2	50.6	14.9	14.6	10.1
	Sierra Leone	SLE	6	4.9	30.4	13.5	10.2	9.5
	Kenya	KEN	48	0.0	69.6	19.4	21.3	9.1
	Benin	BEN	11	2.1	18.3	7.8	5.1	6.1
	Tanzania, United Republic of	TZA	76	0.0	54.5	12.6	15.5	5.9
	Guinea-Bissau	GNB	2	0.5	9.9	5.2	6.6	5.2
	Ivory Coast	CIV	27	0.0	36.7	5.8	7.1	4.9
	Gabon	GAB	21	0.0	29.0	7.2	8.6	4.7
	Togo	TGO	5	0.8	9.8	4.8	3.4	4.7
	Burkina Faso	BFA	22	0.0	13.7	4.9	4.5	3.6
	Cameroon	CMR	41	0.0	38.5	9.1	11.0	3.3
	Mayotte	MYT	1	2.3	2.3	2.3	-	2.3
	Zambia	ZMB	57	0.0	49.2	9.2	13.4	1.8
	Congo, Democratic Republic of	COD	194	0.0	67.7	6.7	11.6	1.7
	Mozambique	MOZ	67	0.0	70.1	5.6	12.9	1.7
	Guinea	GIN	22	0.0	44.4	8.1	13.6	0.9
	Congo, Republic of	COG	27	0.0	21.9	2.5	5.0	0.8
	Ethiopia	ETH	93	0.0	36.9	4.1	7.8	0.8
	Angola	AGO	101	0.0	60.7	3.3	7.7	0.7
	Eritrea	ERI	9	0.0	19.2	3.8	7.2	0.3
	Nigeria	NGA	72	0.0	26.0	1.7	4.5	0.3
	Central African Republic	CAF	51	0.0	22.7	0.6	3.2	0.0
	Chad	TCD	103	0.0	11.2	0.5	1.8	0.0
	Djibouti	DJI	3	0.0	0.3	0.1	0.1	0.0
	Mali	MLI	101	0.0	6.7	0.4	0.9	0.0
	Mauritania	MRT	81	0.0	7.6	0.5	1.1	0.0
	Niger	NER	98	0.0	10.7	0.7	1.9	0.0
	Seychelles	SYC	11	0.0	79.5	11.9	24.2	0.0
	Somalia	SOM	57	0.0	15.6	1.3	3.0	0.0
	Sudan	SDN	204	0.0	37.4	1.3	3.9	0.0

Supplementary Table 2: Variation in 110 km inventory completeness (%) for all three vertebrate groups combined (N = 21,170 species) among **a)** biomes, **b)** realms, **c)** biome-realm-combinations (following[94]), and **d)** countries. Within biomes, realms are ordered from highest to lowest median completeness. Within broad geographical regions, countries are ordered from highest to lowest median completeness. Grouping of countries into geographical regions is for orientation only and does not reflect any view of the authors. Some countries are missing because they did not overlay the majority of the land area of any grid cell. Country codes (ISO 3166 standard) are the same as in Fig. 5.

Supplementary Table 3

Country	Non-inventoried spp-cell	% of non-inventoried spp-cell
Brazil	451,427	15.4
Russia	260,523	8.9
China	201,422	6.9
India	106,128	3.6
Indonesia	103,898	3.6
Congo, Democratic Republic of	98,291	3.4
Canada	74,129	2.5
Sudan	61,617	2.1
Colombia	61,122	2.1
USA	58,822	2.0
Peru	57,550	2.0
Argentina	51,619	1.8
Venezuela	50,096	1.7
Angola	47,694	1.6
Kazakhstan	45,568	1.6
Ethiopia	43,609	1.5
Tanzania	43,367	1.5
Bolivia	42,583	1.5
Australia	40,854	1.4
Myanmar	38,141	1.3
Nigeria	37,055	1.3
Zambia	34,246	1.2
Mozambique	34,066	1.2
Mexico	32,127	1.1
Iran, Islamic Republic of	27,411	0.9
Mali	24,308	0.8
Central African Republic	24,096	0.8
Kenya	23,930	0.8
Mongolia	23,835	0.8
Chad	23,608	0.8
Thailand	23,422	0.8
Cameroon	23,281	0.8
South Africa	19,359	0.7
Papua New Guinea	18,648	0.6
Malaysia	18,515	0.6
Niger	17,865	0.6
Namibia	17,842	0.6
Pakistan	17,135	0.6
Philippines	16,439	0.6
Zimbabwe	16,418	0.6
Turkey	16,375	0.6
Algeria	16,365	0.6
Vietnam	16,066	0.5
Somalia	15,873	0.5
Côte d'Ivoire	15,016	0.5
Botswana	14,617	0.5
Saudi Arabia	14,505	0.5
Congo, Republic of	13,677	0.5
Guyana	13,499	0.5
Paraguay	13,415	0.5

Supplementary Table 3: Top 50 countries based on number of species-grid cell combinations that are missing from country-wide completeness of 100% at the 110 km grain (‘Non-inventoried species spp-cell’). Countries are ordered from highest to lowest percentage of non-inventoried species presences (‘% of non-inventoried spp-cell).

Supplementary Table 4

Taxonomic group	*N* records	Linkable to DB	Not accepted name	Ambiguous name	Validated records
Birds	177,067,882 (100%)	176,698,744 (99.8%)	16,830,672 (9.5%)	26,210,816 (14.8%)	152,429,094 (86.1%)
Mammals	4,725,561 (100.0%)	4,708,363 (99.6%)	625,540 (13.2%)	308,662 (6.5%)	3,355,082 (71.0%)
Amphibians	1,695,155 (100.0%)	1,689,766 (99.7%)	416,666 (24.6%)	642,943 (37.9%)	1,302,072 (76.8%)
Total	183,488,598 (100%)	183,096,873 (99.8%)	17,872,878 (9.7%)	27,162,421 (14.8%)	157,086,248 (85.6%)

Supplementary Table 4: Results of the geographic and taxonomic validation of records. Of the geo-referenced specimen and observation data with a binomial or trinomial scientific names that passed initial filtering (see '*N* records'), between 99.6 and 99.8% could be linked to our taxonomic database (see 'Linkable to DB'). Between 9.5 and 24.6% of records are stored under a name that is not an accepted species name according to our three "master" taxonomies, e.g., a synonym or subspecies name, and thus required taxonomic name standardization (see 'Not accepted name'). 6.5 to 37.9% of records had ambiguous names, i.e., accepted names or synonyms that could refer to more than one accepted species, and thus required combined taxonomic and geographic inference to determine the most parsimonious species identity (see 'Ambiguous name'). 71.0 to 86.1% of records remained after taxonomic and geographic validation, i.e., the record could be confidently assigned to one accepted species, and was also collected within the presumed current distribution of that species (see 'Validated records').

Supplementary Table 5

Taxon	Region	Total cells	Cells included	% cells included
**Birds**	South America	1,506	876	58.17
	Central America/Caribbean	332	218	65.66
	Northern America	1,655	1,555	93.96
	North/West Europe	431	352	81.67
	East/South-East Europe	198	185	93.43
	Australia/Oceania	1,031	680	65.96
	Tropical Asia	913	481	52.68
	Temperate Asia	2,770	777	28.05
	Greater Middle East	1,157	317	27.40
	Sub-Saharan Africa	2,018	700	34.69
**Mammals**	South America	1,493	792	53.05
	Central America/Caribbean	320	218	68.12
	Northern America	1,640	1,173	71.52
	North/West Europe	424	335	79.01
	East/South-East Europe	198	121	61.11
	Australia/Oceania	859	621	72.29
	Tropical Asia	896	394	43.97
	Temperate Asia	2,760	490	17.75
	Greater Middle East	1,157	279	24.11
	Sub-Saharan Africa	2,005	591	29.48
**Amphibians**	South America	1,467	636	43.35
	Central America/Caribbean	318	197	61.95
	Northern America	1,376	1,012	73.55
	North/West Europe	345	315	91.30
	East/South-East Europe	198	89	44.95
	Australia/Oceania	739	527	71.31
	Tropical Asia	849	199	23.44
	Temperate Asia	2,275	374	16.44
	Greater Middle East	726	147	20.25
	Sub-Saharan Africa	1,903	374	19.65

Supplementary Table 5: Grid cells selected for models of point record density and inventory completeness in different regions. Shown are total numbers of cells inhabited by the vertebrate group (Total cells), total numbers (Cells included) and percentages (% cells included) of cells that were included in models.

Supplementary Table 6

a) Inventory completeness.

Taxon	Grain (km)	D^2	Moran's I_{nsp}	Moran's I_{sp}
Birds	110	0.78	0.067***	0.007***
	220	0.76	0.057***	0.003***
	440	0.77	0.040***	-0.003
	880	0.74	0.012	-0.012
Mammals	110	0.70	0.081***	0.006***
	220	0.75	0.079***	0.006***
	440	0.77	0.061***	-0.003
	880	0.73	0.030***	-0.006
Amphibians	110	0.57	0.062***	0.008***
	220	0.64	0.066***	0.008***
	440	0.60	0.064***	0.001
	880	0.60	0.059***	-0.005

b) Record density.

Taxon	Grain (km)	R^2_{sp}	R^2_{nsp}	Moran's I_{nsp}	Moran's I_{sp}
Birds	110	0.82	0.62	0.086***	0.006***
	220	0.83	0.70	0.069***	0.006***
	440	0.85	0.78	0.047***	0.007**
	880	0.86	0.82	0.025***	0.005
Mammals	110	0.66	0.41	0.068***	0.005***
	220	0.76	0.53	0.070***	0.007***
	440	0.80	0.59	0.060***	0.004.
	880	0.76	0.71	0.030***	0.006
Amphibians	110	0.58	0.38	0.063***	0.006***
	220	0.69	0.53	0.062***	0.005***
	440	0.77	0.59	0.060***	0.002
	880	0.83	0.70	0.046***	-0.000

Supplementary Table 6: Model fits and spatial autocorrelation for **a)** inventory completeness (RAC models) and **b)** record density (SAR models). Values are given for the model subset with the lowest AIC score. In a) model fit is expressed by the deviance explained (D^2). The degree of spatial autocorrelation (global Moran's I) in model residuals is compared between the minimum adequate spatial model subset (see 'Moran's I_{sp}') and the corresponding non-spatial model (see 'Moran's I_{nsp}'). Asterisks denote significant spatial autocorrelation (.: *P*<0.1; *: *P*<0.05; **: *P*<0.01; ***: *P*<0.001). In b) model fit is expressed by pseudo-R^2 values, calculated as the squared Pearson correlation coefficient between fitted and observed values [82]. Fitted values of SAR models can be partitioned additively into trend (non-spatial smooth) and signal (spatial smooth). We calculated both a pseudo-R^2 for the fitted values including the spatial component ('R^2sp'), and a pseudo-R^2 for the trend excluding the spatial component, which represents the part of the variation explained by the predictors (in the context of SAR models hereafter 'R^2_{nsp}'). R^2 values of potential minimum adequate models (subsets with ΔAIC < 2) never differed by more than 0.004. The degree of spatial autocorrelation (global Moran's I) in model residuals is compared between the minimum adequate spatial model (see 'Moran's I_{sp}') and the corresponding non-spatial (OLS) model (see 'Moran's I_{nsp}'). Asterisks denote significant spatial autocorrelation (.: *P*<0.1; *: *P*<0.05; **: *P*<0.01; ***: *P*<0.001).

Supplementary Table 7

a) Adding country identity to MAM.

Taxon	Grain (km)	D^2_{MAM}	$D^2_{MAM+Country}$	$D^2_{Country}$	% of cross-country variation already in D^2_{MAM}	% of D^2 added by Country
Birds	110	0.78	0.80	0.68	97.2	2.4
Mammals	110	0.70	0.73	0.64	94.7	4.6
Amphibians	110	0.57	0.62	0.55	92.1	7.1

b) Adding record density to MAM.

Taxon	Grain (km)	D^2_{MAM}	D^2_{MAM+RD}	D^2_{RD}	% of D^2_{MAM} in ΔRD	% of D^2 added by RD
Birds	110	0.78	0.94	0.90	94.2	5.8
	220	0.76	0.94	0.89	94.3	5.7
	440	0.77	0.92	0.88	95.2	4.8
	880	0.74	0.86	0.82	95.2	4.8
Mammals	110	0.70	0.88	0.76	83.7	16.3
	220	0.75	0.89	0.79	86.9	13.1
	440	0.77	0.89	0.81	89.0	11.0
	880	0.73	0.87	0.79	89.8	10.2
Amphibians	110	0.57	0.76	0.59	69.1	30.9
	220	0.64	0.79	0.64	76.8	23.2
	440	0.60	0.80	0.63	72.3	27.7
	880	0.60	0.76	0.57	68.0	32.0

Supplementary Table 7: Influence of adding a) country identity of grid cells as a factor and b) record density to the minimum adequate model of inventory completeness. D^2_{MAM} is the deviance explained by the minimum adequate model. In a): $D^2_{MAM+Country}$ is the deviance explained when adding a country factor to the minimum adequate model. $D^2_{Country}$ is the deviance explained by a model containing only country membership as factor. The percentage of cross-country variation that is already captured by the minimum adequate model (% of cross-country variation already in D^2_{MAM}) was calculated as: 100 / $D^2_{Country}$*($D^2_{Country}$ - ($D^2_{MAM+Country}$ - D^2_{MAM})). %D^2 added by Country is the additional deviance explained by adding a country factor to the minimum adequate model (as percent of total D^2); in b): D^2_{MAM+RD} is the deviance explained when adding log_{10}-transformed record density to the minimum adequate model. D^2_{RD} is the deviance explained by a model containing only log_{10}-transformed record density as an explanatory variable. The percentage of the deviance explained by the MAM that is also attributable to differences in record density (% of D^2_{MAM} in ΔRD) was calculated as: 100 / D^2_{MAM}* (D^2_{MAM} - (D^2_{RD} - D^2_{MAM+RD})). %D^2 added by RD is the additional deviance explained by adding record density to the minimum adequate model (as percent of total D^2_{MAM+RD}).

Supplementary Table 8

a) Inventory completeness at 110 km.					
**Taxonomic group**	**Predictor**	**d²**	**GLM β (range)**	**z-value**	**% SS**
Birds	EndRich	0.01***	0.32***	127.21	0.01
	ProtAreas	0.03***	0.19***	80.96	0.01
	Mountains	0.00***	-0.03***	-11.83	0.00
	GroundAcc	0.03***	0.23***	72.45	0.00
	ProxAirp	0.16***	0.18***	57.32	0.03
	ProxInst	0.29***	0.35***	121.61	0.15
	Security	0.12***	0.08***	27.93	0.01
	GBIFpartic	0.27***	0.38***	134.25	0.13
	ScientActiv	0.39***	0.27***	56.93	0.22
	FundLocal	0.34***	0.61***	126.60	0.21
	FundInst	0.01***	-0.13***	-59.17	0.00
	PublSize	0.18***	0.53***	173.70	0.22
Mammals	EndRich	0.00	0.25***	47.92	0.01
	ProtAreas	0.02***	0.13***	26.50	0.00
	Mountains	0.01***	0.07***	14.28	0.00
	GroundAcc	0.05***	0.02*	2.25	0.00
	ProxAirp	0.12***	0.07***	10.51	0.00
	ProxInst	0.40***	0.61***	87.06	0.72
	Security	0.07***	-0.04***	-6.34	0.00
	GBIFpartic	0.25***	0.26***	38.41	0.10
	ScientActiv	0.27***	-0.01	-0.80	0.06
	FundLocal	0.24***	0.30***	29.94	0.08
	FundInst	0.02***	-0.06***	-12.24	0.01
	PublSize	0.02***	0.15***	25.34	0.01
Amphibians	EndRich	0.00***	0.08***	10.93	0.00
	ProtAreas	0.01***	0.12***	14.35	0.01
	Mountains	0.01***	0.13***	15.32	0.04
	GroundAcc	0.06***	0.12***	11.71	0.01
	ProxAirp	0.11***	0.05***	5.11	0.02
	ProxInst	0.25***	0.30***	29.13	0.56
	Security	0.07***	-0.16***	-16.03	0.03
	GBIFpartic	0.19***	0.24***	28.50	0.26
	ScientActiv	0.16***	-0.02		0.04
	FundLocal	0.13***	0.17*** (0.17 - 0.18)	14.26	0.03
	FundInst	0.01***	-0.07***	-8.40	0.01
	PublSize	0.00***	0.02	1.63	0.00

b) Inventory completeess at 220 km.					
**Taxonomic group**	**Predictor**	**d²**	**GLM β (range)**	**z-value**	**% SS**
Birds	EndRich	0.00***	0.32***	76.97	0.01
	ProtAreas	0.04***	0.20***	50.28	0.01
	Mountains	0.01***	0.06***	16.83	0.00
	GroundAcc	0.02***	0.10***	21.44	0.00
	ProxAirp	0.21***	0.30***	59.08	0.07
	ProxInst	0.30***	0.42***	95.26	0.25
	Security	0.15***	-0.16***	-31.89	0.02
	GBIFpartic	0.29***	0.38***	89.00	0.16
	ScientActiv	0.32***	0.10***	13.75	0.11
	FundLocal	0.23***	0.56***	84.87	0.11
	FundInst	0.02***	-0.15***	-43.89	0.01
	PublSize	0.19***	0.53***	108.44	0.24
Mammals	EndRich	0.01***	0.38***	45.10	0.02
	ProtAreas	0.02***	0.12***	16.44	0.00
	Mountains	0.02***	0.03***	4.03	0.00
	GroundAcc	0.04***	-0.01	-1.05	0.00
	ProxAirp	0.16***	0.16***	16.52	0.02
	ProxInst	0.41***	0.61***	61.87	0.65
	Security	0.07***	-0.03***	-3.41	0.00
	GBIFpartic	0.31***	0.33***	34.11	0.20
	ScientActiv	0.26***	-0.01	-0.71	0.05

Inventory completeess at 220 km (continued)

Taxonomic group	Predictor	d²	GLM β (range)	z-value	% SS
	FundLocal	0.18***	0.32***	24.10	0.04
	FundInst	0.03***	-0.06***	-8.16	0.01
	PublSize	0.02***	0.17***	17.41	0.01
Amphibians	EndRich	0.00.	0.12***	11.04	0.00
	ProtAreas	0.00***	0.10***	8.10	0.00
	Mountains	0.03***	0.11***	8.77	0.03
	GroundAcc	0.09***	0.12***	8.79	0.04
	ProxAirp	0.18***	0.14***	9.36	0.08
	ProxInst	0.26***	0.24***	15.90	0.34
	Security	0.06***	-0.12***	-8.01	0.01
	GBIFpartic	0.24***	0.24***	16.93	0.41
	ScientActiv	0.19***	0.09***	3.58	0.07
	FundLocal	0.12***	0.18***	8.22	0.03
	FundInst	0.02***	-0.13***	-9.76	0.00
	PublSize	0.01***	0.04*	2.44	0.00

c) Inventory completeness at 440 km.

Taxonomic group	Predictor	d²	GLM β (range)	z-value	% SS
Birds	EndRich	0.03***	0.41***	53.94	0.04
	ProtAreas	0.08***	0.27*** (0.27 - 0.28)	36.99	0.06
	Mountains	0.01***	0.05***	9.14	0.00
	GroundAcc	0.02***	-0.02*	-2.53	0.00
	ProxAirp	0.23***	0.29*** (0.28 - 0.29)	32.86	0.11
	ProxInst	0.30***	0.34***	46.12	0.31
	Security	0.17***	-0.12***	-14.12	0.02
	GBIFpartic	0.28***	0.36***	52.56	0.20
	ScientActiv	0.21***	-0.09***	-9.68	0.03
	FundLocal	0.15***	0.65***	63.13	0.10
	FundInst	0.05***	-0.20***	-31.61	0.02
	PublSize	0.16***	0.45***	53.53	0.11
Mammals	EndRich	0.02***	0.45***	30.81	0.04
	ProtAreas	0.05***	0.22***	16.70	0.04
	Mountains	0.02***	0.08***	7.28	0.01
	GroundAcc	0.03***	-0.03*	-2.10	0.00
	ProxAirp	0.17***	0.13***	8.87	0.03
	ProxInst	0.37***	0.49***	32.15	0.47
	Security	0.07***	0.00	0.24	0.00
	GBIFpartic	0.33***	0.40***	28.94	0.34
	ScientActiv	0.17***	-0.11***	-6.08	0.02
	FundLocal	0.10***	0.43***	22.43	0.03
	FundInst	0.10***	-0.15***	-11.96	0.03
	PublSize	0.00***	0.07***	5.01	0.00
Amphibians	EndRich	0.00	0.14***	8.48	0.00
	ProtAreas	0.00.	0.09***	4.75	0.00
	Mountains	0.02***	0.08***	4.38	0.01
	GroundAcc	0.07***	0.17***	9.15	0.05
	ProxAirp	0.14***	0.17***	8.27	0.08
	ProxInst	0.24***	0.12***	5.98	0.25
	Security	0.05***	-0.18***	-8.22	0.02
	GBIFpartic	0.20***	0.27***	14.35	0.46
	ScientActiv	0.14***	0.01	0.42	0.04
	FundLocal	0.12***	0.30***	11.11	0.09
	FundInst	0.04***	-0.15*** (-0.16 - .0.15)	-8.89	0.01
	PublSize	0.01***	0.01		0.00

d) Inventory completeness at 880 km.

Taxonomic group	Predictor	d²	GLM β (range)	z-value	% SS
Birds	EndRich	0.07***	0.41***	31.67	0.08
	ProtAreas	0.08***	0.21***	14.05	0.03
	Mountains	0.02***	-0.04***	-3.67	0.00
	GroundAcc	0.02***	-0.03*	-2.23	0.00

Inventory completeness at 880 km (continued)

Taxonomic group	Predictor	d²	GLM β (range)	z-value	% SS
	ProxAirp	0.19***	0.32***	25.56	0.11
	ProxInst	0.28***	0.33*** (0.33 - 0.34)	24.05	0.34
	Security	0.20***	-0.12***	-8.07	0.02
	GBIFpartic	0.30***	0.38***	32.61	0.23
	ScientActiv	0.18***			0.02
	FundLocal	0.10***	0.49*** (0.48 - 0.49)	33.06	0.09
	FundInst	0.05***	-0.03*	-2.40	0.01
	PublSize	0.13***	0.29*** (0.28 - 0.29)	19.48	0.06
Mammals	EndRich	0.04***	0.29***	12.57	0.04
	ProtAreas	0.07***	0.29***	12.27	0.06
	Mountains	0.03***	0.12***	5.90	0.01
	GroundAcc	0.02***	0.06**	2.77	0.00
	ProxAirp	0.12***	0.18***	9.86	0.03
	ProxInst	0.39***	0.36***	17.67	0.42
	Security	0.11***	-0.07**	-2.96	0.00
	GBIFpartic	0.36***	0.38***	19.30	0.32
	ScientActiv	0.21***			0.03
	FundLocal	0.09***	0.31***	13.23	0.05
	FundInst	0.11***			0.03
	PublSize	0.01***	-0.02	-0.83	0.00
Amphibians	EndRich	0.00			0.00
	ProtAreas	0.02***	-0.11***	-3.80	0.00
	Mountains	0.00**	0.01		0.00
	GroundAcc	0.07***	0.23***	8.86	0.09
	ProxAirp	0.11***	0.26***	12.07	0.13
	ProxInst	0.17***	0.24***	9.84	0.31
	Security	0.09***	-0.44***	-14.46	0.09
	GBIFpartic	0.13***	0.32***	12.66	0.23
	ScientActiv	0.19***			0.08
	FundLocal	0.13***			0.06
	FundInst	0.00			0.00
	PublSize	0.00	0.27*** (0.26 - 0.27)	8.76	0.00

e) Record density at 110 km.

		r²	SAR β (range)	z-value	% SS
Birds	EndRich	0.01***	0.28***	14.66	0.07
	ProtAreas	0.04***	0.06***	7.47	0.00
	Mountains	0.00	0.03*	2.15	0.00
	GroundAcc	0.06***	0.16***	10.35	0.05
	ProxAirp	0.23***	0.15***	11.81	0.00
	ProxInst	0.28***	0.11*** (0.11 - 0.12)	5.11	0.04
	Security	0.09***	-0.04.	-1.93	0.00
	GBIFpartic	0.29***	0.21*** (0.21 - 0.22)	8.65	0.14
	ScientActiv	0.33***	-0.05 (-0.06 - -0.05)	-1.55	0.20
	FundLocal	0.25***	0.38*** (0.38 - 0.39)	10.73	0.24
	FundInst	0.01***	-0.03**	-3.05	0.01
	PublSize	0.21***	0.17***	22.82	0.24
Mammals	EndRich	0.03***	0.30***	17.79	0.19
	ProtAreas	0.03***	0.04***	4.13	0.01
	Mountains	0.01***	0.08***	4.78	0.01
	GroundAcc	0.03***	0.09***	4.38	0.01
	ProxAirp	0.12***	0.10***	5.29	0.01
	ProxInst	0.24***	0.29***	8.72	0.22
	Security	0.03***	0.06*	2.29	0.00
	GBIFpartic	0.18***	0.20***	6.66	0.18
	ScientActiv	0.17***	-0.05	-1.06	0.19
	FundLocal	0.09***	0.24***	4.99	0.06
	FundInst	0.02***	-0.04**	-3.00	0.03
	PublSize	0.04***	0.11***	11.10	0.09
Amphibians	EndRich	0.08***	0.34***	18.07	0.55
	ProtAreas	0.02***	0.07***	5.12	0.00
	Mountains	0.00***	0.05** (0.05 - 0.06)	2.62	0.00

Taxonomic group	Predictor	d²	GLM β (range)	z-value	% SS
	GroundAcc	0.08***	0.11***	4.78	0.02
	ProxAirp	0.14***	0.11***	5.33	0.03
	ProxInst	0.14***	0.33*** (0.32 - 0.33)	9.99	0.26
	Security	0.01***			0.00
	GBIFpartic	0.05***	0.03 (0.03 - 0.04)	1.0	0.01
	ScientActiv	0.07***	0.12*** (0.09 - 0.12)	3.18	0.06
	FundLocal	0.03***	0.05	0.86	0.02
	FundInst	0.00.	-0.09*** (-0.10 - -0.09)	-5.53	0.00
	PublSize	0.04***	0.14***	9.26	0.06

f) Record density at 220 km.

Taxonomic group	Predictor	r²	SAR β (range)	z-value	% SS
Birds	EndRich	0.02***	0.33*** (0.33 - 0.35)	10.83	0.09
	ProtAreas	0.06***	0.07***	5.62	0.00
	Mountains	0.00	0.03*	1.97	0.00
	GroundAcc	0.06***	0.13***	5.41	0.02
	ProxAirp	0.29***	0.13***	7.26	0.03
	ProxInst	0.34***	0.17***	6.34	0.02
	Security	0.16***	-0.07*	-2.51	0.00
	GBIFpartic	0.38***	0.22***	7.37	0.24
	ScientActiv	0.36***	-0.04	-1.07	0.20
	FundLocal	0.25***	0.34***	8.24	0.25
	FundInst	0.04***	-0.06***	-3.90	0.03
	PublSize	0.18***	0.13***	10.88	0.19
Mammals	EndRich	0.07***	0.40*** (0.40 - 0.42)	14.81	0.35
	ProtAreas	0.05***	0.06***	3.80	0.01
	Mountains	0.01***	0.04.	1.82	0.00
	GroundAcc	0.05***	0.08**	2.82	0.00
	ProxAirp	0.16***	0.07**	3.02	0.01
	ProxInst	0.29***	0.31*** (0.30 - 0.31)	8.24	0.17
	Security	0.05***	0.04	1.13	0.00
	GBIFpartic	0.24***	0.21***	5.45	0.20
	ScientActiv	0.19***	-0.00	-0.05	0.16
	FundLocal	0.07***	0.24***	4.65	0.04
	FundInst	0.04***	-0.05**	-3.09	0.05
	PublSize	0.02***	0.12***	9.04	0.03
Amphibians	EndRich	0.14***	0.41*** (0.41 - 0.42)	15.94	0.59
	ProtAreas	0.01***	0.07*** (0.06 - 0.07)	3.45	0.00
	Mountains	0.01**	-0.01	-0.24	0.00
	GroundAcc	0.09***	0.11*** (0.11 - 0.12)	3.54	0.01
	ProxAirp	0.20***	0.10***	4.03	0.08
	ProxInst	0.22***	0.34*** (0.33 - 0.35)	9.03	0.23
	Security	0.02***	0.00		0.00
	GBIFpartic	0.11***	0.07.	1.65	0.02
	ScientActiv	0.13***	0.16*** (0.12 - 0.20)	3.61	0.04
	FundLocal	0.05***	0.07	1.25	0.01
	FundInst	0.00	-0.11***	-4.62	0.00
	PublSize	0.04***	0.14*** (0.13 - 0.14)	6.18	0.02

g) Record density at 440 km.

Taxonomic group	Predictor	r²	SAR β (range)	z-value	% SS
Birds	EndRich	0.04***	0.37*** (0.37 - 0.38)	8.65	0.13
	ProtAreas	0.09***	0.10*** (0.09 - 0.10)	4.49	0.01
	Mountains	0.00	-0.00		0.00
	GroundAcc	0.08***	0.09* (0.07 - 0.09)	2.48	0.00
	ProxAirp	0.30***	0.10*** (0.10 - 0.11)	3.61	0.01
	ProxInst	0.37***	0.15*** (0.13 - 0.15)	4.39	0.26
	Security	0.22***	-0.04 (-0.06 - -0.04)	-1.24	0.00
	GBIFpartic	0.45***	0.26*** (0.24 - 0.27)	7.64	0.26
	ScientActiv	0.34***	-0.02	-0.34	0.08
	FundLocal	0.23***	0.43*** (0.40 - 0.44)	10.36	0.20
	FundInst	0.06***	-0.08** (-0.09 - -0.08)	-2.66	0.01
	PublSize	0.13***	0.09*** (0.08 - 0.09)	4.34	0.03

Record density at 440 km (continued)

Taxonomic group	Predictor	d²	GLM β (range)	z-value	% SS
Mammals	EndRich	0.11***	0.47***(0.47 - 0.48)	10.43	0.37
	ProtAreas	0.09***	0.06* (0.06 - 0.07)	2.51	0.04
	Mountains	0.01**	0.01	-	0.00
	GroundAcc	0.06***	0.04 (0.04 - 0.06)	0.95	0.00
	ProxAirp	0.17***	0.05 (0.04 - 0.05)	1.58	0.00
	ProxInst	0.30***	0.25*** (0.24 - 0.25)	5.44	0.24
	Security	0.06***	0.04 (0.03 - 0.04)	0.92	0.00
	GBIFpartic	0.30***	0.29***	4.34	0.21
	ScientActiv	0.16***	0.04 (0.04 - 0.05)	0.64	0.04
	FundLocal	0.04***	0.23*** (0.21 - 0.24)	4.03	0.02
	FundInst	0.11***	-0.13***	-4.79	0.08
	PublSize	0.00	0.09***	3.99	0.00
Amphibians	EndRich	0.15***	0.45*** (0.45 - 0.46)	11.96	0.55
	ProtAreas	0.01*	0.03	1.08	0.00
	Mountains	0.00	-0.03	-0.84	0.00
	GroundAcc	0.12***	0.13** (0.13 - 0.14)	3.08	0.01
	ProxAirp	0.16***	0.06* (0.06 - 0.07)	2.06	0.01
	ProxInst	0.27***	0.22*** (0.21 - 0.22)	4.99	0.37
	Security	0.07***	-0.03	-0.74	0.00
	GBIFpartic	0.16***	0.05	0.98	0.01
	ScientActiv	0.17***	0.16** (0.13 - 0.16)	2.72	0.03
	FundLocal	0.08***	0.21***	3.35	0.03
	FundInst	0.00	-0.10*** (-0.11 - -0.10)	-3.26	0.00
	PublSize	0.03***	0.07* (0.07 - 0.08)	2.47	0.00

h) Record density at 880 km.

Taxonomic group	Predictor	r²	SAR β (range)	z-value	% SS
Birds	EndRich	0.02.	0.31*** (0.30 - 0.33)	5.37	0.11
	ProtAreas	0.08***	0.15*** (0.15 - 0.16)	4.01	0.01
	Mountains	0.00	0.03 (0.02 - 0.03)	0.93	0.00
	GroundAcc	0.09***	0.11* (0.10 - 0.12)	2.38	0.02
	ProxAirp	0.17***	0.11** (0.11 - 0.12)	2.92	0.01
	ProxInst	0.42***	0.25*** (0.24 - 0.26)	5.83	0.33
	Security	0.28***	-0.08. (-0.09 - -0.07)	-1.67	0.02
	GBIFpartic	0.49***	0.20*** (0.20 - 0.28)	3.63	0.25
	ScientActiv	0.38***	0.10. (0.10 - 0.11)	1.82	0.09
	FundLocal	0.20***	0.31*** (0.31 - 0.39)	4.68	0.17
	FundInst	0.06***	-0.02		0.01
	PublSize	0.09***	-0.06 (-0.06 - -0.04)	-1.44	0.01
Mammals	EndRich	0.07***	0.28*** (0.26 - 0.28)	3.89	0.33
	ProtAreas	0.09***	0.19*** (0.17 - 0.19)	3.91	0.03
	Mountains	0.04**	0.13** (0.12 - 0.13)	2.82	0.02
	GroundAcc	0.10***	0.17** (0.13 - 0.18)	2.78	0.04
	ProxAirp	0.11***	0.07 (0.06 - 0.07)	1.34	0.00
	ProxInst	0.34***	0.20** (0.19 - 0.20)	2.94	0.19
	Security	0.11***	0.11. (0.11 - 0.11)	1.84	0.00
	GBIFpartic	0.38***	0.22** (0.16 - 0.22)	2.93	0.16
	ScientActiv	0.24***	0.18* (0.18 - 0.19)	2.41	0.11
	FundLocal	0.04**	0.17* (0.12 - 0.17)	2.15	0.03
	FundInst	0.16***	-0.14** (-0.15 - -0.14)	-2.74	0.10
	PublSize	0.008	0.005		0.00
Amphibians	EndRich	0.20***	0.34*** (0.32 - 0.37)	5.60	0.70
	ProtAreas	0.00	-0.05 (-0.05 - -0.03)	-1.05	0.00
	Mountains	0.00	0.03	0.65	0.00
	GroundAcc	0.14***	0.24*** (0.23 - 0.27)	4.36	0.03
	ProxAirp	0.10***	0.06 (0.06 - 0.07)	1.52	0.01
	ProxInst	0.30***	0.24*** (0.20 - 0.27)	4.62	0.06
	Security	0.16***	-0.16*** (-0.20 - -0.15)	-3.22	0.01
	GBIFpartic	0.19***	0.06 (0.06 - 0.12)	0.88	0.01
	ScientActiv	0.23***	0.15*	2.17	0.09

Record density at 880 km (continued)

Taxonomic group	Predictor	d²	GLM β (range)	z-value	% SS
	FundLocal	0.07***	0.15. (0.15 - 0.22)	1.90	0.08
	FundInst	0.04**	-0.01		0.00
	PublSize	0.03*	0.07. (0.07 - 0.09)	1.77	0.00

Supplementary Table 8: The effects of socioeconomic and geographic factors on a) – d) inventory completeness and e) – h) data density. The twelve predictor variables were endemism richness (EndRich), protected area coverage (ProtAreas), mountains (Mountains), on-ground accessibility (GroundAcc), proximity to airports (ProxAirp), proximity to data-contributing institutions (ProxInst), secure conditions (Security), participation with GBIF (GBIFpartic), scientific activities (ScientActiv), nationally available research funding (FundLocal), research funding in countries with contributing institutions (FundInst), and size of contributing institutions (PublSize). Three comparative measures were used: for inventory completeness (a – d): 1) the deviance explained from simple regressions (d²), 2) standardized regression coefficients from the reduced spatial generalized linear model with the lowest AIC score (GLM β; a range of coefficients is given if several model subsets have ΔAIC < 2 to the "best" model), and 3) the percentage each predictor has in the total Sums of Squares of an ANOVA, where the AIC values of all possible non-spatial models enter as the response variable and dummy-variables coding whether or not a predictor is in the respective model as explanatory variables (% SS); for inventory completeness (e – h): 1) the coefficient of determination from simple ordinary least squares regressions (r²), 2) standardized regression coefficients from the reduced simultaneous autoregressive model with the lowest AIC score (SAR β), and 3) the percentage each predictor has in the total Sums of Squares of an ANOVA, where the AIC values of all possible non-spatial models enter as the response variable and dummy-variables coding whether or not a predictor is in the respective model as explanatory variables (% SS) the. Asterisks denote significant spatial autocorrelation (.: P<0.1; *: P<0.05; **: P<0.01; ***: P<0.001).

Supplementary Table 9

a) Publishers of bird records

Data publisher	Country	Records total / valid (% of total)	Unknown total / valid (% of total)	Observations total / valid (% of total)	Specimens total / valid (% of total)
Avian Knowledge Network	USA	95,339,821<br>84,339,776 (88.5%)	-	95,339,821<br>84,339,776 (88.5%)	-
ArtDatabanken	Sweden	21,040,602<br>17,322,128 (82.3%)	-	21,040,602<br>17,322,128 (82.3%)	-
Birds Australia	Australia	10,969,497<br>9,803,262 (89.4%)	10,969,497<br>9,803,262 (89.4%)	-	-
BirdLife Finland	Finland	7,535,045<br>5,577,806 (74.0%)	55,638<br>35,060 (63.0%)	7,479,407<br>5,542,746 (74.1%)	-
South African National Biodiversity Institute	South Africa	6,792,022<br>6,120,569 (90.1%)	-	6,792,022<br>6,120,569 (90.1%)	-
UK National Biodiversity Network	UK	5,606,751<br>5,058,976 (90.2%)	5,606,751<br>5,058,976 (90.2%)	-	-
Danish Biodiversity Information Facility	Denmark	4,544,665<br>3,595,795 (79.1%)	25,333<br>17,626 (69.6%)	4,509,884<br>3,570,857 (79.2%)	9,448<br>7,312 (77.4%)
GBIF-Sweden	Sweden	4,237,991<br>3,968,443 (93.6%)	-	4,237,809<br>3,968,304 (93.6%)	182<br>139 (76.4%)
The Norwegian Biodiversity Information Centre (NBIC)	Norway	3,827,892<br>3,134,943 (81.9%)	-	3,827,892<br>3,134,943 (81.9%)	-
NSW Dpt. of Environment, Climate Change, and Water	Australia	2,601,841<br>2,109,362 (81.1%)	-	2,601,841<br>2,109,362 (81.1%)	-
Eremaea	Australia	1,207,943<br>1,068,708 (88.5%)	164,041<br>146,662 (89.4%)	1,043,902<br>922,046 (88.3%)	-
Canberra Ornithologists Group	Australia	1,159,524<br>965,904 (83.3%)	-	1,159,524<br>965,904 (83.3%)	-
Service du Patrimoine naturel,Musée national d'Histoire naturelle, Paris	France	960,908<br>909,673 (94.7%)	-	960,908<br>909,673 (94.7%)	-
University of Gdańsk, Bird Migration Research Station	Poland	667,168<br>601,202 (90.1%)	-	667,168<br>601,202 (90.1%)	-
National Biodiversity Data Centre	Ireland	647,220<br>358,159 (55.3%)	-	-	647,220<br>358,159 (55.3%)
Ocean Biogeographic Information System	OBIS	622,491<br>228,500 (36.7%)	976<br>186 (19.1%)	621,515<br>228,314 (36.7%)	-
Dpt. of Natural Resources, Environment (Northern Territory)	Australia	616,706<br>560,637 (90.9%)	-	616,706<br>560,637 (90.9%)	-
Dpt. of Environment and Natural Resources (South Australia)	Australia	586,633<br>527,342 (89.9%)	489<br>481 (98.4%)	585,597<br>526,360 (89.9%)	547<br>501 (91.6%)
Biologiezentrum Linz Oberösterreich	Austria	548,292<br>496,931 (90.6%)	548,292<br>496,931 (90.6%)	-	-
Finnish Museum of Natural History	Finland	513,504<br>340,535 (66.3%)	-	513,504<br>340,535 (66.3%)	-

Supplementary Table 9 (continued)

Data publisher	Country	Records total / valid (% of total)	Unknown total / valid (% of total)	Observations total / valid (% of total)	Specimens total / valid (% of total)
GBIF-Spain	Spain	431,841<br>412,275 (95.5%)	-	429,746<br>410,541 (95.5%)	2,095<br>1,734 (82.8%)
Australian Antarctic Data Centre	Australia	400,449<br>108 (0.0%)	365,283<br>5 (0.0%)	35,166<br>103 (0.3%)	-
Bird Studies Canada	Canada	310,618<br>292,455 (94.2%)	-	310,618<br>292,455 (94.2%)	-
Arctos	USA	249,240<br>218,950 (87.8%)	-	-	249,240<br>218,950 (87.8%)
Yale University Peabody Museum	USA	196,614<br>169,340 (86.1%)	-	-	196,614<br>169,340 (86.1%)
University of Michigan Museum of Zoology	USA	173,337<br>147,644 (85.2%)	-	-	173,337<br>147,644 (85.2%)
KBIF Data Repository	Korea, Republic of	152,187<br>92,416 (60.7%)	149,984<br>91,626 (61.1%)	-	2,203<br>790 (35.9%)
Royal Ontario Museum	Canada	150,080<br>120,399 (80.2%)	-	-	150,080<br>120,399 (80.2%)
Israel Nature and Parks Authority	Israel / EU - BioCASE	134,076<br>101,540 (75.7%)	-	134,076<br>101,540 (75.7%)	-
Field Museum	USA	122,457<br>107,377 (87.7%)	-	-	122,457<br>107,377 (87.7%)
Canadian Biodiversity Information Facility	Canada	120,384<br>97,427 (80.9%)	120,384<br>97,427 (80.9%)	-	-
Museum of Comparative Zoology, Harvard University	USA	115,101<br>96,997 (84.3%)	-	-	115,101<br>96,997 (84.3%)
Australian Museum	Australia	107,389<br>86,946 (81.0%)	-	-	107,389<br>86,946 (81.0%)
Scientific Committee on Antarctic Research - Marine Biodiversity Information Network (SCAR-MarBIN)	International	104,527<br>8 (0.0%)	427<br>8 (1.9%)	104,100<br>0 (0.0%)	-
Canadian Museum of Nature	Canada	88,218<br>73,846 (83.7%)	-	-	88,218<br>73,846 (83.7%)
Comisión nacional para el conocimiento y uso de la biodiversidad (CONABIO)	Mexico	83,925<br>71,716 (85.5%)	65,111<br>55,757 (85.6%)	18,814<br>15,959 (84.8%)	-
University of Washington Burke Museum	USA	72,535<br>53,763 (74.1%)	-	-	72,535<br>53,763 (74.1%)
BeBIF Provider	Belgium	70,010<br>63,116 (90.2%)	41,033<br>35,940 (87.6%)	28,977<br>27,176 (93.8%)	-
TELDAP	Chinese Taipei	67,664<br>63,208 (93.4%)	-	67,664<br>63,208 (93.4%)	-
California Academy of Sciences	USA	63,523<br>54,871 (86.4%)	-	-	63,523<br>54,871 (86.4%)
Western Foundation of Vertebrate Zoology	USA	60,798<br>53,468 (87.9%)	-	-	60,798<br>53,468 (87.9%)
CSIRO	Australia	60,192<br>52,126 (86.6%)	12<br>0 (0.0%)	-	60,180<br>52,114 (86.6%)
Dpt. of Environment and Resource Management (Queensland)	Australia	58,653<br>31,921 (%)	-	58,287<br>31,611 (54.2%)	366<br>310 (84.7%)

**Data publisher**	**Country**	**Records total / valid (% of total)**	**Unknown total / valid (% of total)**	**Observations total / valid (% of total)**	**Specimens total / valid (% of total)**
Taiwan Biodiversity Information Facility (TaiBIF)	Chinese Taipei	57,172<br>31,806 (55.6%)	-	57,172<br>31,806 (55.6%)	-
EMAN Provider	Canada	48,889<br>3,147 (6.4%)	48,889<br>3,147 (6.4%)	-	-
Natural History Museum, University of Oslo	Norway	48,659<br>16,262 (33.4%)	-	-	48,659<br>16,262 (33.4%)
Museum Victoria	Australia	45,922<br>36,033 (78.5%)	-	-	45,922<br>36,033 (78.5%)
Institute of Nature Conservation, Polish Academy of Sciences	Poland	45,373<br>44,633 (98.4%)	-	45,373<br>44,633 (98.4%)	-
British Antarctic Survey	UK	45,008<br>6 (0.0%)	30<br>1 (3.3%)	44,978<br>5 (0.0%)	-
GEO-Tag der Artenvielfalt	Germany	41,313<br>38,022 (92.0%)	-	41,291<br>38,007 (92.0%)	22<br>15 (68.2%)
Delaware Museum of Natural History	USA	39,111<br>35,247 (90.1%)	-	-	39,111<br>35,247 (90.1%)
South Australian Museum	Australia	36,888<br>30,382 (82.4%)	36,888<br>30,382 (82.4%)	-	-
San Diego Natural History Museum	USA	35,664<br>30,532 (67.5%)	-	-	35,664<br>30,532 (67.5%)
University of Kansas Biodiversity Institute	USA	35,334<br>23,868 (67.5%)	-	-	35,334<br>23,868 (67.5)
Natural History Museum of Los Angeles County	USA	33,933<br>28,805 (84.9%)	-	-	33,933<br>28,805 (84.9%)
UCLA-Dickey Collection	USA	32,931<br>29,428 (89.4%)	1<br>1 (100.0%)	-	32,930<br>29,427 (89.4%)
Royal Belgian Institute of Natural Sciences	Belgium	30,121<br>24,272 (80.6%)	-	-	30,121<br>24,272 (80.6%)
Borror Laboratory of Bioacoustics	USA	29,983<br>27,778 (92.6%)	-	29,983<br>27,778 (92.6%)	-
Western Australian Museum	Australia	29,417<br>22,222 (75.5%)	-	-	29,417<br>22,222 (75.5%)
Administración de Parques Nacionales, Argentina	Argentina	27,466<br>21,656 (78.8%)	-	27,466<br>21,656 (78.8%)	-
American Museum of Natural History	USA	27,008<br>22,643 (83.8%)	-	-	27,008<br>22,643 (83.8%)
Biodiversitäts-Monitoring Schweiz - BDMCH	Switzerland	26,721<br>26,480 (99.1%)	-	26,721<br>26,480 (99.1%)	-
Cornell University Museum of Vertebrates	USA	24,338<br>20,500 (84.2%)	-	-	24,338<br>20,500 (84.2%)
UNIBIO, IBUNAM	Mexico	22,090<br>19,614 (88.8%)	22,090<br>19,614 (88.8%)	-	-
James R. Slater Museum of Natural History	USA	20,978<br>18,094 (86.3%)	-	-	20,978<br>18,094 (86.3%)
Santa Barbara Museum of Natural History	USA	19,178<br>16,311 (85.1%)	-	-	19,178<br>16,311 (85.1%)

Data publisher	Country	Records total / valid (% of total)	Unknown total / valid (% of total)	Observations total / valid (% of total)	Specimens total / valid (% of total)
Instituto de Investigación de Recursos Biológicos Alexander von Humboldt	Colombia	18,291<br>16,086 (87.9%)	17,047<br>14,845 (87.1%)	1,244<br>1,241 (99.8%)	-
Facultad de Ciencias, UNAM	Mexico	16,642<br>15,234 (91.5%)	16,642<br>15,234 (91.5%)	-	-
Conservation International	USA	15,678<br>14,433 (92.1%)	-	15,678<br>14,433 (92.1%)	-
Musée national d'histoire naturelle Luxembourg	Luxembourg	14,630<br>13,362 (91.3%)	-	14,630<br>13,362 (91.3%)	-
Instituto de Ciencias Naturales	Colombia	12,993<br>12,150 (93.5%)	-	-	12,993<br>12,150 (93.5%)
National Museum of Natural History	USA	12,824<br>7,005 (54.6%)	2<br>0 (0.0%)	-	12,822<br>7,005 (54.6%)
Museum fürNaturkunde Berlin	Germany	10,804<br>9,971 (92.3%)	-	10,779<br>9,946 (92.3%)	25<br>25 (100.0%)
Centre d'estudis de la neu i de la muntanya d'Andorra (CENMA), Institut d'Estudis Andorrans	Andorra	10,120<br>9,876 (97.6%)	-	10,120<br>9,876 (97.6%)	-
University of Nebraska State Museum	USA	9,581<br>8,310 (86.7%)	-	-	9,581<br>8,310 (86.7%)
Jagiellonian University, Institute of Environmental Sciences	Poland	8,460<br>7,898 (93.4%)	-	8,460<br>7,898 (93.4%)	-
Upper Silesian Museum, Bytom	Poland	8,403<br>5,241 (62.4%)	-	8,403<br>5,241 (62.4%)	-
Museo Argentino de Ciencias Naturales	Argentina	8,145<br>6,997 (85.9%)	-	-	8,145<br>6,997 (85.9%)
New Brunswick Museum	Canada	7,911<br>6,324 (79.9%)	7,911<br>6,324 (79.9%)	-	-
Bernice Pauahi Bishop Museum	USA	7,741<br>5,330 (68.9%)	-	-	7,741<br>5,330 (68.9%)
Corantioquia	Colombia	7,057<br>6,238 (88.4%)	-	7,057<br>6,238 (88.4%)	-
inatura – Erlebnis Naturschau Dornbirn	Austria	6,319<br>6,098 (96.5%)	6,319<br>6,098 (96.5%)	-	-
National Museum of Nature and Science, Japan	Japan	5,956<br>4,543 (76.3%)	-	-	5,956<br>4,543 (76.3%)
Ireland?	Ireland?	5,913<br>5,078 (85.9%)	-	-	5,913<br>5,078 (85.9%)
Queen Victoria Museum and Art Gallery	Australia	5,585<br>4,143 (74.2%)	5,585<br>4,143 (74.2%)	-	-
iNaturalist,org	USA	5,325<br>4,684 (88.0%)	-	5,325<br>4,684 (88.0%)	-
Netherlands Biodiversity Information Facility (NLBIF)	Netherlands	4,779<br>806 (16.9%)	-	-	4,779<br>806 (16.9%)
Isagen	Colombia	4,135<br>3,895 (94.2%)	11<br>11 (100.0%)	4,124<br>3,884 (94.2%)	-
Haus der Natur Salzburg	Austria	3,752<br>3,749 (99.9%)	3,752<br>3,749 (99.9%)	-	-

Data publisher	Country	Records total / valid (% of total)	Unknown total / valid (% of total)	Observations total / valid (% of total)	Specimens total / valid (% of total)
National Science Museum of Korea	Korea, Republic of	3,715<br>2,589 (69.7%)	2,660<br>1,909 (71.8%)	-	1,055<br>680 (64.5%)
Tasmanian Museum and Art Gallery	Australia	3,355<br>2,044 (60.9%)	1<br>1 (100.0%)	28<br>16 (57.1%)	3,326<br>2,027 (60.9%)
Senckenberg	Germany	3,116<br>2,618 (84.0%)	-	-	3,116<br>2,618 (84.0%)
University of Colorado Museum of Natural History	USA	3,068<br>2,515 (82.0%)	-	-	3,068<br>2,515 (82.0%)
Mokpo Museum of Natural History	Korea, Republic of	2,630<br>1,525 (58.0%)	2,605<br>1,514 (58.1%)	-	25<br>11 (44.0%)
Illinois State University	USA	2,457<br>2,006 (81.6%)	-	-	2,457<br>2,006 (81.6%)
Tall Timbers Research Station and Land Conservancy	USA	2,407<br>2,071 (86.0%)	2,407<br>2,071 (86.0%)	-	-
Natural History Museum, University of Tartu	Estonia	1,794<br>1,784 (99.4%)	-	-	1,794<br>1,784 (99.4%)
Citizen Science - ALA Website	Australia	1,543<br>1,458 (94.5%)	1,543<br>1,458 (94.5%)	-	-
Cincinnati Museum Center	USA	1,009<br>920 (91.2%)	1,009<br>920 (91.2%)	-	-
Wildlife Institute of India	India	752<br>606 (80.6%)	752<br>606 (80.6%)	-	-
PANGAEA - Publishing Network for Geoscientific and Environmental Data	Germany	673<br>240 (35.7%)	-	673<br>240 (35.7%)	-
National Chemical Laboratory (via OBIS)	International	647<br>285 (44.0%)	647<br>285 (44.0%)	-	-
European Molecular Biology Laboratory Australia	Australia	631<br>549 (87.0%)	631<br>549 (87.0%)	-	-
University of Alberta Museums	Canada	476<br>331 (69.5%)	-	-	476<br>331 (69.5%)
Ohio State University Insect Collection	USA	469<br>456 (97.2%)	-	-	469<br>456 (97.2%)
Wildlife Conservation Society - Madagascar Program (WCS - Mad)	Madagascar	469<br>460 (98.1%)	-	469<br>460 (98.1%)	-
Field Study Group of the Dutch Mammal Society	Netherlands	445<br>321 (72.1%)	-	445<br>321 (72.1%)	-
Museé national d'Histoire naturelle	France	209<br>164 (78.5%)	-	-	209<br>164 (78.5%)
New Mexico Biodiversity Collections Consortium	USA	199<br>177 (88.9%)	-	-	199<br>177 (88.9%)
SysTax	Germany	199<br>140 (70.4%)	199<br>140 (70.4%)	-	-
Wildlife Sightings	Canada	189<br>177 (93.7%)	-	189<br>177 (93.7%)	-
Queensland Museum	Australia	183<br>177 (96.7%)	-	-	183<br>177 (96.7%)

Data publisher	Country	Records total / valid (% of total)	Unknown total / valid (% of total)	Observations total / valid (% of total)	Specimens total / valid (% of total)
Botanic Garden and Botanical Museum Berlin-Dahlem	Germany	163<br>108 (66.3%)	-	115<br>82 (71.3%)	48<br>26 (54.2%)
Jagiellonian University, Institute of Zoology	Poland	137<br>70 (51.1%)	-	137<br>70 (51.1%)	-
University of Navarra, Museum of Zoology	Spain	105<br>85 (81.0%)	-	-	105<br>85 (81.0%)
Gyeryonsan Natural History Museum	Korea, Republic of	53<br>23 (43.4%)	-	-	53<br>23 (43.4%)
University of Helsinki, Dpt. of Applied Biology	Finland	45<br>41 (91.1%)	-	45<br>41 (91.1%)	-
Michigan State University Museum	USA	9<br>9 (100.0%)	-	-	9<br>9 (100.0%)
Nicolaus Copernicus University of Toruń	Poland	6<br>6 (100.0%)	-	6<br>6 (100.0%)	-
Humboldt State University	USA	5<br>5 (100.0%)	-	-	5<br>5 (100.0%)
Mammal Research Institute, Polish Academy of Sciences	Poland	4<br>4 (100.0%)	44<br>4 (100.0%)	-	-
Sam Noble Oklahoma Museum of Natural History	USA	2<br>0 (0%)	2<br>0 (0%)	-	-
Jyvaskyla University Museum	Finland	1<br>1 (100.0%)	-	-	1<br>1 (100.0%)
University of Silesia, Herbarium KTU	Poland	1<br>1 (100.0%)	-	-	1<br>1 (100.0%)

b) Publishers of mammal records

Data publisher	Country	Records total / valid (% of total)	Unknown total / valid (% of total)	Observations total / valid (% of total)	Specimens total / valid (% of total)
UK National Biodiversity Network	UK	521,021<br>396,214 (76.0%)	521,021<br>396,214 (76.0%)	-	-
Arctos	USA	455,737<br>401,284 (88.1%)	-	-	455,737<br>401,284 (88.1%)
NSW Dpt. of Environment, Climate Change, and Water	Australia	375,532<br>306,596 (81.6%)	-	375,532<br>306,596 (81.6%)	-
Service du Patrimoine naturel, Musée national d'Histoire naturelle, Paris	France	334,434<br>258,876 (77.4%)	-	334,434<br>258,876 (77.4%)	-
Australian Antarctic Data Centre	Australia	289,554<br>0 (0%)	119,930<br>0 (0%)	169,624<br>0 (0%)	-
Ocean Biogeographic Information System (via OBIS)	International	262,463<br>2082(0.8%)	3,874<br>1(0.0%)	258,219<br>2081(0.8%)	370<br>0 (0%)
University of Kansas Biodiversity Institute	USA	159,667<br>144,186 (90.3%)	-	-	159,667<br>144,186 (90.3%)

Data publisher	Country	Records total / valid (% of total)	Unknown total / valid (% of total)	Observations total / valid (% of total)	Specimens total / valid (% of total)
Field Museum	USA	156,235<br>132,015 (84.5%)	-	-	156,235<br>132,015 (84.5%)
Comisión nacional para el conocimiento y uso de la biodiversidad	Mexico	153,422<br>130,345 (85.0%)	147,755<br>125,501 (84.9%)	5,667<br>4,844 (85.5%)	-
South Australia, Department of Environment and Natural Resources	Australia	125,906<br>92,962 (73.8%)	31<br>20 (64.5%)	120,168<br>88,613 (73.7%)	5,707<br>4,329 (75.9%)
GBIF-Spain	Spain	103,041<br>87,740 (85.2%)	-	99,615<br>85,978 (86.3%)	3,426<br>1,762 (51.4%)
National Museum of Natural History	USA	98,159<br>82,376 (83.9%)	-	8<br>0 (0%)	98,151<br>82,376 (83.9%)
Mammal Research Institute, Polish Academy of Sciences	Poland	86,239<br>82,915 (96.1%)	753<br>747 (99.2%)	-	85,486<br>82,168 (96.1%)
Natural History Museum of Los Angeles County	USA	79,770<br>68,834 (86.3%)	-	-	79,770<br>68,834 (86.3%)
National Biodiversity Data Centre	Ireland	73,067<br>62,727 (85.8%)	-	-	73,067<br>62,727 (85.8%)
Australian Museum	Australia	71,124<br>54,736 (77.0%)	-	-	71,124<br>54,736 (77.0%)
BeBIF Provider	Belgium	69,848<br>62,665 (89.7%)	-	5,763<br>4,764 (82.7%)	64,085<br>57,901 (90.4%)
University of Navarra, Museum of Zoology	Spain	60,888<br>55,009 (90.3%)	-	1,878<br>1,858 (98.9%)	59,010<br>53,151 (90.1%)
Dpt. of Natural Resources, Environment, The Arts and Sport, Northern Territory of Australia	Australia	56,085<br>33,864 (60.4%)	-	56,085<br>33,864 (60.4%)	-
University of Washington Burke Museum	USA	53,415<br>37,178 (69.6%)	-	-	53,415<br>37,178 (69.6%)
James R. Slater Museum of Natural History	USA	49,585<br>45,673 (92.1%)	-	-	49,585<br>45,673 (92.1%)
Western Australian Museum	Australia	44,644<br>35,351 (79.2%)	-	-	44,644<br>35,351 (79.2%)
Scientific Committee on Antarctic Research - Marine Biodiversity Information Network (SCAR-MarBIN)	International	41,863<br>0 (0%)	41,739<br>0 (0%)	124<br>0 (0%)	-
Sam Noble Oklahoma Museum of Natural History	USA	36,269<br>25,681 (70.8%)	36,269<br>25,681 (70.8%)	-	-
Royal Belgian Institute of Natural Sciences	Belgium	32,736<br>29,287 (89.5%)	-	-	32,736<br>29,287 (89.5%)
CSIRO	Australia	31,727<br>25,205 (79.4%)	4,503<br>3,521 (78.2%)	-	27,224<br>21,684 (79.7%)
Israel Nature and Parks Authority	Israel / EU - BioCASE	30,754<br>25,909 (84.2%)	-	30,754<br>25,909 (84.2%)	-
UNIBIO, IBUNAM	Mexico	30,197<br>25,149 (83.3%)	30,197<br>25,149 (83.3%)	-	-
Museum Victoria	Australia	28,568<br>22,947 (80.3%)	-	-	28,568<br>22,947 (80.3%)
Louisiana State University Museum of Natural Science	USA	27,866<br>23,784 (85.4%)	-	-	27,866<br>23,784 (85.4%)

Data publisher	Country	Records total / valid (% of total)	Unknown total / valid (% of total)	Observations total / valid (% of total)	Specimens total / valid (% of total)
Michigan State University Museum	USA	27,768<br>24,803 (89.3%)	-	-	27,768<br>24,803 (89.3%)
ArtDatabanken	Sweden	27,674<br>22,403 (81.0%)	-	27,674<br>22,403 (81.0%)	-
South Australian Museum	Australia	23,997<br>15,298 (63.7%)	23,456<br>15,062 (64.2%)	134<br>23 (17.2%)	407<br>213 (52.3%)
California Academy of Sciences	USA	23,411<br>18,965 (81.0%)	-	-	23,411<br>18,965 (81.0%)
Danish Biodiversity Information Facility	Denmark	21,549<br>10,863 (50.4%)	-	21,469<br>10,797 (50.3%)	80<br>66 (82.5%)
Administración de Parques Nacionales, Argentina	Argentina	19,136<br>13,891 (72.6%)	117<br>96 (82.1%)	12,739<br>8,035 (63.1%)	6,280<br>5,760 (91.7%)
Natural History Museum, University of Oslo	Norway	18,499<br>9,345 (50.5%)	362<br>16(4.4%)	1<br>0 (0%)	18,136<br>9,329 (51.4%)
The Norwegian Biodiversity Information Centre (NBIC)	Norway	18,314<br>15,914 (86.9%)	-	18,314<br>15,914 (86.9%)	-
British Antarctic Survey	UK	17,341<br>0 (0%)	15<br>0 (0%)	17,326<br>0 (0%)	-
UCLA-Dickey Collection (UCLA-Dickey)	USA	16,553<br>14,106 (85.2%)	-	-	16,553<br>14,106 (85.2%)
Museo Argentino de Ciencias Naturales	Argentina	14,514<br>10,265 (70.7%)	-	-	14,514<br>10,265 (70.7%)
Finnish Museum of Natural History	Finland	14,469<br>8,874 (61.3%)	-	14,469<br>8,874 (61.3%)	-
New York State Museum (NYSM)	USA	13,388<br>12,667 (94.6%)	-	-	13,388<br>12,667 (94.6%)
Yale University Peabody Museum	USA	11,881<br>9,565 (80.5%)	-	-	11,881<br>9,565 (80.5%)
Musée national d'histoire naturelle Luxembourg	Luxembourg	11,754<br>11,033 (93.9%)	-	11,754<br>11,033 (93.9%)	-
New Mexico Biodiversity Collections Consortium	USA	11,679<br>10,752 (92.1%)	-	-	11,679<br>10,752 (92.1%)
Santa Barbara Museum of Natural History	USA	9,633<br>7,773 (80.7%)	-	-	9,633<br>7,773 (80.7%)
PANGAEA - Publishing Network for Geoscientific and Environmental Data	Germany	7,884<br>3,526 (44.7%)	-	7,884<br>3,526 (44.7%)	-
American Museum of Natural History	USA	7,704<br>6,603 (85.7%)	-	-	7,704<br>6,603 (85.7%)
University of Colorado Museum of Natural History	USA	7,598<br>7,087 (93.3%)	-	-	7,598<br>7,087 (93.3%)
University of Warsaw, Dpt. of Ecology	Poland	6,834<br>6,673 (97.6%)	489<br>352 (72.0%)	6,345<br>6,321 (99.6%)	-
inatura – Erlebnis Naturschau Dornbirn	Austria	6,068<br>6,061 (99.9%)	6068<br>6061 (99.9%)	-	-
Museum of Comparative Zoology, Harvard University	USA	5,200<br>3,855 (74.1%)	-	-	5,200<br>3,855 (74.1%)

Data publisher	Country	Records total / valid (% of total)	Unknown total / valid (% of total)	Observations total / valid (% of total)	Specimens total / valid (% of total)
Instituto de Ciencias Naturales	Colombia	4,985<br>4,500 (90.3%)	-	-	4,985<br>4,500 (90.3%)
Queen Victoria Museum and Art Gallery	Australia	4,693<br>4,087 (87.1%)	4693<br>4087 (87.1%)	-	-
Texas Cooperative Wildlife Collection	USA	4,586<br>4,326 (94.3%)	-	-	4,586<br>4,326 (94.3%)
Centre d'estudis de la neu i de la muntanya d'Andorra (CENMA), Institut d'Estudis Andorrans	Andorra	4,410<br>4,323 (98.0%)	-	4,410<br>4,323 (98.0%)	-
TELDAP	Chinese Taipei	3,643<br>3,405 (93.5%)	-	3,641<br>3,403 (93.5%)	2<br>2 (100.0%)
New Mexico Museum of Natural History and Science	USA	3,270<br>170(5.2%)	-	-	3,270<br>170(5.2%)
Cornell University Museum of Vertebrates	USA	2,983<br>2,733 (91.6%)	-	-	2,983<br>2,733 (91.6%)
Conservation International	USA	2,734<br>2,345 (85.8%)	-	2,734<br>2,345 (85.8%)	-
Tasmanian Museum and Art Gallery	Australia	2,710<br>1,273 (47.0%)	-	67<br>51 (76.1%)	2,643<br>1,222 (46.2%)
Bernice Pauahi Bishop Museum	USA	2,512<br>1,457 (58.0%)	-	-	2,512<br>1,457 (58.0%)
Avian Knowledge Network	USA	2,438<br>470 (19.3%)	-	2,438<br>470 (19.3%)	-
Field Study Group of the Dutch Mammal Society	Netherlands	2,167<br>2,010 (92.8%)	-	2,167<br>2,010 (92.8%)	-
GEO-Tag der Artenvielfalt	Germany	1,987<br>1,776 (89.4%)	-	1,987<br>1,776 (89.4%)	-
GBIF-Sweden	Sweden	1,961<br>898 (45.8%)	-	451<br>78 (17.3%)	1,510<br>820 (54.3%)
Instituto de Investigación de Recursos Biológicos Alexander von Humboldt	Colombia	1,910<br>1,820 (95.3%)	897<br>807 (90.0%)	1,013<br>1013 (100.0%)	-
Corantioquia	Colombia	1,735<br>1,168 (67.3%)	-	1,735<br>1,168 (67.3%)	-
Dutch Mammal Society	Netherlands	1,626<br>0 (0%)	-	1,626<br>0 (0%)	-
EMAN Provider	Canada	1,414<br>14(1.0%)	1,414<br>14(1.0%)	-	-
Museum für Naturkunde Berlin	Germany	1,404<br>652 (46.4%)	-	1,378<br>628 (45.6%)	26<br>24 (92.3%)
Institute of Research for Development	France	1,321<br>0 (0%)	-	1,321<br>0 (0%)	-
United States Geological Survey	USA	1,136<br>3(0.3%)	-	1,124<br>3(0.3%)	12<br>0 (0%)
Institute of Nature Conservation, Polish Academy of Sciences	Poland	1,113<br>825 (74.1%)	-	1,113<br>825 (74.1%)	-
Borror Laboratory of Bioacoustics	USA	1,041<br>426 (40.9%)	-	1,041<br>426 (40.9%)	-

Data publisher	Country	Records total / valid (% of total)	Unknown total / valid (% of total)	Observations total / valid (% of total)	Specimens total / valid (% of total)
University of Michigan Museum of Zoology	USA	1,034<br>1,009 (97.6%)	-	-	1,034<br>1,009 (97.6%)
Isagen	Colombia	1,031<br>722 (70.0%)	10<br>6 (60.0%)	1,021<br>716 (70.1%)	-
iNaturalist.org	USA	1,018<br>833 (81.8%)	-	1,018<br>833 (81.8%)	-
Natural History Museum, University of Tartu	Estonia	996<br>914 (91.8%)	-	-	996<br>914 (91.8%)
Association for Nature WOLF	Poland	987<br>878 (89.0%)	-	987<br>878 (89.0%)	-
Illinois State University	USA	827<br>735 (88.9%)	-	-	827<br>735 (88.9%)
University of Alberta Museums	Canada	822<br>551 (67.0%)	-	-	822<br>551 (67.0%)
KBIF Data Repository	Korea, Republic of	806<br>622 (77.2%)	746<br>602 (80.7%)	-	60<br>20 (33.3%)
National Museum of Nature and Science, Japan	Japan	309<br>278 (90.0%)	-	-	309<br>278 (90.0%)
Ohio State University Insect Collection	USA	253<br>195 (77.1%)	-	-	253<br>195 (77.1%)
European Forest Institute	Finland	226<br>220 (97.3%)	226<br>220 (97.3%)	-	-
Wildlife Conservation Society - Madagascar Program (WCS - Mad)	Madagascar	189<br>173 (91.5%)	-	189<br>173 (91.5%)	-
University of Minnesota Bell Museum of Natural History	USA	172<br>172 (100.0%)	-	-	172<br>172 (100.0%)
Citizen Science - ALA Website	Australia	168<br>143 (85.1%)	168<br>143 (85.1%)	-	-
Queensland Museum	Australia	136<br>121 (89.0%)	-	-	136<br>121 (89.0%)
National Chemical Laboratory (via OBIS)	International	127<br>33 (26.0%)	127<br>33 (26.0%)	-	-
Haus der Natur Salzburg	Austria	108<br>108 (100.0%)	108<br>108 (100.0%)	-	-
Geocollections of Estonia	Estonia	67<br>3(4.5%)	67<br>3(4.5%)	-	-
Botanic Garden and Botanical Museum Berlin-Dahlem	Germany	46<br>37 (80.4%)	-	17<br>14 (82.4%)	29<br>23 (79.3%)
University of Helsinki, Dpt. of Applied Biology	Finland	39<br>35 (89.7%)	-	39<br>35 (89.7%)	-
Netherlands Biodiversity Information Facility (NLBIF)	Netherlands	34<br>3(8.8%)	-	-	34<br>3(8.8%)
National Science Museum of Korea	Korea, Republic of	31<br>20 (64.5%)	-	-	31<br>20 (64.5%)
Jagiellonian University, Institute of Zoology	Poland	30<br>17 (56.7%)	-	30<br>17 (56.7%)	-

Data publisher	Country	Records total / valid (% of total)	Unknown total / valid (% of total)	Observations total / valid (% of total)	Specimens total / valid (% of total)
Staatliche Naturwissenschaftliche Sammlungen Bayerns	Germany	27<br>26 (96.3%)	-	-	27<br>26 (96.3%)
Wildlife Sightings	Canada	25<br>12 (48.0%)	-	25<br>12 (48.0%)	-
European Molecular Biology Laboratory Australia	Australia	22<br>11 (50.0%)	22<br>11 (50.0%)	-	-
Senckenberg	Germany	20<br>2 (10.0%)	-	-	20<br>2 (10.0%)
University of Nebraska State Museum	USA	11<br>7 (63.6%)	-	-	11<br>7 (63.6%)
Biologiezentrum Linz Oberösterreich	Austria	7<br>1 (14.3%)	7<br>1 (14.3%)	-	-
University of Silesia, Laboratory of Botanical Documentation - Herbarium KTU	Poland	6<br>6 (100.0%)	-	-	6<br>6 (100.0%)
Museum of Texas Tech University (TTU)	USA	5<br>5 (100.0%)	-	-	5<br>5 (100.0%)
Upper Silesian Museum, Bytom	Poland	4<br>3 (75.0%)	-	-	4<br>3 (75.0%)
South African National Biodiversity Institute	South Africa	4<br>1 (25.0%)	-	-	4<br>1 (25.0%)
University of Texas at El Paso	USA	2<br>1 (50.0%)	-	-	2<br>1 (50.0%)
IHAR	Poland	1<br>0 (0%)	-	1<br>0 (0%)	-
Nicolaus Copernicus University of Toruń	Poland	1<br>0 (0%)	-	1<br>0 (0%)	-
University of Gdańsk, Bird Migration Research Station	Poland	1<br>0 (0%)	-	1<br>0 (0%)	-
University of Gdańsk, Dpt. of Plant Taxonomy and Nature Conservation	Poland	1<br>0 (0%)	-	-	1<br>0 (0%)
Royal Ontario Museum	Canada	1<br>1 (100.0%)	-	-	1<br>1 (100.0%)

c) Publishers of amphibian records

Data publisher	Country	Records total / valid (% of total)	Unknown total / valid (% of total)	Observations total / valid (% of total)	Specimens total / valid (% of total)
National Museum of Natural History	USA	233,924<br>198,468 (84.8%)	2<br>2 (100.0%)	-	233,922<br>198,466 (84.8%)
Arctos	USA	136,381<br>120,466 (88.3%)	-	-	136,381<br>120,466 (88.3%)
Museum of Comparative Zoology, Harvard University	USA	98,370<br>77,722 (79.0%)	-	-	98,370<br>77,722 (79.0%)

Supplementary Table 9 (continued)

Data publisher	Country	Records total / valid (% of total)	Unknown total / valid (% of total)	Observations total / valid (% of total)	Specimens total / valid (% of total)
UK National Biodiversity Network	UK	96,559<br>94,502 (97.9%)	96,559<br>94,502 (97.9%)	-	-
California Academy of Sciences	USA	89,345<br>73,794 (82.6%)	-	-	89,345<br>73,794 (82.6%)
Australian Museum	Australia	85,814<br>71,155 (82.9%)	-	-	85,814<br>71,155 (82.9%)
NSW Dpt. of Environment, Climate Change, and Water	Australia	72,921<br>61,468 (84.3%)	-	72,921<br>61,468 (84.3%)	-
Chengdu Institute of Biology, Chinese Academy of Science	Chinese Taipei	58,164<br>48,396 (83.2%)	-	-	58,164<br>48,396 (83.2%)
Natural History Museum of Los Angeles County	USA	43,768<br>37,214 (85.0%)	-	-	43,768<br>37,214 (85.0%)
GBIF-Spain	Spain	38,174<br>35,623 (93.3%)	-	28,393<br>27,058 (95.3%)	9,781<br>8,565 (87.6%)
Museum Victoria	Australia	34,845<br>31,303 (89.8%)	-	-	34,845<br>31,303 (89.8%)
Comisión nacional para el conocimiento y uso de la biodiversidad	Mexico	28,282<br>20,774 (73.5%)	24,616<br>17,712 (72.0%)	3,666<br>3,062 (83.5%)	-
South Australia, Department of Environment and Natural Resources	Australia	25,147<br>23,611 (93.9%)	-	24,340<br>22,945 (94.3%)	807<br>666 (82.5%)
Musée d'histoire naturelle de la Ville de Genève - MHNG	Switzerland	24,894<br>22,218 (89.3%)	-	-	24,894<br>22,218 (89.3%)
Bird Studies Canada	Canada	24,856<br>18,852 (75.8%)	-	24,856<br>18,852 (75.8%)	-
Western Australian Museum	Australia	23,294<br>20,508 (88.0%)	-	-	23,294<br>20,508 (88.0%)
Royal Ontario Museum	Canada	23,182<br>19,307 (83.3%)	-	-	23,182<br>19,307 (83.3%)
ArtDatabanken	Sweden	18,660<br>16,196 (86.8%)	-	18,660<br>16,196 (86.8%)	-
University of Kansas Biodiversity Institute	USA	18,2533<br>24,438 (13.4%)	18,2533<br>24,438 (13.4%)	-	-
Canadian Museum of Nature	Canada	17,371<br>12,232 (70.4%)	-	-	17,371<br>12,232 (70.4%)
Service du Patrimoine naturel, Musée national d'Histoire naturelle, Paris	France	16,352<br>14,665 (89.7%)	-	16,352<br>14,665 (89.7%)	-
Instituto de Ciencias Naturales	Colombia	14,626<br>12,749 (87.2%)	-	-	14,626<br>12,749 (87.2%)
Yale University Peabody Museum	USA	13,682<br>12,082 (88.3%)	-	-	13,682<br>12,082 (88.3%)

Supplementary Table 9 (continued)

Data publisher	Country	Records total / valid (% of total)	Unknown total / valid (% of total)	Observations total / valid (% of total)	Specimens total / valid (% of total)
Museo Argentino de Ciencias Naturales	Argentina	13,055<br>10,249 (78.5%)	-	-	13,055<br>10,249 (78.5%)
South Australian Museum	Australia	13,031<br>11,000 (84.4%)	13,031<br>11,000 (84.4%)	-	-
New Mexico Biodiversity Collections Consortium	USA	12,049<br>10,257 (85.1%)	-	-	12,049<br>10,257 (85.1%)
Museum of Southwestern Biology, Division of Amphibians and Reptiles	USA	11,255<br>9,579 (85.1%)	11,255<br>9,579 (85.1%)	-	-
Dpt. of Natural Resources, Environment, The Arts and Sport, Northern Territory of Australia	Australia	10,808<br>9,334 (86.4%)	-	10,808<br>9,334 (86.4%)	-
San Diego Natural History Museum	USA	10,617<br>8,354 (78.7%)	-	-	10,617<br>8,354 (78.7%)
CSIRO	Australia	9,190<br>7,579 (82.5%)	6,290<br>4,950 (78.7%)	-	2,900<br>2,629 (90.7%)
Cornell University Museum of Vertebrates	USA	9,078<br>7,915 (87.2%)	-	-	9,078<br>7,915 (87.2%)
Alabama Museum of Natural History	USA	8,931<br>7,325 (82.0%)	8,931<br>7,325 (82.0%)	-	-
South African National Biodiversity Institute	South Africa	7,107<br>6,491 (91.3%)	-	-	7,107<br>6,491 (91.3%)
Musée national d'histoire naturelle Luxembourg	Luxembourg	6,997<br>5,320 (76.0%)	-	6,997<br>5,320 (76.0%)	-
Bernice Pauahi Bishop Museum	USA	6,853<br>5,251 (76.6%)	-	-	6,853<br>5,251 (76.6%)
EMAN Provider	Canada	6,639<br>5,090 (76.7%)	6,639<br>5,090 (76.7%)	-	-
TELDAP	Chinese Taipei	6,596<br>6,379 (96.7%)	-	6,596<br>6,379 (96.7%)	-
Royal Belgian Institute of Natural Sciences	Belgium	6,560<br>4,961 (75.6%)	-	-	6,560<br>4,961 (75.6%)
Danish Biodiversity Information Facility	Denmark	6,274<br>4,968 (79.2%)	498<br>498 (100.0%)	3,422<br>2,643 (77.2%)	2,354<br>1,827 (77.6%)
Natural History Museum, University of Oslo	Norway	6,221<br>5,065 (81.4%)	-	5,253<br>4,615 (87.9%)	968<br>450 (46.5%)
Sternberg Museum of Natural History	USA	5,110<br>3,447 (67.5%)	-	-	5,110<br>3,447 (67.5%)
Zoological Institute, Russian Academy of Sciences, St. Petersburg (via the Society for the Management of Electronic Biodiversity Data)	Russia	4,534<br>3,285 (72.5%)	4,534<br>3,285 (72.5%)	-	-
National Biodiversity Data Centre	Ireland	4,033<br>4,032 (100.0%)	-	-	4,033<br>4,032 (100.0%)

Supplementary Table 9 (continued)

Data publisher	Country	Records total / valid (% of total)	Unknown total / valid (% of total)	Observations total / valid (% of total)	Specimens total / valid (% of total)
James R. Slater Museum of Natural History	USA	3,843<br>3,303 (85.9%)	-	-	3,843<br>3,303 (85.9%)
UNIBIO, IBUNAM	Mexico	3,490<br>257(7.4%)	418<br>257 (61.5%)	-	3,072<br>0 (0%)
Institute of Nature Conservation, Polish Academy of Sciences	Poland	3,185<br>2,377 (74.6%)	-	3,185<br>2,377 (74.6%)	-
Cincinnati Museum Center	USA	3,005<br>2,607 (86.8%)	3,005<br>2,607 (86.8%)	-	-
KBIF Data Repository	Korea, Republic of	3,396<br>3,074 (90.5%)	3,396<br>3,074 (90.5%)	-	-
University of Alberta Museums	Canada	2,679<br>2,413 (90.1%)	-	-	2,679<br>2413 (90.1%)
Finnish Museum of Natural History	Finland	2,514<br>791 (31.5%)	-	2,514<br>791 (31.5%)	-
Raffles Museum of Biodiversity Research	BioNET-ASEANET	2,439<br>2,089 (85.6%)	-	-	2,439<br>2089 (85.6%)
University of Colorado Museum of Natural History	USA	2,118<br>1,661 (78.4%)	-	-	2,118<br>1,661 (78.4%)
Administración de Parques Nacionales, Argentina	Argentina	2,010<br>1,699 (84.5%)	-	99<br>41 (41.4%)	1,911<br>1,658 (86.8%)
University of Warsaw, Dpt. of Ecology	Poland	1,945<br>3(0.2%)	-	1,945<br>3 (0.2%)	-
The Norwegian Biodiversity Information Centre (NBIC)	Norway	1,872<br>1,622 (86.6%)	-	1,872<br>1,622 (86.6%)	-
Sam Noble Oklahoma Museum of Natural History	USA	1,770<br>706 (39.9%)	1,770<br>706 (39.9%)	-	-
United States Geological Survey	USA	1,752<br>172(9.8%)	-	1,067<br>93 (8.7%)	685<br>79 (11.5%)
Haus der Natur Salzburg	Austria	1,741<br>818 (47.0%)	1,741<br>818 (47.0%)	-	-
Białowieża National Park	Poland	1,723<br>679 (39.4%)	-	1,723<br>679 (39.4%)	-
Conservation International	USA	1,460<br>1,159 (79.4%)	-	1,460<br>1,159 (79.4%)	-
Royal Museum for Central Africa, Belgium	Belgium	1,413<br>1,036 (73.3%)	-	-	1,413<br>1,036 (73.3%)
University of Nevada, Reno	USA	1,257<br>742 (59.0%)	-	-	1,257<br>742 (59.0%)
Redpath Museum, McGill University	Canada	1,113<br>919 (82.6%)	-	-	1,113<br>919 (82.6%)

Supplementary Table 9 (continued)

Data publisher	Country	Records total / valid (% of total)	Unknown total / valid (% of total)	Observations total / valid (% of total)	Specimens total / valid (% of total)
GEO-Tag der Artenvielfalt	Germany	1,113<br>742 (66.7%)	2<br>0 (0%)	1,111<br>742 (66.8%)	-
Staatliches Museum für Naturkunde Stuttgart	Germany	1,107<br>758 (68.5%)	-	-	1,107<br>758 (68.5%)
Queensland Museum	Australia	871<br>852 (97.8%)	-	-	871<br>852 (97.8%)
Santa Barbara Museum of Natural History	USA	744<br>619 (83.2%)	-	-	744<br>619 (83.2%)
Borror Laboratory of Bioacoustics	USA	721<br>326 (45.2%)	-	721<br>326 (45.2%)	-
Queen Victoria Museum and Art Gallery	Australia	716<br>708 (98.9%)	716<br>708 (98.9%)	-	-
Senckenberg	Germany	615<br>552 (89.8%)	-	-	615<br>552 (89.8%)
Isagen	Colombia	611<br>557 (91.2%)	-	611<br>557 (91.2%)	-
iNaturalist.org	USA	565<br>479 (84.8%)	-	565<br>479 (84.8%)	-
University of Navarra, Museum of Zoology	Spain	525<br>466 (88.8%)	-	25<br>23 (92.0%)	500<br>443 (88.6%)
Israel Nature and Parks Authority	Israel / EU - BioCASE	485<br>338 (69.7%)	-	485<br>338 (69.7%)	-
Instituto de Investigación de Recursos Biológicos Alexander von Humboldt	Colombia	400<br>338 (84.5%)	-	400<br>338 (84.5%)	-
Netherlands Biodiversity Information Facility (NLBIF)	Netherlands	373<br>220 (59.0%)	6<br>6 (100.0%)	367<br>214 (58.3%)	-
GBIF-Sweden	Sweden	326<br>230 (70.6%)	-	104<br>84 (80.8%)	222<br>146 (65.8%)
Museum für Naturkunde Berlin	Germany	283<br>188 (66.4%)	-	283<br>188 (66.4%)	-
Avian Knowledge Network	USA	281<br>257 (91.5%)	-	281<br>257 (91.5%)	-
National Museum of Nature and Science, Japan	Japan	238<br>189 (79.4%)	-	-	238<br>189 (79.4%)
Milwaukee Public Museum	USA	215<br>102 (47.4%)	-	-	215<br>102 (47.4%)
Tasmanian Museum and Art Gallery	Australia	200<br>192 (96.0%)	-	4<br>3 (75.0%)	196<br>189 (96.4%)
Corantioquia	Colombia	141<br>91 (64.5%)	-	141<br>91 (64.5%)	-

Supplementary Table 9 (continued)

Data publisher	Country	Records total / valid (% of total)	Unknown total / valid (% of total)	Observations total / valid (% of total)	Specimens total / valid (% of total)
Wildlife Conservation Society - Madagascar Program	Madagascar	139<br>120 (86.3%)	-	139<br>120 (86.3%)	-
Field Study Group of the Dutch Mammal Society	Netherlands	135<br>114 (84.4%)	-	135<br>114 (84.4%)	-
American Museum of Natural History	USA	110<br>0 (0%)	-	-	110<br>0 (0%)
Centre d'estudis de la neu i de la muntanya d'Andorra (CENMA), Institut d'Estudis Andorrans	Andorra	106<br>72 (67.9%)	-	106<br>72 (67.9%)	-
Citizen Science - ALA Website	Australia	63<br>42 (66.7%)	63<br>42 (66.7%)	-	-
inatura – Erlebnis Naturschau Dornbirn	Austria	55<br>42 (76.4%)	55<br>42 (76.4%)	-	-
University of Minnesota Bell Museum of Natural History	USA	43<br>43 (100.0%)	-	-	43<br>43 (100.0%)
Wildlife Sightings	Canada	30<br>29 (96.7%)	-	30<br>29 (96.7%)	-
Zoologisches Forschungsinstitut und Museum Alexander Koenig	Germany	2<br>0 (0%)	-	-	2<br>0 (0%)
Botanic Garden and Botanical Museum Berlin-Dahlem	Germany	2<br>1 (50.0%)	-	2<br>1 (50.0%)	-
European Molecular Biology Laboratory Australia	Australia	17<br>17 (100.0%)	17<br>17 (100.0%)	-	-
SysTax	Germany	167<br>150 (89.8%)	167<br>150 (89.8%)	-	-
Michigan State University Museum	USA	16<br>16 (100.0%)	-	-	16<br>16 (100.0%)
National Chemical Laboratory (via OBIS)	International	10<br>2 (20.0%)	10<br>2 (20.0%)	-	-
Geocollections of Estonia	Estonia	1<br>0 (0%)	1<br>0 (0%)	-	-
Carnegie Museums	USA	1<br>1 (100.0%)	-	-	1<br>1 (100.0%)

Supplementary Table 9: Summary of a) bird, b) mammal, c) amphibian records contributed to GBIF by different data publishers and used in this study. Data publishers are ordered by decreasing number of contributed data. In the parentheses are percentages of overall data that passed geographic and taxonomic validation and were used in further analyses. Note that we applied a land area threshold of 30% at the 110 km grain, which resulted in the exclusion of some “good” data collected on or near the sea. We also excluded non-breeding ranges. Therefore percentages of excluded records do not necessarily allow conclusions on the quality of data provided by a particular publisher.

Supplementary Notes 1: Species distribution data

Range Data

We considered all species of terrestrial birds (excluding pelagic feeders, N = 9,712)[1], terrestrial mammals (excluding cetaceans, pinnipeds and sirenians; N = 5,270)[2], and amphibians (N = 6,188)[3]. We projected expert based extent-of-occurrence range maps for these 21,170 species[2,4] into an equal area projection and overlaid them with four nested equal-area grids with grain sizes of c. 110 km, 220 km, 440 km, and 880 km, respectively, at the equator. These range maps were originally drawn by species experts based on a variety of data sources, including point records, local inventories, atlas and literature data. We considered a grid cell as occupied by a species, if any portion of its range map overlapped with it, and chose 110 km as the finest resolution to minimize false presences[5-7]. We excluded 110 km grid cells that did not have at least 30% land area unless they included oceanic islands, in order to minimize effects of area and imprecise range maps while keeping most range-restricted species in the analyses. We further excluded grid cells of which the majority of the land area overlapped with mangrove biomes. This led to the exclusion of 51 narrow endemics near coast lines (not included in the above species count). We overlaid the gridded range maps to define expert-opinion species richness.

Point occurrence records

We focused on records aggregated by the Global Biodiversity Information Facility (GBIF) as a representation of international efforts to mobilize biodiversity data into ‘digital accessible information’ (DAI)[8]. GBIF is by far the largest such effort in geographic and taxonomic scope[9,10] and GBIF-facilitated data have been used to assess progress towards Aichi target 19[11]. We received 192,637,611 geo-referenced records for birds, mammals and amphibians from GBIF in October 2012, of which we extracted 192,463,144 records with potentially sensible

geographic coordinates (Longitude: -180° – +180°, Latitude: -90° – +90°) reported with a precision of at least one tenth of a degree. We excluded 8,861,041 records that did not have either a binomial or trinomial scientific name, 278,107 records for which the 'basis of record' field did not indicate 'preserved specimen', 'observation', or 'unknown' (most of which are observation records), and 9,865 records that were reportedly collected before the year 1850, leaving 183,488,598 records. We validated these taxonomically and geographically (see below), which left 157,086,248 records for further analyses.

Taxonomic and geographic validation of records

We then matched the taxonomies of records and range maps. To maximize the amount of records that would pass taxonomic standardization, we combined information on accepted names and synonyms from seven existing taxonomic databases (see below). We accepted species delimitations following ref.[1] for birds, ref.[2] for mammals, and ref.[3] for amphibians. To each accepted species name, we linked further scientific names fully or partly included in the respective species concept from the above and four further databases[2,12–14], including synonyms, subspecies, and common typographical variants. Via this "synonym table", we linked records to the accepted species. We excluded records likely referring to domesticated forms. The synonym tables for the three vertebrate groups, along with a brief guideline of how to use them, are available as Supplementary Dataset 1. We inferred the taxonomic identities of records with ambiguous scientific names (such as *pro parte* synonyms) from spatial overlays with the range maps of all accepted species to which the name could potentially refer. In further analyses, we only used records of which the species identity could be unambiguously determined because they fell inside the gridded range maps (at 110 km grain) of only one accepted species. This led to the exclusion of 13.9 to 29.0% "false" or unclear records (see Supplementary Table 4). By validating localities of records against expert-opinion range maps, we ensure that records are biologically plausible and do not refer to zoo or invasive animals

outside of their native ranges. We note that this approach may lead to the exclusion of "good" records collected outside of range maps if the maps are inaccurate. While coordinate transposition of geographically false records and "fuzzy matching" of names would have decreased the number of excluded records marginally[15,16] this would also have increased the uncertainty associated with the validity of records[15]. Supplementary Table 4 shows results of the geographic and taxonomic validation of records.

Record density and inventory completeness

We overlaid the validated records with the same grids as the range maps. For each grid cell, we then calculated record density as the number of records per 10,000 km^2 land area and inventory completeness as the percentage of expert-opinion species richness documented by records.

Supplementary Notes 2: Geographic and socio-economic variables explaining inventory completeness

We analyzed the relationships of twelve different geographic and socio-economic factors with record density and inventory completeness. These represent a wide range of existing hypotheses that can be categorized into five broader categories: 1) appeal, 2) accessibility, 3) security, 4) international scientific integration, and 5) financial and institutional resources (for details see maps and discussion of variables below). We limited collinearity among predictor variables by only including variables with Pearson's correlation coefficients ≤ 0.7[17].

Most data were available at spatial grains $\leq 0.25°$ and aggregated as arithmetic means for the grid cells. We created a few variables from country-level data sets, namely security, national research funding, integration into scientific activities, and GBIF participation (see below). We assumed that the effects of these factors on biodiversity sampling and data mobilization efforts would be similar throughout a given country, and thus used the same value for each grid cell within the country. For grid cells overlaying several countries, we calculated the arithmetic

mean of the respective country values weighted by the proportion of land area that falls within each country. We based the definition of country boundaries and the calculation of land area on the polygons of the GADM database (www.gadm.org/version1). We assigned disputed areas to the country currently having *de facto* administrative control.

Endemism richness:

Areas with specific biodiversity features are naturally interesting to ecologists and several authors have suggested that collectors frequent areas where they can expect to find many or rare species[18–23]. To test whether there is global support for this "diversity tracking" hypothesis[21], we used endemism richness[24], as it combines aspects of both species richness and species' range-sizes within an assemblage. Endemism richness is calculated as the sum of the inverse global range sizes of all species present in a grid cell. We estimated the range of each species as the sum of 110 km grid cells overlaying the respective range map polygon[2,25]. We assumed a taxonomic focus of most collectors to at least class-level and therefore used avian, mammalian, and amphibian endemism richness, respectively, to predict inventory completeness of the three vertebrate classes. Note that a focus on rare species during sampling[26,27] or a possible emphasis on type specimens during digitization could also lead to range-restricted species being disproportionately represented in mobilized data and thus to data being biased towards high endemism areas.

Mountains:

Mountains could also draw a special attention of collectors because of their scenic beauty or their elevational habitat gradients and, accordingly, high species turnover and the presence of "mountain specialists"[21,22,28–30]. Conversely, it has been reported that mountains are relatively neglected by collecting efforts in some areas due to their poor accessibility[31,32]. To test for effects of mountains on inventory completeness and record density, we calculated the

topographic range within each grid cell as the difference between the minimum and maximum altitude, based on data from the GTOPO-30 digital elevation model[33].

Protected areas:

Protected areas could attract collectors because they may promise "pristine" habitats in otherwise altered landscapes or represent strongholds of rare or sought-after species[23,28–30,34–37]. If developed for ecotourism or management, they may also provide the most straightforward access points to ecosystems[37]. To model the effect of protected areas, we calculated the proportion of the land area in each grid cell covered by protected areas of International Union for Conservation of Nature categories I to IV[38]. Preliminary analyses demonstrated that using an alternative predictor variable based on all[38] protected areas (thus including more protected areas, e.g. from China) did not alter our conclusions.

On-ground accessibility:

Some of the most frequently tested hypotheses regarding sampling bias revolve around the on-ground accessibility of areas to researchers, especially via roads (e.g., the "highway effect"[39] or "road-map effect"[40]). Because the time needed to access an area on the ground has to be traded off against time spent sampling, collectors often choose to sample close to human population centers[19–21,23,28,31,34,35,41–43] or on-ground transportation routes like roads, railways, navigable rivers and coasts lines[20,21,31,34,35,37,39,40,43–47]. These effects have been documented mainly at local to regional spatial scales. While most studies found negative relationships between distance to urban areas and transportation routes,[30] have found that in China, the opposite is true at the county scale, i.e. sampling intensity and inventory completeness are negatively correlated with both road and human population density. To test whether on-ground accessibility influences data availability at the global scale, we used the 'Travel time to major cities' dataset[48], which provides estimates of the time needed to travel to cities with a

population >50,000, and which combines data on urban areas, roads, railroads, navigable rivers, shipping lanes, habitat types, etc. We calculated mean values for every grid cell, and reversed arithmetic signs, so that higher numbers in our index corresponded to greater accessibility.

Proximity to airports:

Since ecologists often have to travel long distances to their study areas, it is possible that regions more accessible by air travel have been better sampled and therefore have higher record density and inventory completeness[31,37]. To estimate the accessibility of areas by air travel, we used data on the locations of >9,300 airports and airfields[49]. Areas close to several airports should be more accessible to researchers, and we therefore calculated the mean distance of every grid cell centroid to the five closest airports. Again, we reversed arithmetic signs to create an index where large values correspond to close proximity to airports.

Proximity to research institutions:

If sampling is mainly carried out by staff of specimen-housing institutions, then time and money constraints could lead collectors to focus on areas nearby their homes or home institutions, and correspondingly, to administrative areas with research institutions being more thoroughly sampled[18,29–31,34,50–52]. This effect has been mostly documented for plants (hence, the "botanist effect" [50]), but it can be hypothesized for any group of organisms.

At the global scale, different aspects complicate testing this hypothesis: First, specimen-housing institutions often have a strong geographical and taxonomic focus. So not all institutions in close proximity to a given grid cell should be considered as potential samplers of its biodiversity. For instance, an institution specializing in bird migrations is unlikely to collect amphibians in a nearby wetland. We therefore created an index based on the distances to those institutions that currently focus or have focused on sampling the respective vertebrate class in

the broader geographic region surrounding a grid cell. For a given focal grid cell and vertebrate class, we identified data publishers (i.e., institutions) that contributed records from within 750 km of the grid cell centroid. We geo-located these publishers (to at least 50 km accuracy) and calculated their distance (in km) to the grid cell centroid. When simply calculating the mean distance to those publishers weighted by their relative contribution, we found that the many large European and North American institutions had an overarching effect on the index, and all grid cells in the southern hemisphere emerged as remote, even if situated in close proximity to "southern" institutions. We therefore calculated the proximity of grid cells to the relevant publishers as the weighted mean of inverse distances or "proximities" (in km; multiplied by 10^8 for easier scaling):

$$10^8 * \sum_{i=0}^{n}(\text{RelContrib}i/\text{D}i)$$

where RelContrib_i is the relative contribution of the i-th publisher to the records from the area and D_i the distance (in km). This index has high values when the majority of data within an area are provided by publishers in close proximity. In preliminary analyses we also calculated the weighted mean of $\log_{10}$-transformed and square root-transformed distances, which yielded very similar results, so we used the best performing index based on AIC.

Our approach differs from that of Amano & Sutherland[53], who tested for the effect of the distance to data aggregators (e.g., the GBIF headquarters in Copenhagen, Denmark) rather than data publishers, and found only a negligible effect for GBIF-enabled data. However, while the big biodiversity data aggregators like GBIF, VertNet, SpeciesLink or eBird provide the infrastructure for linking biodiversity data, they are themselves not responsible for the amount or informational content of the data (this lies with distributed data providers). We therefore excluded data for which the indicated publisher itself is an international data aggregator from the calculation of our index.

Secure conditions:

Human hazards associated with armed conflicts, territorial disputes, low levels of public safety or political instability can discourage scientific activities[54,55] and have been reported or hypothesized to have adverse effects on biodiversity data collection and data administration activities, such that more data are available for areas characterized by secure conditions[20,23,32,53,56–58]. To test this hypothesis, we used the Global Peace Index (GPI)[59], which is probably the most inclusive existing index describing the overall state of security within a country [53]. We note that this index has several drawbacks. First, it is aggregated at the country level, while real levels of security can vary within countries. It is unclear at which spatial scales security levels would deter collecting efforts (i.e., depending on their risk tolerance and detail of available information, foreign collectors could avoid particular low-security parts of a country or entire geo-political regions). As a further drawback, even though we calculated the mean GPI score across several years, the index is only available for the time period between 2008 and 2012 and may not reflect real or perceived security levels in the 1950s through 1980s where many of the specimen records have been collected. In preliminary analyses, we found that an index of the frequency of armed conflicts from 1946 to 2008, created from more fine-scale data[60] was consistently a very poor predictor of record density and inventory completeness for all taxa and spatial grains (results not shown). A third potential drawback is that the GPI is not only based on factors affecting the level of personal safety within a country, but also on the level of militarization, which may be unimportant to collectors. However, potential alternative country-level measures of perceived personal safety that we tested in preliminary analyses ('political stability and absence of violence'[61], 'control of corruption'[61], physician density[62]) were highly collinear with the GPI, so we restricted our main analyses to this measure. Because high GPI values stand for low levels of security, we reversed arithmetic signs of GPI values with after $\log_{10}$-transformation to create an index of secure conditions, and

accordingly hypothesized a positive relationship with both record density and inventory completeness.

Scientific activities:

Low levels of record density and inventory completeness in specific countries may also be due to a lack of scientific capacity or expertise[23,56], or be the result of a delayed start and poor international integration into the communication of ecological science due to linguistic reasons [53]. Conversely, countries whose researchers actively engage in the communication of science through peer-reviewed publication and are internationally well-integrated through collaborations may also mobilize and share more data via international networks like GBIF. To estimate this integration of a country into international scientific communication and collaborations (or "globalization of science"[53]), we used data on peer-reviewed primary literature from the *SCImago Journal & Country Rank*, which assembles publication ranks based on *Elsevier*'s *Scopus* database[63]. We extracted the H-index for every country based on peer-reviewed papers published between 1996 and 2011 in the field 'Ecology, Evolution, Behavior and Systematics', and multiplied it with the proportion of papers resulting from international collaborations, i.e., with authors' home institutions situated in at least two countries.

GBIF participation:

Although GBIF represents by far the largest international effort facilitating access to point records, many data holders currently do not share their data or only make them accessible via smaller, mostly national networks. Not sharing available biodiversity data internationally due to, e.g., political, economic, or legal reasons has been identified as a key factor limiting scientific progress[64], and the availability of readily accessible biodiversity data from many parts of the world[15,65]. One of the main strategic goals of GBIF for the coming years therefore is

winning the support and cooperation of as yet non-participating countries[66]. To test whether cooperation of countries with GBIF is important in limiting biodiversity information from their territories, we used the proportion of the land area within each grid cell that is covered by a GBIF-participating country (as of April 2013, information from GBIF website).

National research funding:

Locally available financial resources have been shown to be an important factor limiting scientific activities in developing countries[67,68] and are thus a frequently hypothesized reason for low availability of biodiversity data[36,47,52,53,56,69]. To estimate the financial resources that are potentially available for biodiversity research, we gathered information on the per capita gross domestic expenditure (in purchase power parity dollars) on research and development (GERD)[70,71]. Most other studies have used measures of economic activity such as per capita GDP. Although biodiversity-related funding only makes up a tiny fraction of GERD, research and development spending is generally more closely tied to scientific activities and scientific output than GDP-based measures[67], and we believe it to be a better proxy for resources that are available for biodiversity studies. We assumed that research grants are mostly available from national funding institutions, and that every grid cell within a country has a similar likelihood of obtaining money for biodiversity data collection and mobilization. We therefore assigned the same GERD value to every grid cell within a country. We restricted our models to those grid cells with at least 70% of their land area covered by countries with available GERD data, which led to the exclusion of some grid cells, particularly in Africa and Asia (see maps of included grid cells and predictor variables above). Preliminary analyses in which we replaced GERD by per capita GDP[72] as an estimate of research funding and thus included more grid cells showed that it was indeed a poorer predictor of both record density and inventory completeness, but otherwise did not alter our conclusions.

Research funding of institutions:

Data collection within a particular area as well as their mobilization is often carried out by staff of foreign research institutions. Therefore research funding available in the countries of those institutions that actually contribute data from that area may be a more plausible limiting factor for DAI than locally available funding. A survey on the challenges involved in specimen digitization among the natural history community[73] found funding to institutions (or related institutional aspects such as technical infrastructure or number and expertise of staff) to be the main factor limiting specimen digitization and biodiversity data mobilization (see also[56]). To test whether this factor limits record density and inventory completeness globally, we created an index based on GERD data in data publisher countries (see above, GERD data available for all 31 countries with data publishers that have contributed records used in this study). We linked to every data publisher the GERD value (in purchase power parity dollars) of the country where it is located. For each grid cell, we then calculated the mean GERD of data publishers, weighted by their relative contribution to the records from the respective grid cell:

$$\sum_{i=0}^{n}(\text{RelContrib}i * \text{GERD}i)$$

where RelContrib_i is the relative contribution of the *i*-th publisher to the records from the grid cell and GERD_i the GERD in the country where the *i*-th publisher is located. We acknowledge that research institutions within a given country may differ in their ability to attract funding, and chances of securing funding for data mobilization may depend more on the existence of specific funding programs (such as the National Science Foundation's 'Advancing Digitization of Biodiversity Collections' initiative) than on among-country differences in GERD.

Publisher size:

By definition, larger research institutions have larger quantities of data. Additionally, they often have more resources available for sampling and curatorial activities as well as more and highly

specialized staff, combining a greater variety of research foci and taxonomic expertise than smaller institutions[74]. Some large North American and European institutions are also reported to have more important collections from Africa, Asia and South America than smaller local institutions because they were involved in extensive biodiversity inventory programs in those regions[75]. Accordingly, data provided by these institutions should include specimens of more and rarer species[23,26,75,76], leading to higher levels of inventory completeness in regions where they are or have been active. On the other hand, Chauvel *et al.*[77] also highlight the value of specific information added only by smaller institutions. Yesson *et al.*[15] suggested that a focus on large institutions would most efficiently fill gaps in global, digital accessible information, and a focus on the largest North American and European collections is part of GBIF's strategic plan for 2012-2016[66]. To test whether the size of contributing institutions is limiting record density and inventory completeness in their focal areas, we created an index based on the mean size of institutions that are active within a particular grid cell, weighted by their relative contributions:

$$\sum_{i=0}^{n}(\mathrm{RelContrib}i * \mathrm{V}i)$$

where $\mathrm{RelContrib}_i$ is the relative contribution of the *i*-th publisher to the records from the grid cell and V_i the total data volume that the *i*-th publisher contributed to GBIF (as of Oct 2012). We acknowledge that different institutions have advanced to different degrees in terms of mobilizing their data into DAI[78], which could potentially bias our estimation of publisher size. However, no reliable information of the size of all institutions that contribute data to GBIF is currently available (compare[78]). Record counts of data publishers are summarized in Supplementary Table 9.

Supplementary Notes 3: Statistical methods

We compared the mean completeness among regions using max-*t* tests[79], and *P*-values were adjusted to geographically effective degrees of freedom following Dutilleul[80].

We investigated the effects of the predictor variables on record density and inventory completeness with simple and multiple regression analyses and built regression models separately for amphibians, birds and mammals at each of four spatial grains (110 km, 220 km, 440 km, 880 km). Because some explanatory variables were calculated using information from the records (e.g., 'Proximity to institutions'), we only included grid cells with at least one record (see Supplementary Figure 4 and Supplementary Table 5).

Before entering the models, record density as well as all predictor variables were $\log_{10}(x + k)$-transformed, with a variable-specific constant k added to each value x, so that the smallest value before $\log_{10}$-transformation equaled 1[81]. Predictor variables with values bound between 0 and 1 ('Protected areas', 'GBIF participation') were arcsine-square root-transformed before $\log_{10}$-transformation. To account for bias due to area-effects, we included the $\log_{10}$-transformed land area within each grid cell as a covariate in all multiple regression models (highly significant in all cases).

We modeled effects on record density with non-spatial linear models (ordinary least squares) as well as "spatial" simultaneous autoregressive models (SAR) of the error type, which account for spatial autocorrelation (SAC) in the residuals[82], using functions from the R package *spdep*. We used non-spatial and spatial GLMs with a binomial distribution and a logit link to model effects on inventory completeness, which entered the model as a composite variable: *cbind*('species covered by GBIF', 'species not covered but presumed present') in *R* terminology. The spatial GLMs were formed by first running a given non-spatial model, and then calculating the 'residuals autocovariate' (RAC) using the *spdep*-function *autocov_dist*, based on a specific neighborhood structure (a list of neighborhood cells to each grid cell) and the residuals of the non-spatial model. The RAC was then entered in the model as a covariate

and accounted for SAC in the model residuals[83], similar to an error-type SAR. We used the global Moran's I test to determine the degree of SAC[81]. Significant SAC in model residuals often persisted in the spatial models but was reduced by about one order of magnitude compared to non-spatial models (see Moran's I values in Supplementary Table 6).

To represent simple associations of predictor and response variables, we ran single-predictor models (non-spatial and not including log-transformed land area as a covariate) and report the coefficient of determination and deviance explained, respectively, for OLS and GLMs (Supplementary Figure 3, Supplementary Tables 6-8). We assessed model fit of the minimum adequate models (MAMs) as the % deviance explained (D^2) in the case of RAC models (spatial binomial GLMs; Supplementary Table 6b) and as Pseudo-R^2 in the case of SAR models (Supplementary Table 6b). To test for potential country effects that would remain after controlling for the main 12 predictor variables, we added countries as an additional factor to the spatial MAMs and assessed the increase in model fit (Supplementary Table 7).

Long computation times due to the large amount of predictor variables and high numbers of grid cells made it unfeasible to run all possible spatial models. For both inventory completeness and sampling effort, we instead first ran all possible non-spatial multiple-regression models. We then identified all model subsets that would likely be among the minimum adequate spatial models (with a ΔAIC <10 to the MAM) and only re-ran those models as spatial models.

Both SAR and RAC models require defining a neighborhood structure that defines the distance over which SAC occurs in model residuals. For each grain, we identified the range of distances that would define a neighborhood structure with a median of 8 (~ one cell row) to 24 (~ two cell rows) neighbor cells around focal cells. We then re-ran all candidate model subsets as spatial models for each of five different neighborhood structures based on five distances within that range: for the 110 km grain 200, 250, 300, 350, and 400 km, for the 220 km grain 400, 500, 600, 700, and 800 km, for the 440 km grain 800, 1,000, 1,200, 1,400, and 1,600 km, and for the 880 km grain 1,600, 2,100, 2,600, 3,100, and 3,600 km.

We also investigated interactions and non-linear effects, and although many were significant, accounting for them did not greatly alter model fit or parameter estimates of the main effects in preliminary analyses. To maintain as much simplicity as possible with twelve predictor variables, we therefore decided to focus on the main effects.

Relative importance of predictor variables

For each taxon and grain, we identified the minimum adequate spatial models based on AIC scores. We report the standardized coefficient (β) of the most strongly supported spatial MAM (i.e., with lowest AIC score) in Fig. 3 and Supplementary Fig. 5, and where applicable, the range of the standardized coefficient among all potential spatial MAMs (with ΔAIC <2 to the lowest AIC score) in Supplementary Tables 6-8. Where the model with the lowest AIC score did not include a factor, we report the standardized coefficient of the "second-best" model (if among the potential MAMs, Supplementary Tables 8). If none of the potential MAMs had a particular factor, it was left blank in Fig. 3 and Supplementary Fig. 5.

As an alternative measure of relative importance, and considering all possible subsets of the full non-spatial model as experimental units, we carried out ANOVAs with a response variable consisting of the AIC scores of all possible models and predictor variables formed as dummy-variables coding for every factor whether or not it is in the respective model. The percentage of the total Sums of Squares (% SS) attributable to each factor corresponds to their relative importance (compare[84,85]).

Supplementary Notes 4: Limitations of this study

Biodiversity data sources

With GBIF and the many integrated data sources (see Supplementary Table 9) we cover by far the largest share of global digital accessible information on biodiversity. However, several global and regional data mobilization initiatives provide access to digital data, but do not

currently make their data accessible via GBIF. Additionally, several regions have digital or non-digital data that are not shared.

In this study, we focused on information on species distributions as a particularly essential biodiversity variable[6], that is a prerequisite for more nuanced conservation strategies targeting critical or declining populations, or associated ecosystem services. However, we acknowledge that several initiatives address data types that inform about other aspects of critical relevance for conservation, such as species' abundances[86], ranging behavior[87], or conservation status[2]. Such datasets require equally systematic assessments and prioritizations in order to effectively proceed towards Aichi target 19.

Potential biases

The extremely large numbers of bird observation records (85.6% of all validated records) result in a vast overrepresentation of birds in our dataset (152M records/9,712 species) compared to mammals (3.4M records/5,270 species) and amphibians (1.3M records/6,188 species). This overrepresentation means that model results for birds may be more reliable than those for mammals and amphibians. However, record quantities upon which mammalian and amphibians inventory completeness values are based are still in the low millions, sufficiently high to support our analyses and conclusions. Birds' over-representation in species and record numbers does not bias our conclusions on overall important limiting factors for inventory completeness, as we modeled effects separately for each of the three vertebrate groups and emphasized factors that consistently emerged as relatively important irrespective of vertebrate group, spatial grain, and evaluation metric (hence our stress on 'Proximity to research institutions' and 'GBIF participation'). In cases were important effects only emerged for one or two vertebrate groups, we make that clear.

Another factor that might affects our analyses is that the ratio between observation records and specimen records is much higher in birds (0.01) compared to mammals (1.4) and amphibians

(2.8). Thus, models for birds mainly explain patterns in observation records. However, as these observation records are readily accessible to anyone wishing to use bird records in research or conservation, we see no basis for excluding them. Quite the contrary, focusing analyses more on specimen-derived records (to more closely approximate the observation/specimen ratios in mammals and amphibians) would bias analyses towards a small and potentially unrepresentative fraction of available occurrence information.

Finally, grid cells with zero records (excluded from modeling analyses) are geographically biased. Certain regions like North Africa and Central Asia have particularly high proportions of cells that are not included in models, especially at fine spatial grains, and may not be well-informed by our models (see Supplementary Fig. 4, Supplementary Table 5). However, even at fine grains, included grid cells are globally distributed and poorly-covered cells are best-represented in absolute terms (see histograms in Supplementary Fig. 4). Therefore, our models should be informative about the factors limiting inventory completeness globally, including in poorly-covered regions.

Explanatory variables

A general shortcoming of our study is that we had to rely on fairly recent socio-economic datasets. We investigated time series of collected data volumes per 5-year period which showed that the majority of records (i.e., including both observation and specimen records) have been collected in recent decades, but specimens in particular were often collected several decades ago (median recording year for amphibians: 1979; for mammals: 1989; for birds: 2007). We implicitly assumed that among-region differences in factors relating to field sampling, like on-ground accessibility, protected areas, and levels of research funding, have on average been similar at the times when data were collected. As digitization and sharing of these records happened mostly within the last decade, record age does not affect our conclusions regarding

the main factors currently limiting DAI. However, spatiotemporal changes in sampling activities in relation to historical factors (e.g. roads, reserves) is a needed area of further study. With the factors included in this study, we attempted to cover a wide range of established hypotheses on the drivers of data bias and inventory completeness in global DAI. However, we note that original collection, digitization, mobilization, and sharing of data may be influenced by further contemporary and historical socio-economic factors, such as political systems and agendas, levels of bureaucracy and international cooperation, policies of funding agencies, and legal aspects[20,64,73,88], information technological capacity[89], lingua franca[43,53], colonial history[37,75,90], traditions of natural history institutions and personal preferences of collectors and curators[91], as well as attitudes of countries and data owners towards data-sharing[92,93]. Most of these effects are difficult to quantify, and existing country-level datasets are often highly collinear. Some of these effects, however, may become visible in the form of country effects, not least because data mobilization to GBIF is organized via national nodes. However, many countries have experienced extreme political transitions as well as changes in their sovereign territory over the course of time when data have been collected, and effects of modern country identities on record density and inventory completeness may be difficult to interpret for many parts of the world. We therefore decided not to perform hierarchical mixed effects models with countries as a random factor, but instead only assess the increase in model fit if a 'country' factor was added to the minimum adequate multi-predictor models.

Supplementary References:

1. BirdLife International. *Taxonomic Checklist of the Birds of the World version 3 (2011). Available at http://www.birdlife.org.* (2011).

2. IUCN. *IUCN Red List of Threatened Speciess. Version 2010.4. Available at http://www.iucnredlist.org.* (2010).

3. Frost, D. R. Amphibian Species of the World: an Online Reference. Version 5.5 (2012). American Museum of Natural History, New York, USA. (2012). *Electronic Database accessible at http://research.amnh.org/herpetology/amphibia/index.html* (2012).

4. Jetz, W., Wilcove, D. S. & Dobson, A. P. Projected impacts of climate and land-use change on the global diversity of birds. *PLoS Biol.* **5,** e157 (2012).

5. Hurlbert, A. H. & Jetz, W. Species richness, hotspots, and the scale dependence of range maps in ecology and conservation. *Proc. Natl. Acad. Sci. U. S. A.* **104,** 13384–9 (2007).

6. Hawkins, B. A., Rueda, M. & Rodríguez, M. Á. What Do Range Maps and Surveys Tell Us About Diversity Patterns? *Folia Geobot.* **43,** 345–355 (2008).

7. Hortal, J. Uncertainty and the measurement of terrestrial biodiversity gradients. *J. Biogeogr.* **35,** 1335–1336 (2008).

8. Sousa-Baena, M. S., Garcia, L. C. & Peterson, A. T. Completeness of digital accessible knowledge of the plants of Brazil and priorities for survey and inventory. *Divers. Distrib.* **20,** 369–381 (2014).

9. Edwards, J. L. Interoperability of Biodiversity Databases: Biodiversity Information on Every Desktop. *Science* **289,** 2312–2314 (2000).

10. Graham, C. H., Ferrier, S., Huettman, F., Moritz, C. & Peterson, A. T. New developments in museum-based informatics and applications in biodiversity analysis. *Trends Ecol. Evol.* **19,** 497–503 (2004).

11. Tittensor, D. P. *et al.* A mid-term analysis of progress toward international biodiversity targets. *Science* **346,** 241–244 (2014).

12. Wilson, D. E. & Reeder, D. M. Mammal Species of the World. A Taxonomic and Geographic Reference (3rd ed). *Available at http://www.press.jhu.edu* (2005).

13. Lepage, D. Avibase - The World Bird Database v. 2012. *Available at http://avibase.bsc-eoc.org* (2012).

14. ITIS Global Orrell T. (custodian). ITIS Global: The Integrated Taxonomic Information System (version Apr 11). In: Species 2000 & ITIS Catalogue of Life, 2012 Annual Checklist (Bisby F., Roskov Y., Culham A., Orrell T., Nicolson D., Paglinawan L., Bailly N., Appeltans W., Kirk P., Bourgoin. (2012).

15. Yesson, C. *et al.* How global is the global biodiversity information facility? *PLoS One* **2,** e1124 (2007).

16. Otegui, J. Quality and Fitness-for-use Assessments on the Primary Data Indexed at the Global Biodiversity Information Facility (GBIF). Dissertation Thesis, 390p. (2012).

17. Dormann, C. F. *et al.* Collinearity: a review of methods to deal with it and a simulation study evaluating their performance. *Ecography* **36,** 27–46 (2013).

18. Dennis, R. L. H. & Thomas, C. D. Bias in butterfly distribution maps : the influence of hot spots and recorder's home range. *J. Insect Conserv.* **4,** 73–77 (2000).

19. Kier, G. *et al.* Global patterns of plant diversity and floristic knowledge. *J. Biogeogr.* **32,** 1107–1116 (2005).

20. Küper, W., Sommer, J. H., Lovett, J. C. & Barthlott, W. Deficiency in African plant distribution data – missing pieces of the puzzle. *Bot. J. Linn. Soc.* **150,** 355–368 (2006).

21. Lobo, J. M., Romo, H. & García-Barros, E. Identifying recorder-induced geographic bias in an Iberian butterfly database. *Ecography* **6,** 873–885 (2006).

22. Soria-Auza, R. W. & Kessler, M. The influence of sampling intensity on the perception of the spatial distribution of tropical diversity and endemism: a case study of ferns from Bolivia. *Divers. Distrib.* **14,** 123–130 (2008).

23. Boakes, E. H. *et al.* Distorted views of biodiversity: spatial and temporal bias in species occurrence data. *PLoS Biol.* **8,** e1000385 (2010).

24. Kier, G. & Barthlott, W. Measuring and mapping endemism and species richness: a new methodological approach and its application on the flora of Africa. *Biodivers. Conserv.* **10,** 1513–1529 (2001).

25. Jetz, W., McPherson, J. M. & Guralnick, R. P. Integrating biodiversity distribution knowledge: toward a global map of life. *Trends Ecol. Evol.* **27,** 151–159 (2012).

26. Guralnick, R. & Van Cleve, J. Strengths and weaknesses of museum and national survey data sets for predicting regional species richness: comparative and combined approaches. *Divers. Distrib.* **11,** 349–359 (2005).

27. Ter Steege, H., Haripersaud, P. P., Bánki, O. S. & Schieving, F. A model of botanical collectors' behavior in the field: never the same species twice. *Am. J. Bot.* **98,** 31–7 (2011).

28. Parnell, J. A. N. *et al.* Plant collecting spread and densities: their potential impact on biogeographical studies in Thailand. *J. Biogeogr.* **30,** 193–209 (2003).

29. Sánchez-Fernández, D., Lobo, J. M., Abellán, P., Ribera, I. & Millán, A. Bias in freshwater biodiversity sampling: the case of Iberian water beetles. *Divers. Distrib.* **14,** 754–762 (2008).

30. Yang, W., Ma, K. & Kreft, H. Environmental and socio-economic factors shaping the geography of floristic collections in China. *Glob. Ecol. Biogeogr.* **23,** 1284–1292 (2014).

31. Funk, V. A., Zermoglio, M. F. & Nasir, N. Testing the use of specimen collection data and GIS in biodiversity exploration and conservation decision making in Guyana. *Biodivers. Conserv.* **8,** 727–751 (1999).

32. Funk, V. A. & Richardson, K. S. Systematic data in biodiversity studies: use it or lose it. *Syst. Biol.* **51,** 303–16 (2002).

33. US Geological Survey. GTOPO30. *Available at http://www1.gsi.go.jp/geowww/globalmap-gsi/gtopo30/gtopo30.html.* (1996).

34. Freitag, S., Hobson, C., Biggs, H. C. & Jaarsveld, A. S. Testing for potential survey bias: the effect of roads, urban areas and nature reserves on a southern African mammal data set. *Anim. Conserv.* **1,** 119–127 (1998).

35. Reddy, S., Dávalos, L. M. & Da, L. M. Geographical sampling bias and its implications for conservation priorities in Africa. *J. Biogeogr.* **30,** 1719–1727 (2003).

36. Martin, L. J., Blossey, B. & Ellis, E. Mapping where ecologists work: biases in the global distribution of terrestrial ecological observations. *Front. Ecol. Environ.* **10,** 195–201 (2012).

37. Ballesteros-Mejia, L., Kitching, I. J., Jetz, W., Nagel, P. & Beck, J. Mapping the biodiversity of tropical insects: species richness and inventory completeness of African sphingid moths. *Glob. Ecol. Biogeogr.* **22,** 586–595 (2013).

38. IUCN and UNEP. *The World Database on Protected Areas (WDPA). UNEP-WCMC. Cambridge, UK.* (2009).

39. Soberón, J. M., Llorente, J. B. & Oñate, L. The use of specimen-label databases for conservation purposes : an example using Mexican Papilionid and Pierid butterflies. *Biodivers. Conserv.* **9,** 1441–1466 (2000).

40. Crisp, M. D., Laffan, S., Linder, H. P. & Monro, A. Endemism in the Australian Flora. *J. Biogeogr.* **28,** 183–198 (2001).

41. Osborne, P. E. & Tigar, B. J. Interpreting Bird Atlas Data Using Logistic Models: An Example From Lesotho, Southern Africa. *J. Appl. Ecol.* **29,** 55–62 (1992).

42. Diniz-Filho, J. A. F. *et al.* Macroecological correlates and spatial patterns of anuran description dates in the Brazilian Cerrado. *Glob. Ecol. Biogeogr.* **14,** 469–477 (2005).

43. Schulman, L., Toivonen, T. & Ruokolainen, K. Analysing botanical collecting effort in Amazonia and correcting for it in species range estimation. *J. Biogeogr.* **34,** 1388–1399 (2007).

44. Gioia, P. & Pigott, J. P. Biodiversity assessment : a case study in predicting richness from the potential distributions of plant species in the forests of south-western Australia. *J. Biogeogr.* **27,** 1065–1078 (1998).

45. Hijmans, R. J. *et al.* Assessing the Geographic Representativeness of Genebank Collections: the Case of Bolivian Wild Potatoes. *Conserv. Biol.* **14,** 1755–1765 (2000).

46. Sciences, L., Kadmon, R., Farber, O. & Danon, A. Effect of roadside bias on the accuracy of predictive maps produced by bioclimatic models. *Ecol. Appl.* **14,** 401–413 (2004).

47. Newbold, T. Applications and limitations of museum data for conservation and ecology, with particular attention to species distribution models. *Prog. Phys. Geogr.* **34,** 3–22 (2010).

48. Nelson, A. Travel time to major cities: A global map of Accessibility. Global Environment Monitoring Unit - Joint Research Centre of the European Commission, Ispra Italy. *Available at http://gem.jrc.ec.europa.eu/* (2008).

49. Partow, A. The Global Airport Database. Release Version 0.0.1. *Available at http://www.partow.net/miscellaneous/airportdatabase/* (2003).

50. Moerman, D. E. & Estabrook, G. F. The botanist effect: counties with maximal species richness tend to be home to universities and botanists. *J. Biogeogr.* **33,** 1969–1974 (2006).

51. Pautasso, M. & McKinney, M. L. The botanist effect revisited: plant species richness, county area, and human population size in the United States. *Conserv. Biol.* **21,** 1333–40 (2007).

52. Ahrends, A. *et al.* Funding begets biodiversity. *Divers. Distrib.* **17,** 191–200 (2011).

53. Amano, T., Sutherland, W. J. & B, P. R. S. Four barriers to the global understanding of biodiversity conservation: wealth, language, geographical location and security. *Proc. R. Soc. B Biol. Sci.* **280,** 20122649 (2013).

54. Bonfoh, B. *et al.* Research in a war zone. *Nature* **474,** 569–71 (2011).

55. Brito, J. C. *et al.* Unravelling biodiversity, evolution and threats to conservation in the Sahara-Sahel. *Biol. Rev. Camb. Philos. Soc.* **89,** 215–231 (2013).

56. Collen, B., Ram, M., Zamin, T. & Mcrae, L. The tropical biodiversity data gap: addressing disparity in global monitoring. *Trop. Conserv. Sci.* **1,** 75–88 (2008).

57. Otegui, J., Ariño, A. H., Encinas, M. a & Pando, F. Assessing the Primary Data Hosted by the Spanish Node of the Global Biodiversity Information Facility (GBIF). *PLoS One* **8,** e55144 (2013).

58. Hortal, J., Jiménez-Valverde, A., Gómez, J. F., Lobo, J. M. & Baselga, A. Historical bias in biodiversity inventories affects the observed environmental niche of the species. *Oikos* **117,** 847–858 (2008).

59. Institute for Economics and Peace. Global peace index 2008-2012. Sydney, Australia: Institute for Economics and Peace. *Available at http://www.visionofhumanity.org/*. (2012).

60. Tollefsen, A. F., Strand, H. & Buhaug, H. PRIO-GRID: A unified spatial data structure. *J. Peace Res.* **49,** 363–374 (2012).

61. The World Bank. Worldwide Governance Indicators. *Available at http://info.worldbank.org/governance/wgi/*. (2012).

62. World Health Organization. Global Atlas of the Health Workforce. *Available at http://apps.who.int/globalatlas/*. (2012).

63. SCImago. SJR — SCImago Journal & Country Rank. *Retrieved March 12, 2013, from http://www.scimagojr.com*. (2007).

64. U.S. National Committee for CODATA. Bits of Power - Issues in Global Access to Scientific Data. NATIONAL ACADEMY PRESS 249 (1997). *PDF available at http://www.nap.edu/catalog/5504/bits-of-power-issues-in-global-access-to-scientific-data* (2015).

65. Thomas, C. Biodiversity Databases Spread, Prompting Unification Call. *Science* **324,** 1632–1633 (2009).

66. GBIF. GBIF Strategic Plan 2012-2016: Seizing the future. *Available at http://www.gbif.org/orc/?doc_id=2792.* (2011).

67. May, R. M. The Scientific Wealth of Nations. *Science* **275,** 793–796 (1997).

68. King, D. A. The scientific impact of nations - What different countries get for their research spending. *Nature* **430,** 311–316 (2002).

69. Soberón, J. M. & Peterson, A. T. Biodiversity informatics: managing and applying primary biodiversity data. *Philos. Trans. R. Soc. Lond. B. Biol. Sci.* **359,** 689–98 (2004).

70. UNESCO Institute for Statistics (UIS). Science and technology report. *Available at http://www.uis.unesco.org/ScienceTechnology/Pages/research-and-development-statistics.aspx*. (2012).

71. Palmer, L. Show me the money. *Nat. Clim. Chang.* **1,** 376–380 (2011).

72. CIA. GDP - per capita (PPP), The World Factbook. *Available at https://www.cia.gov/library/publications/the-world-factbook/*. (2013).

73. Vollmar, A. *et al.* Natural history specimen digitization: challenges and concerns. *Biodivers. Informatics* **1,** 93–112 (2010).

74. Poliseli, L. & Christoffersen, M. L. in *Sociological Landscape - Theories, Realities and Trends* (ed. Erasga, D.) (2012). *Available at http://cdn.intechopen.com/pdfs-wm/34145.pdf* (2012).

75. Lavoie, C. Biological collections in an ever changing world: Herbaria as tools for biogeographical and environmental studies. *Perspect. Plant Ecol. Evol. Syst.* **15,** 68–76 (2013).

76. Longino, J. T., Coddington, J. & Colwell, R. K. The ant fauna of a tropical rain forest: estimating species richness three different ways. *Ecology* **83,** 689–702 (2002).

77. Chauvel, B., Dessaint, F., Cardinal-Legrand, C. & Bretagnolle, F. The historical spread of Ambrosia artemisiifolia L. in France from herbarium records. *J. Biogeogr.* **33,** 665–673 (2006).

78. Ariño, A. H. A. & Riño, A. R. H. A. Approaches to estimating the universe of natural history collections data. *Biodivers. Informatics* **7,** 81–92 (2010).

79. Herberich, E., Sikorski, J. & Hothorn, T. A robust procedure for comparing multiple means under heteroscedasticity in unbalanced designs. *PLoS One* **5,** e9788 (2010).

80. Dutilleul, P. Modifying the t test for assessing the correlation between two spatial processes. *Biometrics* **49,** 305–314 (1993).

81. Belmaker, J. & Jetz, W. Cross-scale variation in species richness-environment associations. *Glob. Ecol. Biogeogr.* **20,** 464–474 (2011).

82. Kissling, W. D. & Carl, G. Spatial autocorrelation and the selection of simultaneous autoregressive models. *Glob. Ecol. Biogeogr.* **17,** 59–71 (2007).

83. Crase, B., Liedloff, A. C. & Wintle, B. A. A new method for dealing with residual spatial autocorrelation in species distribution models. *Ecography* **35,** 879–888 (2012).

84. Dormann, C. F., Purschke, O., García Márquez, J. R., Lautenbach, S. & Schröder, B. Components of uncertainty in species distribution analysis: a case study of the Great Grey Shrike. *Ecology* **89,** 3371–86 (2008).

85. Thibaud, E., Petitpierre, B., Broennimann, O., Davison, A. C. & Guisan, A. Measuring the relative effect of factors affecting species distribution model predictions. *Methods Ecol. Evol.* **5,** 947–955 (2014).

86. NERC Centre for Population Biology - Imperial College. The Global Population Dynamics Database Version 2. (2010). *Available at http://www.sw.ic.ac.uk/cpb/cpb/gpdd.html* (2010).

87. Wikelski, M. & Kays, R. Movebank: archive, analysis and sharing of animal movement data. World Wide Web electronic publication. (2015). *Available at www.movebank.org* (2015).

88. Schäfer, A., Dallmeier-Tiessen, S., Pfeiffenberger, H. & et al. (The ODE Project). Ten Tales of Drivers and Barriers in Data Sharing. *Available at http://epic.awi.de/.* (2011).

89. Ariño, A. H., Chavan, V. & King, N. The Biodiversity Informatics Potential Index. *BMC Bioinformatics* **12,** S4 (2011).

90. Figueiredo, E. & Smith, G. F. The colonial legacy in African plant taxonomy. *S. Afr. J. Sci.* **106,** 1–4 (2010).

91. Pyke, G. H. & Ehrlich, P. R. Biological collections and ecological/environmental research: a review, some observations and a look to the future. *Biol. Rev. Camb. Philos. Soc.* **85,** 247–66 (2010).

92. Van Panhuis, W. G. *et al.* A systematic review of barriers to data sharing in public health. *BMC Public Health* **14,** 1144 (2014).

93. Enke, N. *et al.* The user's view on biodiversity data sharing - Investigating facts of acceptance and requirements to realize a sustainable use of research data. *Ecol. Inform.* **11,** 25–33 (2012).

94. Olson, D. M. *et al.* Terrestrial Ecoregions of the World: A New Map of Life on Earth. *Bioscience* **51,** 933–938 (2001).
